# *Mdga2* deficiency leads to an aberrant activation of BDNF/TrkB signaling that underlies autism-relevant synaptic and behavioral changes in mice

**DOI:** 10.1371/journal.pbio.3003047

**Published:** 2025-04-01

**Authors:** Dongdong Zhao, Yuanhui Huo, Naizhen Zheng, Xiang Zhu, Dingting Yang, Yunqiang Zhou, Shengya Wang, Yiru Jiang, Yili Wu, Yun-wu Zhang

**Affiliations:** 1 Institute of Aging, Key Laboratory of Alzheimer’s Disease of Zhejiang Province, Wenzhou Medical University, Wenzhou, Zhejiang, China; 2 Xiamen Key Laboratory of Brain Center, The First Affiliated Hospital of Xiamen University, and Fujian Provincial Key Laboratory of Neurodegenerative Disease and Aging Research, Institute of Neuroscience, School of Medicine, Xiamen University, Xiamen, Fujian, China; Institute for Basic Science, KOREA, REPUBLIC OF

## Abstract

Memprin/A5/mu (MAM) domain containing glycosylphosphatidylinositol anchor 2 (MDGA2) is an excitatory synaptic suppressor and its mutations have been associated with autism spectrum disorder (ASD). However, the detailed physiological function of MDGA2 and the mechanism underlying MDGA2 deficiency-caused ASD has yet to be elucidated. Herein, we not only confirm that *Mdga2*^ +/−^ mice exhibit increased excitatory synapse transmission and ASD-like behaviors, but also identify aberrant brain-derived neurotrophic factor/tyrosine kinase B (BDNF/TrkB) signaling activation in these mice. We demonstrate that MDGA2 interacts with TrkB through its memprin/A5/mu domain, thereby competing the binding of BDNF to TrkB. Both loss of MDGA2 and the ASD-associated MDGA2 V930I mutation promote the BDNF/TrkB signaling activity. Importantly, we demonstrate that inhibiting the BDNF/TrkB signaling by both small molecular compound and MDGA2-derived peptide can attenuate the increase of α-amino-3-hydroxy-5-methyl-4-isoxazole propionic acid (AMPA) receptor-mediated excitatory synaptic activity and social deficits in MDGA2-deficient mice. These results highlight a novel MDGA2-BDNF/TrkB-dependent mechanism underlying the synaptic function regulation, which may become a therapeutic target for ASD.

## Abstract 2

MDGA2 mutations are associated with autism spectrum disorder (ASD) but the underlying mechanisms are poorly understood. In this study, the authors show that MDGA2 competes with BDNF, thereby constraining TrkB signaling, and that aberrant TrkB activation caused by MDGA2-deficiency is crucial for ASD-related synaptic and behavioral phenotypes.

## Introduction

Autism spectrum disorder (ASD), a complex neurodevelopmental disorder with onset in early childhood, is characterized by reduced social interaction, increased stereotypic repetitive behavior, and altered cognition. The prevalence of ASD has increased significantly in recent years, with approximately 1% of the world population considered to have the disorder [1–4]. Despite growing efforts devoted in this field, the etiology of ASD has yet to be fully elucidated.

Genetic studies have identified multiple genes associated with ASD. A majority of these genes such as *SHANKs*, *DLG4*, *NRXNs*, and *NLGNs*, encode synaptic proteins, suggesting that synaptic dysfunction is a major cause of ASD [4–7]. So far, identified genes only explain a portion of ASD occurrence. Identifying additional ASD-associated genes and revealing the underlying mechanisms should provide new insights into the pathogenesis of ASD and its treatment strategies. Recently, loss-of-function and missense mutations in the synapse-associated *MDGA2* (memprin/A5/mu [MAM] domain containing glycosylphosphatidylinositol anchor 2) gene, including truncations covering the MAM domain of MDGAs and a V930I mutation have been identified in ASD patients [8,9]. MDGA2 and its homolog MDGA1 were found to interact with neuroligins for blocking neurexin (NRXN) binding, so that they can suppress neuroligin synaptogenic activity [10–16]. Some studies also suggested that MDGA2 can negatively regulate the neuroligin 1 (NLGN1)-NRXN synaptic pathway to suppress excitatory synapse development and its deficiency leads to elevated excitatory synaptic activity and ASD-like behaviors in mice [17–19]. However, whether MDGA2 deficiency leads to ASD also through other mechanisms remains unknown.

Growing evidence indicates that dysregulation of brain-derived neurotrophic factor (BDNF), including its abnormal protein levels and single nucleotide variations, is involved in ASD [20–23]. BDNF mediates downstream signaling pathways through its high-affinity binding to the tyrosine kinase B (TrkB) receptor. Activation of the BDNF/TrkB signaling triggers the downstream Akt-mammalian target of rapamycin (mTOR) pathway and leads to over-translation of synaptic proteins, some of which are found to be associated with ASD [24–27]. However, although a majority of studies found elevated BDNF levels in the serum of ASD patients and abnormal mTOR activation in ASD patients [28–31], implying that aberrant activation of the BDNF/TrkB signaling may result in disease phenotypes, some other studies showed that activating the BDNF/TrkB signaling actually attenuated disease-like phenotypes in several ASD animal models [32–35].

Herein, we identified a novel function of MDGA2 in competing with BDNF for binding to TrkB and thus constraining the BDNF/TrkB signaling. We found that *Mdga2* heterozygous knockout (KO) (*Mdga2*^ +/−^) mice exhibited aberrant TrkB activation, increased α-amino-3-hydroxy-5-methyl-4-isoxazole propionic acid (AMPA) receptor (AMPAR)-mediated excitatory synapse transmission, and ASD-like behaviors including social deficits, repetitive behavior, and impaired cognition. Importantly, we showed that suppression of the TrkB activity by using its antagonist ANA-12 and using a peptide derived from MDGA2, as well as inhibition of the AMPAR activity attenuated social deficits in *Mdga2*-deficient mice. Our findings not only identify a previously undescribed mechanism underlying MDGA2 deficiency-induced ASD phenotypes, but also suggest that targeting MDGA2-mediated BDNF/TrkB signaling has therapeutic potential for ASD.

## Results

### *Mdga2* ^*+/* −^ mice exhibit autism-like behaviors and increased excitatory synapse density

MDGA2 was abundantly expressed in mouse medial prefrontal cortex (mPFC) and hippocampus (S1A Fig). Among different cell types, MDGA2 was highly expressed in neurons compared to astrocytes and microglia (S1B Fig). The expression of MDGA2 was gradually increased from embryonic day (E) 14 to postnatal day (P) 14 and then moderately decreased at P 30 and P 60, implying that MDGA2 may be involved in brain development (S1C Fig). Moreover, we found that MDGA2 markedly co-localized with the excitatory postsynaptic marker PSD-95 in both cultured primary neurons and in mouse hippocampus (S1D and S1I Fig).

To further investigate the role of MDGA2, we acquired *Mdga2* KO mice lacking the entire exon2 of the *Mdga2* gene (S1E Fig). We confirmed that *Mdga2* gene expression and MDGA2 protein levels in the brain were completely absent in *Mdga2* homozygous KO (*Mdga2*^−*/*−^) mice and approximately half in *Mdga2* heterozygous KO (*Mdga2*^* +/*−^) mice when compared to wild-type (WT) littermate controls (S1F–S1I Fig).

**Fig 1:**
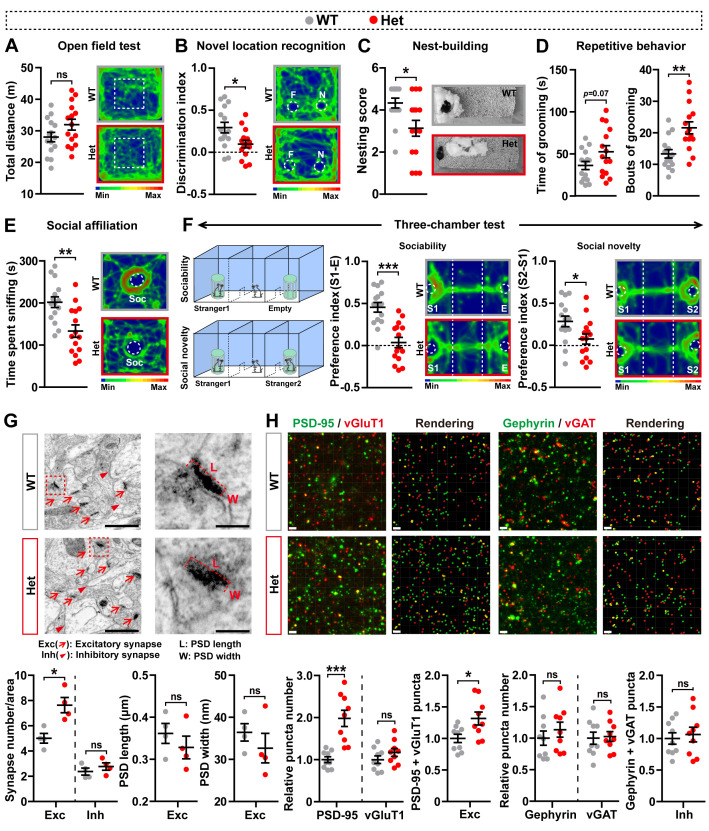
Mdga2 ^+/^ ^−^ mice exhibit autism-like behaviors and elevated excitatory synapse density. (**A**–**F**) 1.5–2 month-old *Mdga2* ^+/−^ (Het) mice and their WT littermates were analyzed for their total travel distance and time spent in the center in the open field test (A), their discrimination index in the novel location recognition test (B), their nesting score in the nest-building test (C), their time spent self-grooming and bouts of self-grooming (D), their time spent sniffing a reference mouse in the social affiliation test (E), and their sociability preference for sniffing a stranger mouse (S1) over an empty cylinder (F, middle panels) and social novelty preference for sniffing a new stranger mouse (S2) over the familiar S1 mouse (F, right panels) in the three-chamber test. WT: *n* = 15; Het: *n* = 15. (**G**) Excitatory (Exc, red arrow) and inhibitory (Inh, red arrowheads) synapses are indicated in the representative electron micrographs of the hippocampal CA1 stratum radiatum from Het mice and their WT littermates. The numbers of excitatory and inhibitory synapses were quantified for comparison. The PSD length (L) and PSD width (W) of excitatory synapses in enlargements were also quantified for comparison Scale bars: 1 μm or 200 nm for enlargements. *n* = 4 mice per group, with an average of 25 synapses from each mouse. (**H**) Confocal images and three-dimensional rendering of confocal stacks of the CA1 stratum radiatum from Het mice and their WT littermates showing excitatory synapses identified by co-localized puncta between vGluT1 (red) and PSD-95 (green), as well as inhibitory synapses identified by co-localized puncta between vGAT (red) and Gephyrin (green). Scale bars: 2 μm. The numbers of PSD-95, vGluT1, Gephyrin, and vGAT puncta, as well as the numbers of excitatory and inhibitory synapses, were quantified and normalized to those in WT (set to one arbitrary unit) for comparison. *n* = 9 slices from 4 mice per group. Data represent mean ± SEM. *P*-values were determined by two-tailed unpaired Student *t*-test. *p < 0.05, **p < 0.01, ***p < 0.001, ns: not significant. The data underlying this figure can be found in S1 Data, specifically in the sheet labeled “Figure 1”.

Since *Mdga2*^−*/*−^ mice die at about postnatal day 10 (P 10), we used *Mdga2*^* +/*−^ mice for behavioral experiments. Although *Mdga2*^* +/*−^ mice showed normal locomotor activity in the open field test (Fig 1A), they exhibited recognition memory deficit in the novel location recognition (NLR) test (Fig 1B). Moreover, we found that in consistent with a previous study [18], *Mdga2*^* +/*−^ mice displayed autism-like behaviors including impaired nest building ability (Fig 1C), increased repetitive self-grooming behavior (Fig 1D), and deficits in social affiliation (Fig 1E), sociability (Fig 1F), and social novelty (Fig 1F).

Next, we examined the morphologies of synapses in the hippocampus. Electron microscopy analysis showed increased numbers of excitatory synapses but not inhibitory synapses in the hippocampal CA1 stratum radiatum of *Mdga2*^* +/*−^ mice when compared to controls, though the length and width of postsynaptic density (PSD) of excitatory synapses appeared unaltered (Fig 1G). Co-immunostaining of presynaptic and postsynaptic markers revealed that *Mdga2*^* +/*−^ mice had increased vGluT1^ +^/PSD-95^ +^ puncta but not vGAT^ +^/Gephyrin^ +^ puncta when compared to controls (Fig 1H). These results suggest that MDGA2 deficiency leads to an aberrant increase in excitatory synapses; and this may cause abnormal behaviors in *Mdga2*^* +/*−^ mice.

### Hyperactivation of the BDNF/TrkB signaling pathway in *Mdga2*-deficient mice

**Fig 2:**
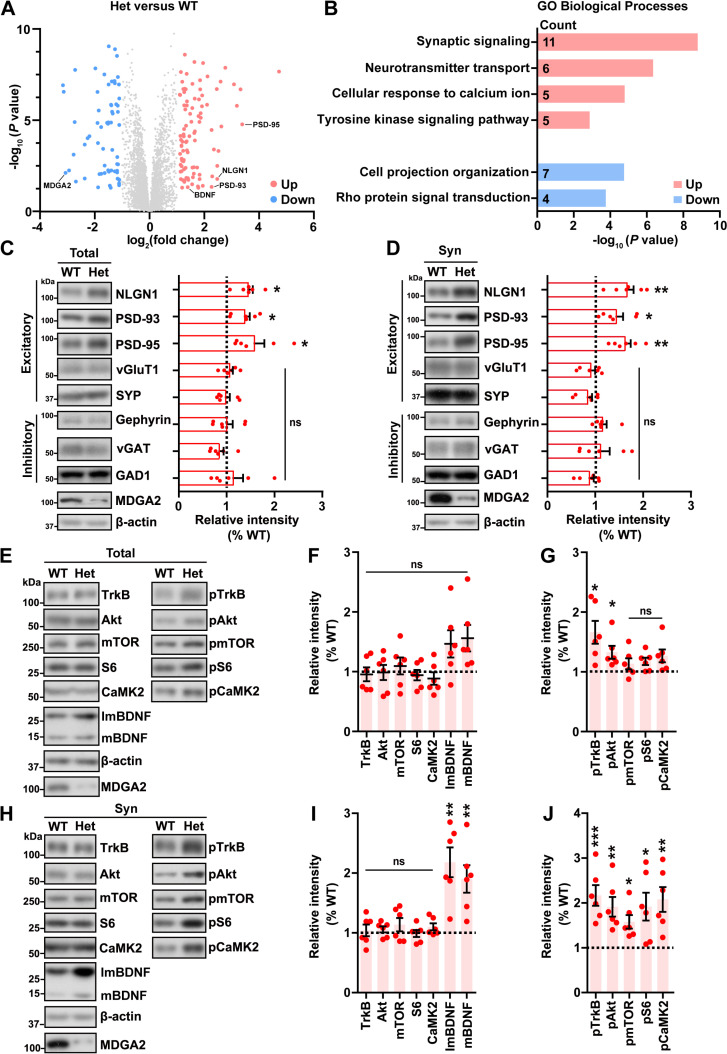
*Mdga2* haploinsufficiency promotes the TrkB-Akt signaling pathway. (**A**) Proteomic analysis identified up-regulated (red) and down-regulated (blue) proteins in the hippocampus of 1.5–2-month-old *Mdga2*^ +/−^ (Het) mice vs. their wild-type (WT) littermates (*n* = 4 per group, fold change >1.1, *p* < 0.05). (**B**) GO analysis of biological processes of altered proteins. (**C**, **D**) Equal protein amounts of total lysates (C) and synaptosome (Syn) fractions (D) derived from the hippocampus of 1.5–2-month-old Het mice and their WT littermates were subjected to immunoblotting for the proteins indicated. Protein levels were quantified by densitometry, normalized to β-actin levels, and compared to those of WT (set to one arbitrary units; indicated by dashed lines). (**E**–**J**) Equal protein amounts of total lysates (E) and Syn fractions (H) derived from the hippocampus of 1.5–2-month-old Het mice and their WT littermates were subjected to immunoblotting for the proteins indicated. The levels of proteins indicated were quantified by densitometry, normalized to those of β-actin, and compared to those of WT littermates (set to one arbitrary units; indicated by dashed lines) (F, I). The levels of phosphorylated (p) proteins were normalized to respective total protein levels and then compared to those of WT littermates (set to one arbitrary units; indicated by dashed lines) (G, J). Data represent mean ± SEM. *n* = 6 mice per group in (C–J). *P*-values were determined by two-tailed unpaired Student *t*-tes*t*. *p < 0.05, **p < 0.01, ***p < 0.001, ns: not significant. The data underlying this figure can be found in S1 Data, specifically in the sheet labeled “Figure 2”.

To determine the molecular mechanism underlying MDGA2 deficiency-induced pathology, we examined protein expression profiles of 1.5- to 2-month-old WT and *Mdga2*^* +/*−^ mouse hippocampus by Data-independent icquisition (DIA) quantification proteomics. Ninety-seven down-regulated and 99 up-regulated differentially expressed proteins (DEPs) were identified (fold change > 1.1, *p* < 0.05, S1–S3 Tables) and compared by scatter plots (Fig 2A). A reduction of MDGA2 protein levels and an increase of NLGN1, PSD-93, and PSD-95 protein levels were identified. Gene ontology (GO) analysis of biological processes revealed an enrichment of proteins related to “synaptic signaling” and “tyrosine kinase signaling pathway” in the upregulated DEPs (Fig 2B). Indeed, immunoblotting analysis showed that levels of total (Fig 2C) and synaptosomal (Fig 2D) NLGN-1, PSD-95, and PSD-93, all of which are enriched in the excitatory PSD, were significantly increased in *Mdga2*^* +/*−^ mice when compared to controls. While levels of presynaptic proteins (vGluT1, SYP, vGAT, and GAD1) and the inhibitory postsynaptic protein Gephyrin were not altered. Such a discrepancy may be attributed to a major expression of MDGA2 in excitatory neurons, so that its deficiency has a dominant effect on protein production in excitatory neurons. Increased PSD-95 but not vGluT1 levels in *Mdga2*^* +/*−^ mice were also confirmed by immunofluorescence staining (Fig 1H), implying that *Mdga2*^* +/*−^ mice have more immature excitatory synapses than controls.

Interestingly, the levels of BDNF, one important factor triggering the TrkB tyrosine kinase signaling pathway involved in ASD, were found to be increased in *Mdga2*^* +/*−^ mice through the proteomic profiling (Fig 2A). Immunoblotting assays also showed that although immature BDNF (ImBDNF) and mature BDNF (mBDNF) protein levels in the total hippocampal tissues of *Mdga2*^ +/−^ mice were not significantly different from those of WT controls (Fig 2E and 2F), their levels in synaptosomal fractions of *Mdga2*^ +/−^ hippocampus were significantly increased (Fig 2H and 2I). Therefore, we further studied several crucial proteins downstream the BDNF/TrKB signaling pathway. Levels of TrkB, Akt, mTOR, S6, and CaMK2 in total and synaptosomal fractions from the hippocampus of *Mdga2*^ +/−^ mice were unaltered. However, phosphorylated forms of all these proteins (pTrkB, pAkt, pmTOR, pS6, and pCaMK2) were significantly increased in synaptosomal fractions of *Mdga2*^ +/−^ mouse hippocampus when compared to those of controls; and levels of pTrkB and pAkt were also significantly increased in total fractions of *Mdga2*^ +/−^ mouse hippocampus when compared to those of controls (Fig 2E–2J). In cultured primary neurons, we also found that MDGA2 deficiency not only led to increased levels of pTrkB, pAkt, pmTOR, NLGN1, PSD-93, and PSD-95, but also promoted overall protein production (S2A Fig). This is consistent with the notion that the TrkB-Akt-mTOR pathway activates protein translation in synapses [27,36–39].

### MDGA2 competes with BDNF for TrkB binding and suppresses TrkB activity

**Fig 3:**
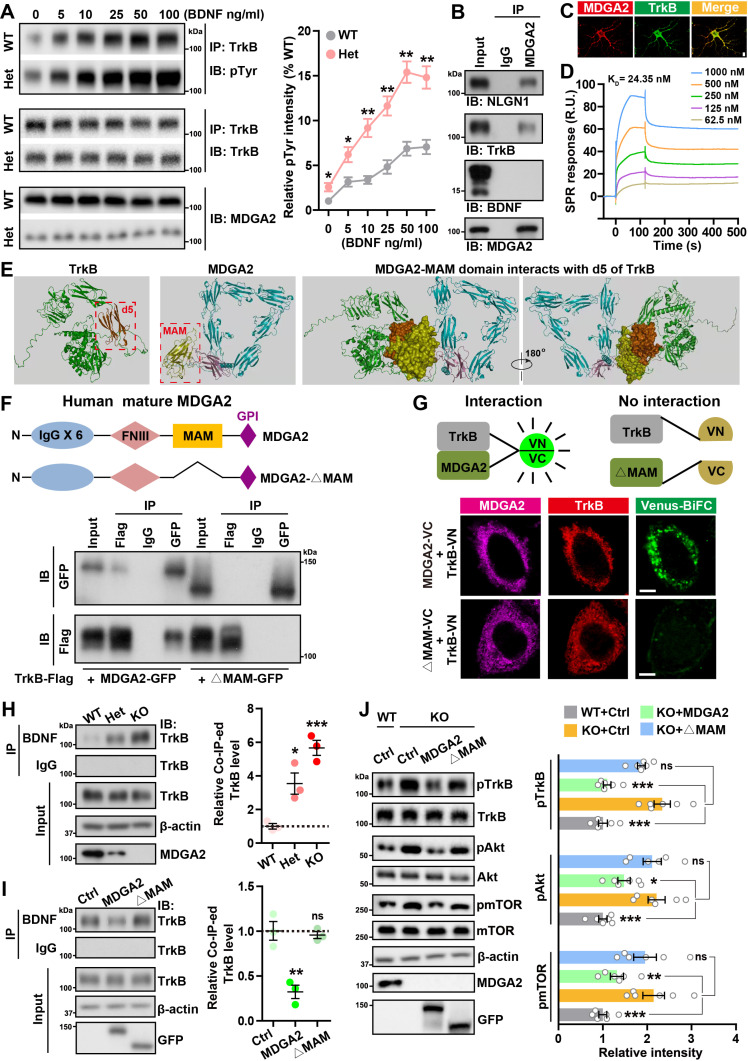
MDGA2 interacts with TrkB and suppresses TrkB activity. (**A**) Primary hippocampal neurons derived from *Mdga2*^ +/−^ (Het) mice and their wild-type (WT) littermates at DIV 7 were treated with different doses of BDNF for 30 min. Equal amounts of protein lysates were subjected to immunoprecipitation (IP) with an anti-TrkB antibody, followed by immunoblotting with an anti-phosphotyrosine (pTyr) antibody or the anti-TrkB antibody. The pTyr levels were quantified by densitometry, normalized to total TrkB levels, and then compared to those of WT controls. *n* = 3 per group. (**B**) WT mouse brain lysates were subjected to IP with an anti-MDGA2 antibody and a control IgG, and immunoblotted (IB) for the components indicated. mBDNF, mature BDNF; ImBDNF, immature BDNF. (**C**) Hippocampal neurons from WT mice at DIV 7 were immunostained with antibodies against MDGA2 (red) and TrkB (green), and then counter-stained with DAPI (blue). Images were acquired by a confocal microscope. Scale bar: 10 μm. (**D**) Surface plasmon resonance (SPR) analysis of the binding between recombinant MDGA2 protein and recombinant TrkB protein. TrkB was immobilized on a Biacore CM5 chip and tested for its binding with MDGA2 at the concentrations indicated. (**E**) Computer simulation of the binding mode of TrkB and MDGA2. The predicted binding domain of TrkB (the d5 domain) is highlighted by a red square and labeled in brown. The predicted binding domain of MDGA2 (the MAM domain) is highlighted by a red square and labeled in yellow. (**F**) HEK293T cells were co-transfected with TrkB-Flag and GFP-tagged full-length MDGA2 (MDGA2-GFP) or an MDGA2 deletion fragment lacking the entire MAM domain (ΔMAM-GFP). Cell lysates were subjected to IP with control IgG and antibodies against Flag or GFP, and immunoblotted for the components indicated. A scheme of full-length MDGA2 shows its six IgG domains, FNIII domain, MAM domain, and C-terminal GPI anchor. The scheme of MDGA2-ΔMAM is also shown. (**G**) HeLa cells were co-transfected with TrkB that is tagged with the N-terminal Venus fragment (TrkB-VN) and full-length MDGA2 or MDGA2-ΔMAM that are tagged with the C-terminal Venus fragment (MDGA2-VC or ΔMAM-VC) for 24 h. After immunostaining with antibodies against MDGA2/MDGA2-ΔMAM (pink) and TrkB (red), cells were observed by confocal microscopy. Venus signal (green) indicates the interaction between MDGA2 and TrkB. Schematic diagrams depicting the BiFC assay are also shown. Scale bars: 10 μm. (**H**, **I**) Equal amounts of protein lysates from primary neurons derived from Het, KO, and their WT littermate mice (H), and from WT primary neurons transfected with GFP control, MDGA2-GFP or ΔMAM-GFP (I) were subjected to IP with IgG and an anti-BDNF antibody, and then immunoblotted for the components indicated. Immunoprecipitated TrkB levels were quantified by densitometry, normalized to input levels, and compared to respective controls (set to one arbitrary units). *n* = 3 per group. (**J**) Neurons derived from KO mice were transfected with GFP control (Ctrl), MDGA2-GFP, or MDGA2-ΔMAM-GFP, and neurons derived from their WT littermates were transfected with GFP control (Ctrl). Equal amounts of protein lysates were subjected to immunoblotting for the proteins indicated. Levels of phosphorylated proteins were quantified by densitometry, normalized to respective total protein levels and compared to controls (set to one arbitrary units). *n* = 6 per group. Data represent mean ± SEM. *P*-values were determined by two-tailed unpaired Student *t*-test in (A) and one-way ANOVA with Tukey’s multiple comparisons test in (H, I, J). *p < 0.05, **p < 0.01, ***p < 0.001, ns: not significant. The data underlying this figure can be found in S1 Data, specifically in the sheet labeled “Figure 3”.

BDNF binding triggers TrkB phosphorylation and hence its activation. We examined the BDNF/TrkB signaling in *Mdga2*-deficient primary neurons. Phosphorylation of TrkB induced by BDNF showed a dose-dependent manner and was much stronger in *Mdga2*^* +/*−^ and *Mdga2*^−*/*−^ neurons than in WT neurons (Figs 3A and S3A). When MDGA2 was overexpressed in cells and MDGA2-interacting proteins were pulled down and subjected to mass spectrometry analysis, TrkB peptide was identified among pulled-down proteins (S3B Fig). Moreover, in WT mouse brain tissues, we found that an anti-MDGA2 antibody immunoprecipitated TrkB and NLGN1 but not mBDNF or ImBDNF (Fig 3B), and an anti-TrkB antibody specifically immunoprecipitated MDGA2 but not MDGA1 (S3C Fig). Co-immunostaining experiments also showed that endogenous MDGA2 co-localized with endogenous TrkB (Figs 3C and S3D). Furthermore, surface plasmon resonance (SPR) analysis demonstrated that recombinant MDGA2 protein had a dose-dependent binding affinity to recombinant TrkB protein in vitro, with a *K*_D_ value of 24.35 nM (Fig 3D).

MDGA2 is attached to the postsynaptic membrane via a C-terminal GPI anchor and its extracellular domain consists of six immunoglobulin-like domains (IgG × 6), a fibronectin type III-like (FNIII) domain, and a MAM domain (Fig 3E) [12,40,41]. We used the structures of MDGA2 and TrkB to simulate their interaction and predicted a potential interaction between the MDGA2 MAM domain and the TrkB IgG2/d5 domain (Fig 3E). Interestingly, MDGA2 mutations with truncations covering the MAM domain have been previously reported in individuals with ASD [8]. Therefore, we generated a truncated form of MDGA2 lacking its MAM domain (MDGA2-ΔMAM, Fig 3F). We found that although loss of MAM had no effect on its cell membrane localization (S3E Fig), it abolished the MDGA2-TrkB interaction (Fig 3F). Consistently, BiFC assays showed that TrkB tagged with the N-terminal venus fragment could interact with full-length MDGA2 but not MDGA2-ΔMAM, both of which are tagged with the C-terminal venus fragment, to generate venus fluorescence (Fig 3G).

Given that TrkB signaling is dependent on BDNF stimulation, we speculate that MDGA2 competes with BDNF for TrkB interaction. Computer simulation predicted that BDNF interacted with the TrkB IgG2/d5 domain (S3F Fig); and this interaction has previously been experimentally confirmed [42]. Since the TrkB IgG2/d5 domain was also predicted to interact with the MDGA2 MAM domain (Fig 3E), we generated a TrkB truncated form lacking the IgG2/d5 domain (TrkB-Δd5) (S3G Fig). Co-immunoprecipitation study revealed that loss of the IgG2/d5 domain indeed abolished TrkB interaction with MDGA2 (S3H Fig). Furthermore, we found that BDNF interacted with much more TrkB in *Mdga2*-deficient neurons than in WT controls (Figs 3H and S3I). Moreover, overexpression of full-length MDGA2 but not MDGA2-ΔMAM attenuated the interaction between BDNF and TrkB (Fig 3I). Consistently, BDNF-induced TrkB signaling activation in WT primary neurons was attenuated upon transfection with full-length MDGA2 but not with MDGA2-ΔMAM (S3J Fig). Importantly, overactivation of the TrkB signaling in *Mdga2*^−/−^ neurons, as indicated by the elevated levels of pTrkB, pAkt, and pmTOR, could be reversed upon the expression of full-length MDGA2 but not MDGA2-ΔMAM (Fig 3J). Together, these results indicate that MDGA2 interacts with TrkB through the MDGA2 MAM domain to block TrkB-BDNF binding and constrain TrkB activation. Additionally, we found that increased NLGN1 levels in MDGA2-deficient neurons were reduced upon ANA-12 treatment to inhibit the TrkB signaling pathway (S2A and S2B Fig), whereas knockdown of NLGN1 in MDGA2-deficient neurons had no effect on the TrkB signaling pathway (S3K Fig), suggesting that the regulation of TrkB by MDGA2 is upstream of the NLGN1 pathway.

### Blocking the BDNF-TrkB interaction by an MDGA2-derived peptide attenuates social deficits in *Mdga2* ^+/−^ mice

**Fig 4:**
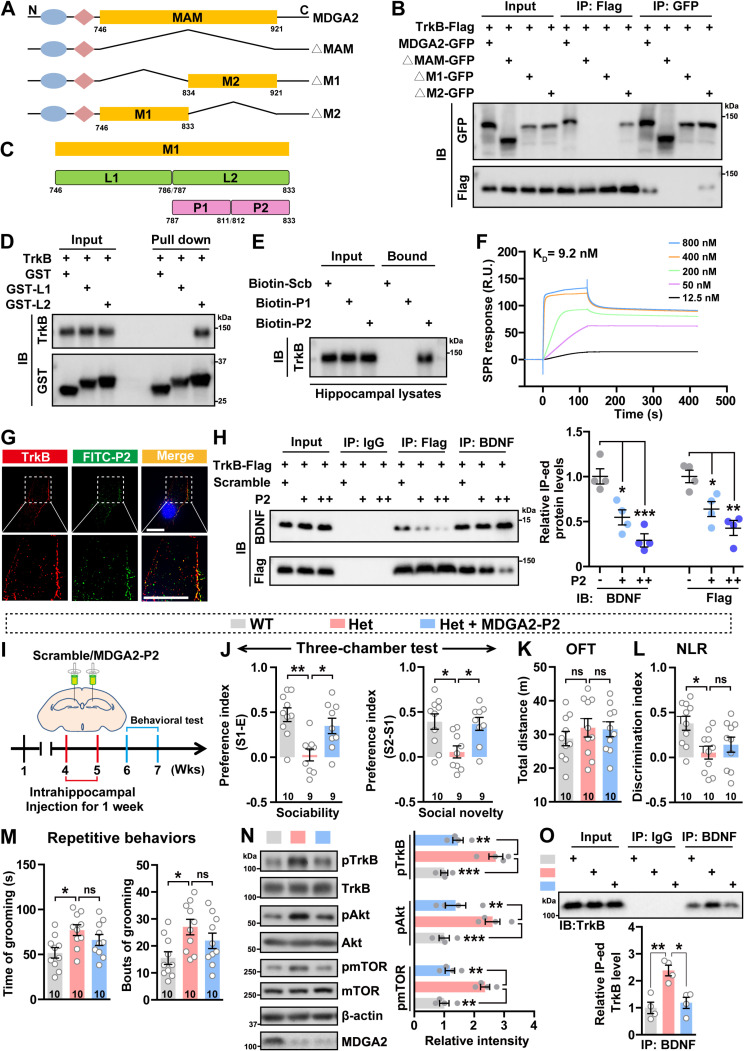
Blocking BDNF-TrkB interaction in the hippocampus rescues social deficits in *Mdga2*^+/−^ mice. (**A**) Schematic depictions of human MDGA2 and its truncated constructs. ΔMAM, lacking the entire memprin/A5/mu (MAM) domain. ΔM1: lacking the N-terminal region (aa 746–833) of MAM domain. ΔM2: lacking the C-terminal region (aa 834–921) of MAM domain. N: N-terminus. C: C-terminus. (**B**) HEK293T cells were co-transfected with tyrosine kinase B (TrkB)-Flag and GFP-tagged full-length MDGA2, MDGA2-ΔMAM, MDGA2-ΔM1, or MDGA2-ΔM2. Cell lysates were subjected to immunoprecipitation (IP) with antibodies against Flag or GFP, and immunoblotted (IB) for the components indicated. (**C**) Schemes of fragments derived from the MDGA2 MAM-M1 domain, including M1 (aa 746–833), L1 (aa 746–786), L2 (aa 787–833), P1 (aa 787–811), and P2 (aa 812–833). (**D**) GST-fused L1 and L2 were incubated with human TrkB protein and pulled down with Glutathione Sepharose 4B. Samples were then immunoblotted for indicated proteins. (**E**) 1 μg P1, P2, and scrambled (Scb) peptides conjugated with biotin were bilaterally injected into the hippocampal regions of wild-type (WT) mice. Mice were sacrificed 24 h later. Equal protein amounts of hippocampal lysates were precipitated with avidin beads and immunoblotted with an antibody against TrkB. (**F**) Surface plasmon resonance (SPR) analysis of the binding between MDGA2-P2 and TrkB. TrkB was immobilized on a Biacore CM5 chip and tested for its binding with P2 at the indicated concentrations. (**G**) SH-SY5Y cells were incubated with FITC-labeled P2 peptide (100 nM) for 16 h, immunostained with an antibody against TrkB (red), and then counter-stained with DAPI (blue). Images were acquired by High Sensitivity Structured Illumination Microscope (HiS-SIM). Scale bar: 10 μm. (**H**) *Mdga2*^ +/−^ neurons were co-transfected with TrkB-Flag for 8 h, and then incubated with increasing quantities (+: 0.1 μm; ++: 1 μm) of P2 peptide for 16 h. Cell lysates were subjected to IP with control IgG or antibodies against Flag or BDNF, and immunoblotted for the components indicated. Immunoprecipitated protein levels were quantified by densitometry, normalized to respective inputs, and compared to those without P2 treatment (set to one arbitrary units). *n* = 4 experimental replicates. (**I**) Schematic depiction of the experimental strategy. 4-week-old *Mdga2*^ +/−^ (Het) mice and their WT littermates were administered with 1 μg scrambled or MDGA2-P2 peptide via bilateral intrahippocampal injection once per day for 1 week. After another week, mice were studied for their behaviors. (**J**–**M**) Treated mice were analyzed for their sociability and social novelty preferences in the three-chamber test (J), their total travel distance in the open field test (K), their discrimination index in the novel location recognition test (L), and their time spent self-grooming and bouts of self-grooming (M). WT, *n* = 10; Het, *n* = 9 (for I, J) and *n* = 10 (for K–M); MDGA2-P2-treated Het, *n* = 9 (for I, J) and *n* = 10 (for K–M). (**N**) Equal amounts of protein lysates from the hippocampus of treated mice were immunoblotted for the proteins indicated. Levels of phosphorylated proteins were quantified by densitometry, normalized to respective total protein levels, and compared to WT controls (set to one arbitrary units). *n* = 4 mice per group. (**O**) Equal amounts of protein lysates from the hippocampus of treated mice were subjected to immunoprecipitation (IP) with IgG or an anti-BDNF antibody, and then immunoblotted for TrkB. Immunoprecipitated TrkB levels were quantified by densitometry, normalized to input levels, and compared to controls (set to one arbitrary unit). *n* = 4 per group. Data represent mean ± SEM. *P*-values were determined by one-way ANOVA with Tukey’s multiple comparisons test. *p < 0.05, **p < 0.01, ***p < 0.001, ns: not significant. The data underlying this figure can be found in S1 Data, specifically in the sheet labeled “Figure 4”.

The MDGA2 MAM domain contains 176 amino acids. To further determine the MAM region that interacts with TrkB, we performed domain mapping assays by truncating the N-terminal region (aa 746–833, M1) and the C-terminal region (aa 834–921, M2) of the MAM domain, respectively (Fig 4A). Co-immunoprecipitation assays showed that truncation of M1 but not M2 abolished the interaction between MDGA2 and TrkB (Fig 4B). Next, we divided M1 into two segments, L1 (aa 746–786) and L2 (aa 787–833), and generated GST-tagged L1 and L2 fragments (Fig 4C and 4D). In vitro pull-down assays revealed that only GST-tagged L2 and but not GST-tagged L1 interacted with TrkB (Fig 4D). We further divided L2 into two segments, P1 (aa 787–811) and P2 (aa 812–833), and synthesized biotin-conjugated P1 and P2 peptides (Fig 4C and 4E). Subsequently, the two peptides were injected into mouse hippocampus and biotin pull-down assays were carried out. The results showed that P2, but not P1, bound to TrkB in vivo (Fig 4E). Moreover, we conducted SPR analysis to characterize the direct interaction between MDGA2-P2 and TrkB, revealing that P2 interacted with TrkB with a *K*_D_ value of 9.2 nM (Fig 4F). In addition, fluorescein isothiocyanate (FITC)-conjugated P2 had marked co-localization with TrkB in SH-SY5Y cells (Fig 4G). Furthermore, we performed competitive binding assays and found that P2 treatment attenuated TrkB interaction with BDNF in a dose-dependent manner (Fig 4H). Together, these results indicate that the P2 region (aa 812–833) is an essential domain for MDGA2 to bind to TrkB.

Given that MDGA2-P2 peptide can block TrkB-BDNF interaction, we bilaterally injected MDGA2-P2 or a scrambled peptide into the hippocampal regions of *Mdga2*^* +/*−^ mice once per day for 1 week. Treated mice were subjected to behavioral and biochemical analysis after another week (Fig 4I). In the three-chamber test, MDGA2-P2 peptide treatment also rescued sociability and social novelty deficits in *Mdga2*^* +/*−^ mice (Fig 4J). However, MDGA2-P2 peptide treatment did not affect locomotor activity (Fig 4K), rescue impaired cognitive function (Fig 4L) or reduce repetitive self-grooming behavior (Fig 4M) in *Mdga2*^ +/−^ mice. As expected, MDGA2-P2 peptide treatment effectively reversed over-activation of the TrkB signaling (Fig 4N) and reduced the interaction between BDNF and TrkB (Fig 4O) in *Mdga2*^* +/*−^ mice. These findings collectively suggest that blockage of the interaction between BDNF and TrkB has therapeutic potential for ASD caused by MDGA2 deficiency.

### Pharmacological inhibition of TrkB attenuates social deficits and AMPAR dysfunction in *Mdga2* ^+/−^ mice

ANA-12 is a selective and blood–brain barrier-permeable TrkB antagonist. We also explored whether ANA-12 can rescue deficits in *Mdga2*^ +/−^ mice. In primary neurons derived from *Mdga2*^ +/−^ and *Mdga2*^−/−^ mice, ANA-12 effectively reversed increased levels of pTrkB, pAkt, pmTOR, NLGN1, PSD-95, and PSD-93 (S2A and S2B Fig). We also tested the efficacy of ANA-12 in vivo at different time points and found that ANA-12 inhibited the TrkB signaling mostly at 16–24 h after treatment in both WT and *Mdga2*^−/−^ mice (S4A Fig).

To determine whether a short-term treatment can attenuate disease-related phenotypes and how long the benefit of such a short-term treatment can last, we applied acute ANA-12 administration to mice. During the acute administration, 4-week-old mice were subjected to intraperitoneal (i.p.) delivery of ANA-12 at 0.5 mg/kg body weight, once daily for three consecutive days, and then behavioral tests at 1 day (acute Test 1) or 1 week (acute Test 2) after treatments (S4B Fig). We found that acute ANA-12 treatment did not affect locomotor activity of mice in the open field (S4C Fig), nor did it attenuate impaired cognitive function (Fig 4D) and repetitive behavior (S4E Fig) in *Mdga2*^ +/−^ mice. In the social affiliation test, acute ANA-12-treated *Mdga2*^ +/−^ mice showed a significantly higher social preference compared to controls in acute Test 1 but not in acute Test 2 (S4F Fig). Similarly, in the three-chamber test, acute ANA-12-treated *Mdga2*^ +/−^ mice showed a significantly higher sociability preference when compared to controls in acute Test 1 but not in acute Test 2 (S4G Fig). These results suggest that acute ANA-12 treatment can attenuate social deficits in *Mdga2*^ +/−^ mice, but the benefit is temporary.

**Fig 5:**
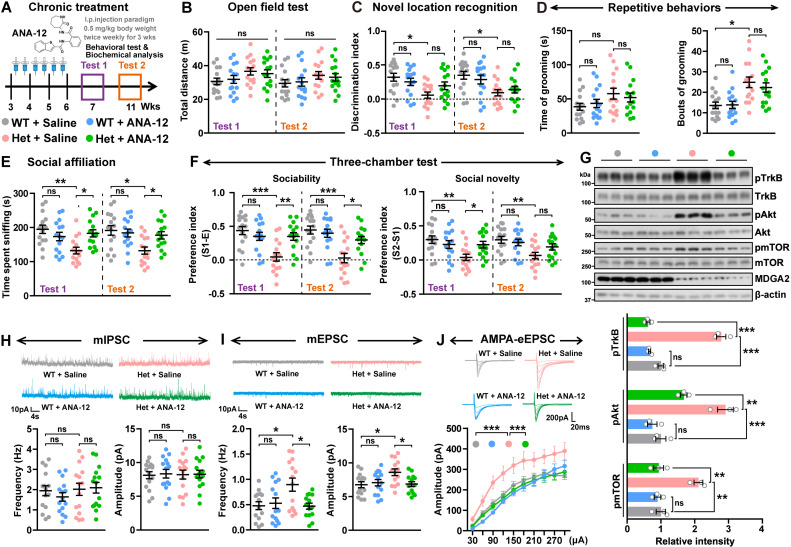
Chronic ANA-12 treatment rescues social deficits in *Mdga2* ^+/^− mice. (**A**) ANA-12 chronic treatment scheme. ANA-12 was intraperitoneally injected into 3-week-old *Mdga2*^ +/−^ (Het) mice and their WT littermates at 0.5 mg/kg body weight, twice per week for 3 weeks. Behavioral tests were carried out when mice were 7- (Test 1) and 11- (Test 2) weeks old. (**B**–**F**) Treated WT and *Mdga2*^ +/−^ (Het) mice were studied for their total travel distance in the open field test (B), their discrimination index in the novel location recognition test (C), their time spent self-grooming and bouts of self-grooming (D, only chronic test 1 results are shown), their sniffing time in the social affiliation test (E), and their sociability and social novelty preferences in the three-chamber test (F). *n* = 15 mice per group. (**G**) Equal amounts of protein lysates from treated mice were immunoblotted for the proteins indicated. Levels of phosphorylated proteins were quantified by densitometry, normalized to respective total protein levels, and compared to WT littermate controls (set to one arbitrary units). *n* = 3 mice per group. (**H**, **I**) The miniature inhibitory postsynaptic currents (mIPSCs) (H) and miniature excitatory postsynaptic currents (mEPSCs) (I) of hippocampal neurons in treated mice were recorded and their frequencies and amplitudes were quantified for comparison. *n* = 15 cells from 3 mice per group for mIPSCs; *n* = 14 cells from 3 mice per group for mEPSCs. (**J**) Representative traces of evoked AMPAR-mediated EPSCs and quantitative analysis of evoked AMPAR-mediated EPSC amplitude with increasing stimulus intensity. *n* = 20–21 cells from 3 mice per group. Data represent mean ± SEM. *P*-values were determined by two-way ANOVA with Tukey’s multiple comparisons test in (B–I) and repeated-measures ANOVA followed by Bonferroni’s post hoc analysis in (J). *p < 0.05, **p < 0.01, ***p < 0.001, ns: not significant. For Test 1: Interaction *F*(1,56) = 0.2753, *p = *0.6019, and for Test 2: Interaction *F*(1,56) = 0.2774, *p = *0.6005 in B. For Test 1: Interaction *F*(1,56) = 0.6210, *p = *0.4340, and for Test 2: Interaction *F*(1,56) = 0.7506, *p = *0.3900 in C. For Test 1: Interaction *F*(1,56) = 0.1344, *p = *0.7153, and for Test 2: Interaction *F*(1,56) = 0.2389, *p = *0.6269 in D. For Test 1: Interaction *F*(1,56) = 10.07, *p = *0.0024, and for Test 2: Interaction *F*(1,56) = 4.906, *p = *0.0309 in E. For Test 1: Interaction *F*(1,56) = 13.52, *p = *0.0005, and for Test 2: Interaction *F*(1,56) = 7.339, *p = *0.0089 in F left. For Test 1: Interaction *F*(1,56) = 3.144, *p = *0.0817, and for Test 2: Interaction *F*(1,56) = 7.339, *p = *0.0089 in F right. Interaction *F*(1,8) = 17.59, *p = *0.0030 for pmTOR*,* Interaction *F*(1,8) = 9.145, *p = *0.0165 for pAkt, and Interaction *F*(1,8) = 108.6, *p < *0.0001 for pTrkB in G. Interaction *F*(1,56) = 0.5436, *p = *0.4640 in H left. Interaction *F*(1,56) = 0.0127, *p = *0.9106 in H right. Interaction *F*(1,52) = 6.023, *p = *0.0175 in I left. Interaction *F*(1,52) = 5.282, *p = *0.0256 in I right. The data underlying this figure can be found in S1 Data, specifically in the sheet labeled “Figure 5”.

We next studied whether chronic administration of ANA-12 has relatively long benefit. We treated 3-week-old mice with i.p. delivery of ANA-12 at 0.5 mg/kg body weight, twice per week for three consecutive weeks, and then carried out behavioral tests 1 week (chronic Test 1) and 5 weeks later (chronic Test 2, Fig 5A). Chronic ANA-12 treatment did not affect the locomotor activity (Fig 5B) or recognition memory (Fig 5C) of *Mdga2*^ +/−^ mice during both chronic Test 1 and chronic Test 2. However, in the social affiliation test, chronic ANA-12 treatment significantly improved impaired social affiliation in *Mdga2*^ +/−^ mice in both chronic Test 1 and chronic Test 2 (Fig 5E). In the three-chamber test, chronic ANA-12 treatment also significantly improved impaired sociability and social novelty in *Mdga2*^ +/−^ mice in chronic Test 1, and significantly improved impaired sociability in *Mdga2*^ +/−^ mice in chronic Test 2 (Fig 5F). While chronic ANA-12 treatment had no rescuing effect on repetitive self-grooming behavior in *Mdga2*^ +/−^ mice (Fig 5D). Together, these results suggest that chronic ANA-12 treatment can attenuate social deficits in *Mdga2*^ +/−^ mice for a relatively long period. As expected, we found that altered phosphorylation of proteins crucially involved in the TrkB signaling was reversed by chronic ANA-12 treatment (Fig 5G).

We also performed whole-cell recordings of CA1 pyramidal neurons in hippocampal slices from treated mice. No differences in miniature inhibitory postsynaptic current (mIPSC) frequency or amplitude were observed between *Mdga2*^ +/−^ mice and WT littermate controls, nor did chronic ANA-12 treatment affect mIPSC frequency or amplitude in these mice (Fig 5H). However, both miniature excitatory postsynaptic current (mEPSC) frequency and amplitude were significantly higher in *Mdga2*^ +/−^ mice than in WT controls; and chronic ANA-12 treatment reversed the increased mEPSC amplitude and frequency in *Mdga2*^ +/−^ mice (Fig 5I). Further analysis of mIPSC and mEPSC kinetics, including the rise time and decay (S5A–S5D Fig) showed no change in *Mdga2*^ +/−^ mice, implicating that the MDGA2-TrkB axis has no effect on the composition of its downstream AMPA receptors. Moreover, evoked AMPAR-mediated EPSCs (Fig 5J) and the AMPAR/N-methyl-D-aspartate (NMDA) receptor (NMDAR) response ratios (S5F Fig) were markedly elevated in *Mdga2*^ +/−^ mice, which were effectively attenuated upon chronic ANA-12 treatment. However, no detectable differences were found in evoked NMDAR-mediated EPSCs between *Mdga2*^ +/−^ mice and WT controls (S5E Fig). In line with the electrophysiological results, cell surface distribution of the AMPAR subunit GluA1 but not the NMDAR subunit GluN1 was elevated in MDGA2-deficient neurons compared to WT neurons, and became comparable between these neurons upon ANA-12 treatment (S5G Fig). These results suggest that AMPAR hyperactivity induced by activated BDNF/TrkB signaling may represent a major cause of the ASD-like behaviors in *Mdga2*^ +/−^ mice. To confirm this, we treated *Mdga2*^ +/−^ mice with CP-465022 by i.p. injection at 1 mg/kg body weight (S5H Fig). CP-465022 is an AMPAR antagonist that holds potential for use in treating ASD-like behaviors [43]. We found that CP-465022 treatment rapidly promoted social affiliation and sociability (S5I and S5J Fig), without affecting social novelty, recognition memory, and repetitive self-grooming behavior in *Mdga2*^ +/−^ mice (S5K–S5M Fig). These results indicate that MDGA2-TrkB signaling-mediated AMPAR function is essential for social behaviors.

### MDGA2 deficiency in hippocampal excitatory neurons causes social impairment

**Fig 6:**
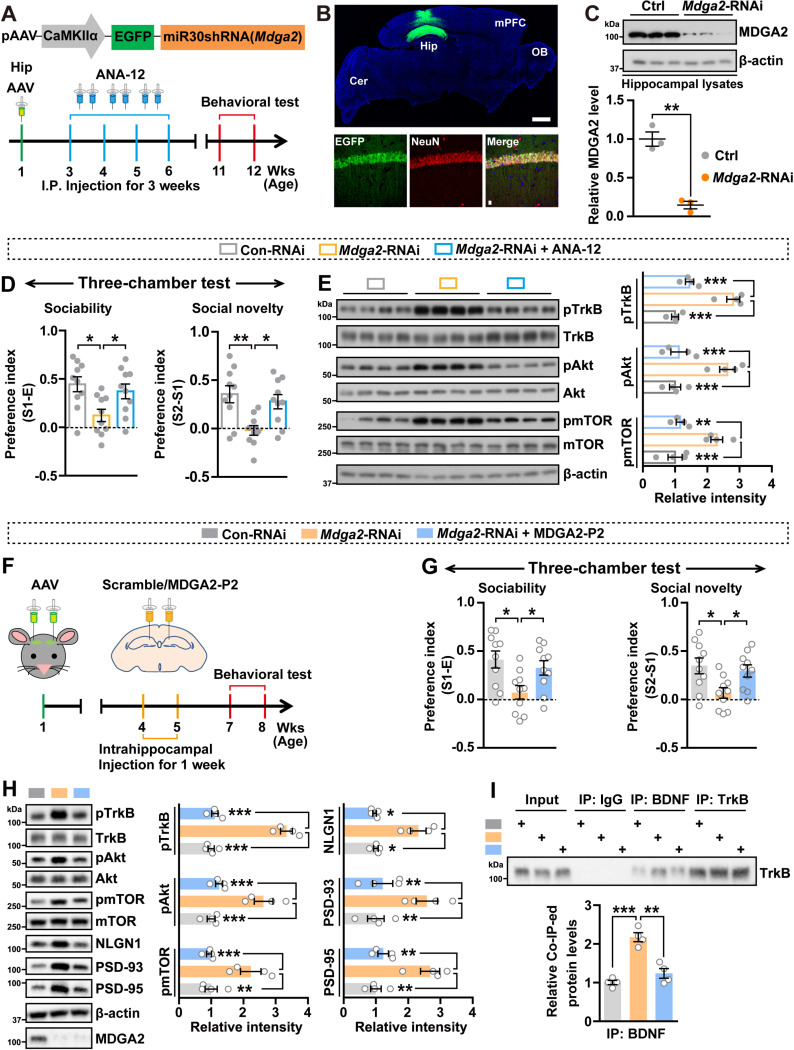
MDGA2 deficiency in hippocampal excitatory neurons is associated with social behaviors. (**A**) AAV injection and ANA-12 treatment scheme. AAVs containing a construct engineered to silence *Mdga2* and co-express EGFP, as well as their controls were bilaterally injected into the hippocampal regions of 1-week-old C57BL/6 WT mice. ANA-12 was intraperitoneally delivered when these mice were 3-weeks old at 0.5 mg/kg body weight, twice per week for three consecutive weeks. Behavioral tests were carried out when mice were 11-weeks old. (**B**) Mice subjected to hippocampal infection with AAVs were immunostained with an anti-NeuN antibody (red) three weeks later, followed by observation under a confocal microscope. Scale bar = 1 mm for the representative image. Scale bar = 10 μm for the enlarged images. Hip, hippocampus; OB, olfactory bulb; Cer, cerebellum. (**C**) MDGA2 protein levels from hippocampal extracts were analyzed by immunoblotting and quantiﬁed by densitometry with normalizing to β-actin levels. *n* = 3 mice per group. (**D**) Treated mice were analyzed for their sociability and social novelty preferences in the three-chamber test. *n* = 10 per group. (**E**) Equal amounts of protein lysates from treated mice were immunoblotted for the proteins indicated. Levels of phosphorylated proteins were quantified by densitometry, normalized to respective total protein levels, and compared to WT controls (set to one arbitrary units). *n* = 4 mice per group. (**F**) AAV injection and MDGA2-P2 peptide treatment scheme. AAVs were bilaterally injected into the hippocampal regions of 1-week-old C57BL/6 WT mice. After 3 weeks, mice were administered with scrambled or MDGA2-P2 peptide via bilateral intrahippocampal injections once per day for 1 week. Behavioral tests were carried out when mice were 7-weeks old. (**G**) Treated mice were analyzed for their sociability and social novelty preferences in the three-chamber test. *n* = 10 per group. (**H**) Equal amounts of protein lysates from treated mice were immunoblotted for the proteins indicated. Levels of phosphorylated proteins were quantified by densitometry, normalized to respective total protein levels, and compared to WT controls (set to one arbitrary units). *n* = 4 mice per group. (**I**) Equal amounts of hippocampal protein lysates of treated mice were subjected to immunoprecipitation (IP) with IgG and antibodies against BDNF or TrkB, and then immunoblotted for TrkB. Immunoprecipitated TrkB levels were quantified by densitometry, normalized to input levels, and compared to controls (set to one arbitrary unit). *n* = 4 per group. Data represent mean ± SEM. *P*-values were determined by two-tailed unpaired Student *t*-test in (C) and one-way ANOVA with Tukey’s multiple comparisons test in (D, E, G–I). *p < 0.05, **p < 0.01, ***p < 0.001. The data underlying this figure can be found in S1 Data, specifically in the sheet labeled “Figure 6”.

To further study the function of MDGA2 in excitatory neurons in the hippocampus, we packaged AAV2/9 viruses that express a miR30 shRNA against *Mdga2* with enhanced green fluorescent protein (EGFP) under the control of the CaMKIIα promoter (Fig 6A), as well as its control AAVs. AAVs were stereotaxically injected into the hippocampus of 1-week-old C57BL/6 WT mice (Fig 6A and 6F). After 2 weeks, ANA-12 was i.p. delivered at 0.5 mg/kg body weight, twice per week for three consecutive weeks, and mice were subjected to behavioral tests 5 weeks later (Fig 6A). Alternatively, 3 weeks after AAV injection, MDGA2-P2 peptide was bilaterally injected into mouse hippocampus for 1 week. Mice were then studied for their behaviors 2 weeks later (Fig 6F). We confirmed that hippocampal (Fig 6B) injections of AAVs expressing *Mdga2* shRNA specifically reduced MDGA2 levels and activated the TrkB signaling (Fig 6C, 6E, and 6H). Knockdown of MDGA2 in the hippocampus had no effects on mouse locomotor activity (S6A and S6E Fig) or repetitive self-groom behavior (S6B and S6F Fig), but dramatically impaired mouse recognition memory (S6C and S6G Fig) and social behaviors (Figs 6D, 6G, S6D and S6H). While ANA-12 treatment rescued impaired sociability and social novelty deficits (Fig 6D) and hippocampal injection of MDGA2-P2 peptide rescued impaired sociability, social novelty, and social affiliation deficits (Figs 6G and S6H) in these mice. Moreover, both ANA-12 and MDGA2-P2 peptide treatments reversed the elevated TrkB signaling of these mice (Fig 6E and 6H); and MDGA2-P2 peptide treatment also reversed the elevated TrkB-BDNF interaction (Fig 6I) and attenuated the increased evoked AMPAR/NMDAR response ratios caused by MDGA2 knockdown (S6I Fig). However, neither ANA-12 nor the MDGA2-P2 peptide had effect on rescuing impaired recognition memory (S6C and S6G Fig).

Together, these results suggest that MDGA2 of hippocampal excitatory neurons plays a crucial role in memory and social behavior. Moreover, pharmacological inhibition of the TrkB signaling seems to preferentially attenuate social deficits.

### The ASD-associated MDGA2 V930I mutation reduces its membrane anchoring and interaction with TrkB

**Fig 7:**
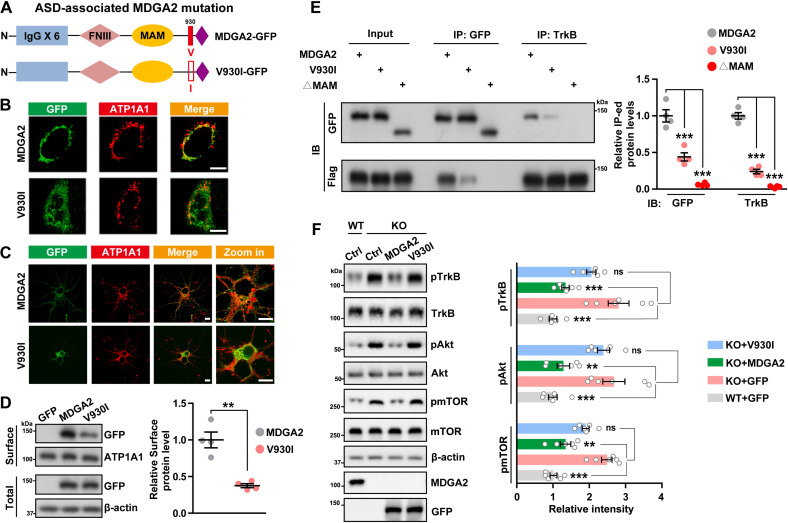
**The** autism spectrum disorder (ASD)-associated MDGA2 V930I mutation impairs the MDGA2-TrkB interaction and MDGA2’s inhibitory effect on the tyrosine kinase B (TrkB) signaling. (**A**) Schematic depiction of the WT human MDGA2 and its V930I mutation constructs that are tagged with GFP. (**B**, **C**) HeLa cells (B) and mouse primary neurons (C) transfected with MDGA2 (green) or V930I (green) were immunostained with an antibody against the plasma membrane protein ATP1A1 (red) and then observed by confocal microscopy. Scale bars = 10 μm. (**D**) Cell surface expression of WT MDGA2 or V930I in transfected HeLa cells was determined by surface biotinylation assays. Biotinylated (cell surface) protein levels were quantified by densitometry and normalized to total protein levels for comparison (WT MDGA2 levels were set to one arbitrary unit). *n* = 4 experimental replicates. (**E**) Mouse primary neurons were transfected with MDGA2, V930I, or ΔMAM. Equal amounts of protein lysates were subjected to immunoprecipitation (IP) with GFP or an anti-TrkB antibody and immunoblotted (IB) for the components indicated. Immunoprecipitated protein levels were quantified by densitometry, normalized to respective inputs, and compared to those transfected with MDGA2 (set to one arbitrary units). *n* = 4 experimental replicates. (**F**) Primary neurons from *Mdga2* ^−/−^ (knock out [KO]) mice were transfected with GFP control (Ctrl), MDGA2 or V930I, and primary neurons from their WT littermates were transfected with GFP control (Ctrl). Equal amounts of protein lysates were immunoblotted for the proteins indicated. Levels of phosphorylated proteins were quantified by densitometry, normalized to respective total protein levels and compared to controls (set to one arbitrary units). *n* = 6 experimental replicates. Data represent mean ± SEM. *P*-values were determined by two-tailed unpaired Student *t* test in (D) and by one-way ANOVA with Tukey’s multiple comparisons test in (E, F). **p < 0.01, ***p < 0.001, ns: not significant. The data underlying this figure can be found in S1 Data, specifically in the sheet labeled “Figure 7”.

A rare MDGA2 missense mutation located in exon 17 of the *MDGA2* gene, which results in the amino acid substitution of V930I was also identified to be associated with ASD [9]. We constructed a GFP-tagged MDGA2-V930I expression plasmid (Fig 7A). Immunostaining studies revealed that when MDGA2-V930I was transfected into HeLa cells (Fig 7B) and mouse primary neurons (Fig 7C), its cell surface distribution was much less than that of WT MDGA2. Consistently, cell biotinylation assays showed that cell surface levels of MDGA2-V930I were significantly lower than those of WT MDGA2 (Fig 7D).

Since both MDGA2 and TrkB are membrane proteins and their interaction is mainly on cell surface (Figs 3C and S3D), we speculate that the V930I mutation may interfere with the MDGA2-TrkB interaction through reducing cell surface levels of MDGA2, thereby causing aberrant TrkB activation. Indeed, in neurons co-transfected with TrkB and WT MDGA2 or MDGA2-V930I, immunoprecipitation assays showed that the interaction between MDGA2-V930I and TrkB was significantly decreased compared to that of WT MDGA2 (Fig 7E). While re-expression of MDGA2-V930I in *Mdga2*^−/−^ neurons did not inhibit the aberrant activation of the TrkB/Akt signaling as WT MDGA2 did (Fig 7F). Collectively, these results indicate that the ASD-associated V930I mutation interferes with MDGA2 subcellular localization possibly through affecting MDGA2 folding, thereby decreases its interaction with TrkB and thus its inhibitory effect on TrkB activity, leading to ASD pathogenesis. Moreover, the V930I mutation may also promote ASD pathogenesis through reducing the MDGA2-NLGN1 interaction; and this deserves further scrutiny.

## Discussion

Truncated and missense mutations in the *MDGA2* gene have been found to associate with ASD. There is evidence that MDGA2 polymorphisms also associate with harm avoidance behaviors [44]. Since MDGA2 is a target of the AF4/AFF member 3 (LAF4/AFF3) transcription factor and its overexpression partially rescues phenotypes caused by LAF4/AFF3 deficiency, MDGA2 may also mediate LAF4/AFF3 deletion-caused neurodevelopmental defects and intellectual disability [45]. Herein, we found that *Mdga2*-deficient mice exhibited ASD-like behaviors including social deficits, repetitive behavior, and cognitive impairment. In addition, *Mdga2*-deficient mice had abnormalities in excitatory synapses, such as increased dendritic spine density and post-synaptic protein levels, and elevated AMPAR-mediated synaptic activity. These results are consistent with a previous study [18] and demonstrate that functional deficiency in MDGA2 leads to ASD pathogenesis.

MDGA2 and its homolog MDGA1 are MAM domain-containing GPI anchor proteins that comprise a novel subgroup of the Ig superfamily [40]. MDGA1 can regulate the radial migration of cortical neurons [46,47] and MDGA2 can regulate rostral growth of commissural axons during neural development [48]. Recent studies also suggest that both MDGA1 and MDGA2 regulate the interaction between NLGNs and NRXNs. Select pairs of NLGNs and NRXNs form *trans-*synaptic bridges spanning the synaptic cleft to regulate synapse development and validation. Among them, neuroligin 2 (NLGN2) is limited to inhibitory synapses and NLGN1 is dominant at excitatory synapses. It has been found that MDGA1 specifically binds to NLGN2 but not NLGN1 or neuroligin 3 (NLGN3) through its Ig domains in a *cis-*manner, thereby blocking the binding of NLGN2 to NRXNs and controlling the function of inhibitory synapses [10–16]. While although previous in vitro studies also found that the Ig domains of MDGA2 interacted with NLGN2 [12,13] and that MDGA2 was a potential specificity factor influencing the recruitment of presynaptic neurotransmitters at inhibitory synapses by NLGN2 [49], another in vivo study showed that MDGA2 preferentially interacts with NLGN1 and restricts excitatory synapse functions [18]. Several recent studies also showed that MDGA2 negatively regulates glutamatergic synapse functions in cultured neurons [19] and that MDGA2 does not affect the abnormal cytosolic Gephyrin aggregation, the reduction in inhibitory synaptic transmission, or the increased anxiety-related behavior in *Nlgn2*-deficient mice [50]. Here, we also found that MDGA2 mainly co-localized with the excitatory postsynaptic protein PSD-95 but not the inhibitory postsynaptic protein Gephyrin and that MDGA2 deficiency specifically affected mEPSCs but not mIPSCs, further supporting the role of MDGA2 in excitatory rather than in inhibitory synapse functions.

Perturbation of the NLGN1-NRXN pathway and the excitatory/inhibitory transmission balance has been suggested as a mechanism underlying the MDGA2 deficiency-caused ASD pathogenesis [17,18]. Herein, we also confirmed that MDGA2 interacted with NLGN1 and that loss of MDGA2 resulted in increased NLGN1 protein levels. More importantly, we demonstrated that aberrant activation of the BDNF/TrkB signaling upon MDGA2 deficiency-mediated disease pathogenesis. Increasing evidence directly or indirectly suggests that the BDNF/TrkB signaling pathway is involved in ASD pathogenesis: a majority of studies found elevated BDNF levels in the serum of ASD patients [28]. Additionally, downstream effectors of TrkB, such as Akt and mTOR, are found to be overactivated in various ASDs [29–31]. Hyperactivation of the Akt/mTOR pathway usually leads to increased synaptic protein synthesis, which is a major cause of abnormal synaptic function and ASD-like behaviors [27,36–39]; and suppression of the Akt/mTOR pathway activity has served as therapeutic targets in multiple ASD models [31,51–55]. We found that BDNF levels were increased in *Mdga2*-deficient mice, accompanied by increased activation of TrkB, Akt, and mTOR. Importantly, treatment with ANA-12 or the MDGA2-P2 peptide to inhibit TrkB activity attenuated social deficits, but had no effect on repetitive behavior or recognition memory in *Mdga2*^ +/−^ mice. Recently, it was also found that Akt and mTOR inhibitors specifically rescued social deficits without affecting repetitive behavior in *Cntnap2−/−* mice [56]. In contrast, some other studies showed that activating the BDNF/TrkB signaling attenuated disease-like phenotypes in several ASD animal models. For example, activation of TrkB by 7.8-dihydroxyflavone rescued both social deficits and repetitive self-grooming behavior in VRK3-deficient mice and in D2-deficient mice that experienced early-life stress [33,35]. Overexpression of BDNF rescued repetitive self-grooming behavior and anxiety-like behavior in neonatally isolated rats [34]. BDNF treatment also attenuated social deficits in valproic acid-exposed mice [32]. Together, these findings including ours suggest that imbalanced BDNF/TrkB activity, either elevated or decreased by various genetic and environmental factors, can lead to complex phenotypes in ASD.

MDGA2-mediated NLGN1-NRXN pathway and BDNF/TrkB signaling may become convergent: it has recently been shown that tyrosine kinase-mediated phosphorylation of NLGN1 promotes the recruitment of PSD scaffold proteins, thereby enhancing AMPAR-mediated synaptic transmission [57]. Herein, we found that MDGA2 deficiency resulted in increased NLGN1 levels, which were reversed when TrkB activity was inhibited. Moreover, knocking down NLGN1 levels in MDGA2-deficient neurons had no effect on TrkB signaling. These findings indicate that the effect of MDGA2 on TrkB signaling is upstream of the NLGN1 signaling.

The complexity of ASD behaviors, characterized by deficits in cognitive and social abilities, as well as stereotypy. The complexity of these deficits suggests a dysregulation involving multiple brain regions [58]. The hippocampus has been identified as a key region closely associated with ASD [59–61]. Consistent with previous reports, our study also observed that MDGA2 deletion specifically resulted in increased numbers of excitatory synapses and excitatory synaptic proteins in the hippocampus. By using AAVs to knockdown MDGA2 expression in the hippocampus, we discovered that MDGA2 deficiency specifically impairs recognition memory and social behavior in mice. These results suggest that the autistic-like behaviors caused by loss function of MDGA2 is primarily due to abnormal excitatory neurons in the hippocampus. Given that MDGA2 is also expressed in considerable amounts in the mPFC, further research is needed to investigate whether MDGA2 serves different functions or exists in different neural circuits in various brain regions.

Although *Mdga2*^ +/−^ mice exhibit social deficits, increased repetitive behavior, and cognitive impairment, blocking the BDNF/TrkB signaling with ANA-12 and the MDGA2-P2 peptide and blocking AMPAR with CP-465022 only attenuated social deficits but not increased repetitive behavior or cognitive impairment in these mice. These findings suggest that MDGA2-regulated BDNF/TrkB signaling may only be responsible for social behaviors. Whether other mechanisms responsible for repetitive behavior and cognitive impairment caused by MDGA2 deficiency exist deserves further scrutiny.

In conclusion, our study demonstrates that under physiological conditions, MDGA2 interacts with TrkB to suppress BDNF-induced TrkB activity and the downstream Akt/mTOR signaling pathway for maintaining normal synaptic protein translation. ASD-associated *MDGA2* mutations and *Mdga2* deletion lead to excessive activation of the BDNF/TrkB-Akt/mTOR signaling and elevated synaptic protein levels, increased AMPAR-mediated excitatory synapse transmission, and disease phenotypes. Both inhibiting the TrkB activity and blocking AMPAR can attenuate social deficits in *Mdga2*^ +/−^ mice (S7 Fig). Our work identifies a new mechanism underlying ASD pathogenesis caused by *MDGA2* mutations and raises an urgent need to evaluate the exact function of the BDNF/TrkB signaling in ASD patients caused by different genetic and environment factors for designing precision treatment.

## Materials and methods

### Reagents and antibodies

Chemical reagents: the TrkB antagonist ANA-12 (Cat# HY-12497) and the AMPAR antagonist CP-465022 (Cat# HY-18663) were purchased from MedChemExpress. Poly-d-lysine (Cat# A3890401) were from Thermo Fisher Scientific. Protease (Cat# 4693132001) and phosphatase (Cat# 4906845001) inhibitor cocktails were purchased from Roche.

Primary antibodies used include: rabbit anti-β-actin (Cell Signaling Technology, Cat# 8457); rabbit anti-Akt (Cell Signaling Technology, Cat# 4685); rabbit anti-pAkt (Cell Signaling Technology, Cat# 4060); rabbit anti-BDNF (Abcam, Cat# ab108319); rabbit anti-CaMKII (Cell Signaling Technology, Cat# 3357S); rabbit anti-pCaMKII (Cell Signaling Technology, Cat# 12716); rabbit anti-Flag (Proteintech, Cat# 20543-1-AP); rabbit anti-GAD1 (Proteintech, Cat# 10408-1-AP); mouse anti-Gephyrin (Synaptic Systems, Cat# 147 011); rabbit anti-Gephyrin (Proteintech, Cat# 12681-1-AP); mouse anti-GFAP (Cell Signaling Technology, Cat# 3670); mouse anti-GFP (Abmart, Cat# M20004); rabbit anti-Iba1 (Cell Signaling Technology, Cat# 17198); rabbit anti-MDGA1 (Biorbyt, Cat# orb773819); rabbit anti-MDGA2 (Abcam, Cat# ab121164, for immunostaining); rabbit anti-MDGA2 (Biorbyt, Cat# orb540377, for immunoblotting); rabbit anti-mTOR (Cell Signaling Technology, Cat# 2983); rabbit anti-pmTOR (Cell Signaling Technology, Cat# 5536); mouse anti-NeuN (Cell Signaling Technology, Cat# 94403); rabbit anti-NLGN1 (Thermo Fisher Scientific, Cat# PA5-78648); mouse anti-phosphotyrosine (Millipore, Cat# 16-316); rabbit anti-PSD-93 (Abcam, Cat# ab151721); mouse anti-PSD-95 (Synaptic Systems, Cat# 124 011); rabbit anti-PSD-95 (Cell Signaling Technology, Cat# 3450S); mouse anti-puromycin antibody (Millipore, Cat# MABE343); rabbit anti-S6 (Cell Signaling Technology, Cat# 2217); rabbit anti-pS6 (Cell Signaling Technology, Cat# 2211); mouse anti-Synaptophysin (Sigma-Aldrich, Cat# S5768); rabbit anti-TrkB (Cell Signaling Technology, Cat# 4603); rabbit anti-pTrkB (Cell Signaling Technology, Cat# 4619); rabbit anti-GluA1 (Cell Signaling Technology, Cat# 13185); rabbit anti-GluN1 (Cell Signaling Technology, Cat# 5704); mouse anti-vGAT (Santa Cruz Biotechnology, Cat# sc-393373); and mouse anti-vGluT1 (Millipore, Cat# MAB5502). The secondary antibodies used include: Goat anti-Mouse IgG (H + L), HRP (ThermoFisher, Cat# 31430); Goat anti-Rabbit IgG (H + L), HRP (ThermoFisher, Cat# 31460); Alexa Fluor 635 Goat anti-Mouse IgG (H + L) (ThermoFisher, Cat# A-31575); Alexa Fluor 488 Goat anti-Rabbit IgG (H + L) (ThermoFisher, Cat#A-11008); and Alexa Fluor 594 Goat anti-Rat IgG (H + L) (ThermoFisher, Cat# A-11007).

### Mice

*Mdga2* heterozygous KO mice (C57BL/6N background) with a deletion of exon 2 were purchased from Cyagen Biosciences (Cat# S-KO-09348) and bred at Xiamen University Laboratory Animal Center. Mouse genotypes were determined by PCR, using mouse-tail DNA as templates and the following primer pair:

Forward: 5′-TCTCCATCTCCTGGTTGTCATTAG-3′
Reverse: 5′-TGCTAATGGCTAGCAAATGCTG-3′

*Mdga2* KO mice and their littermate controls, as well as C57BL/6 WT mice purchased from Xiamen University Laboratory Animal Center were housed in a room maintained at a constant temperature and 12-h light/dark cycle (light hours were 8:00–20:00). Mice were randomly assigned to different experiments used in this study. Male mice were used for quantitative proteomics, drug treatment, and behavioral analyses and following electrophysiological and biochemical experiments. Male and female mice were used for other biochemical experiments. All procedures were in accordance with the National Institutes of Health Guide for the Care and Use of Laboratory Animals and approved by the Animal Ethics Committee of Xiamen University (XMULAC20200163).

### Cell culture

HEK293T, SH-SY5Y, and HeLa cells were originally from ATCC (Manassas, Virginia, USA) and maintained in the laboratory. These cells were cultured in high-glucose DMEM (ThermoFisher) with 10% fetal bovine serum (ThermoFisher). Primary neurons were isolated from both male and female mouse embryos at embryonic day 16.5 (E 16.5) and cultured in neurobasal medium (ThermoFisher) supplemented with 2% B27 (ThermoFisher) and 1 mM glutamine (ThermoFisher).

### Plasmids and siRNAs

Full-length human MDGA2 and its mutant plasmids were generated using a pEGFP-N1 vector backbone. Full-length human TrkB and its mutant cDNAs were cloned into the pcDNA3.1-Flag vector. For BiFC assays, full-length human TrkB was cloned into the pBiFC-VN173 vector containing the N-terminal part of Venus (YouBia, Cat# VT1161), and full-length MDGA2 and its mutant were cloned into the pBiFC-VC155 vector containing the C-terminal part of Venus (YouBia, Cat# VT1162).

NLGN1-targeting siRNA and scrambled control siRNA were synthesized by GenePharma (Shanghai, China). The sequences were as follows: NLGN1 siRNA sense: 5′-CCUGUGUGGU-UCACUAAUATT-3′, NLGN1 siRNA antisense: 5′-UAUUAGUGAACCACACAGGTT-3′. Scrambled control siRNA sense: 5′-UAAUCUUUCACGUAUGGGCTT-3′, Scrambled control siRNA antisense: 5′-GCCCAUACGUGAAAGAUUATT-3′

### Cell transfection

Plasmids were transfected into HEK293T, SH-SY5Y, and HeLa cells using Turbofect transfection reagent (ThermoFisher), following the manufacturer’s instructions. Primary neurons were transfected with plasmids using Lipofectamine LTX (ThermoFisher, Cat# 15338100) or EntransterTM-H4000 transfection reagent (Engreen Bio-system, Cat# 4000-6), and with siRNAs using Lipofectamine RNAiMAX (ThermoFisher, Cat# 13778100), following manufacturers’ protocols. The transfection efficiency of both Lipofectamine LTX and EntransterTM-H4000 exceeded 50%.

### Data-Independent Acquisition (DIA) quantification proteomics and data analysis

The hippocampus of 1.5–2-month-old *Mdga2*^ +/−^ male mice and their WT littermate controls was collected and sent to the BGI company for DIA quantification proteomics and data analysis. Briefly, samples were lysed and sonicated. After centrifugation, the supernatant was taken for quantification. Equal amounts of proteins (100 μg) per sample were subjected to trypsin enzymatic hydrolysis. After dryness, 10 μg enzymatic peptides per sample were separated by high-pH fractionation into a total of 10 fractions using high-performance liquid chromatography (HPLC) and freeze-dried. Data-dependent acquisition (DDA) fractions and DIA samples were analyzed by nano-LC–MS/MS. DDA data was identified by Andromeda search engine within MaxQuant. Identified results were used by Spectronaut^TM^ for spectral library construction. For DIA data, Spectronaut^TM^ used the constructed spectral library information to complete deconvolution and extraction, and used the mProphet algorithm to complete analytical quality control for obtaining reliable quantitative results. The mass spectrometry proteomics data have been deposited to the ProteomeXchange Consortium via the PRIDE [62] partner repository with the dataset identifier PXD050810. DEPs between comparison groups were identified based on the quantitative results, using the fold change >1.1 and *p*-value <0.05 as the criteria. After retrieval from the UniProtKB database, identified DEPs were processed for GO analysis by using DAVID (https://david.ncifcrf.gov/home.jsp) and Metascape database (https://metascape.org/gp/index.html).

### Synaptosome and PSD fraction preparation

Synaptosome and PSD fractions were prepared from both male and female mice at 1.5–2 months of age as previously described [63]. In brief, hippocampal regions were dissected and homogenized in ice-cold sucrose buffer (0.32 M sucrose and 25 mM HEPES, pH 7.4, supplemented with protease and phosphatase inhibitors). After centrifugation at 1,400 *g* for 10 min to remove nuclei and large debris, the supernatant total lysate (S1) fractions were subjected to 10,000 *g* centrifugation for 12 min at 4 °C to obtain the pellet (P2, crude synaptosomal fractions). P2 fractions were washed twice with sucrose buffer, re-suspended in cold HBS buffer (25 mM HEPES, pH 7.4 and 150 mM NaCl, supplemented with protease and phosphatase inhibitors) containing 1% Triton X-100, and centrifuged again at 16,000 *g* for 15 min to obtain the pellet (PSD fractions). PSD fractions were re-suspended in HBS buffer with 3% SDS for further analysis.

### Immunoblotting and co-immunoprecipitation (Co-IP) assays

Equal protein amounts of total lysates from cells or mouse tissues were subjected to SDS-PAGE and immunoblotted with antibodies indicated. For Co-IP, 1 mg of protein lysates were incubated with antibodies indicated and Protein G Agarose (ThermoFisher, Cat# 20399) beads at 4 °C overnight, and immunoprecipitated proteins were then subjected to SDS-PAGE and immunoblotting. Uncropped immunoblotting images are shown in S1 Raw Images.

### TrkB and MDGA2 protein generation

Full-length human TrkB or MDGA2 cDNA was cloned into the pcDNA6-His vector. TrkB and MDGA2 proteins were expressed in HEK293T cells and then purified using a His-tag Protein Purification Kit (Beyotime, Cat# P2229S).

### Pull-down assays

GST pull-down: GST-fused MDGA2 fragments (L1: MDGA2 aa 746−786; L2: MDGA2 aa 787−833) were expressed in bacteria and purified using Glutathione Sepharose 4B (GE Healthcare, Cat# 17-0756-01). Purified human TrkB protein was incubated with GST-fused MDGA2-L1, MDGA2-L2, or GST proteins and then pulled down with Glutathione Sepharose 4B.

Biotin pull-down: Biotin-labeled scrambled peptide (PYPPNTTLKSLPVISFNGKRAA), MDGA2-P1 peptide (NADRSGSKEGFYMYIETSRPRLEGE), and MDGA2-P2 peptide (KARLLSPVFSIAPKNPYGPTNT) were synthesized by Sangon Biotech. Peptides were bilaterally injected into the hippocampal regions of WT mice. Mice were sacrificed 24 h later. Equal protein amounts of hippocampal lysates were precipitated with avidin beads.

### Surface plasmon resonance (SPR) assays

The purified TrkB protein was immobilized on a Biacore CM5 chip (GE Healthcare, BR-1005-30) using the amine coupling kit (GE Healthcare, BR-1000-50) and following the provided instructions. The binding of recombinant MDGA2 protein or MDGA2-P2 peptide was tested at the concentrations indicated, with the response measured in resonance units. Data analysis and *K*_D_ calculation were conducted using the Biacore T100 Evaluation software.

### Immunoprecipitation–mass spectrometry

MDGA2-GFP was expressed in SH-SY5Y cells for 24 h and then lysed in a lysis buffer (10 mM Tris-HCl at pH 7.5, 150 mM NaCl, 0.5 mM EDTA, 0.5% NP-40, and protease inhibitor cocktail). After centrifugation, the supernatant was subjected to immunoprecipitation with an antibody against GFP and Protein G Agarose beads at 4 °C overnight. Immunoprecipitated proteins were subjected to LC–MS/MS analysis performed by Jingjie PTM BioLab.

### Immunostaining and analysis

For brain slices, 8-week-old mice were anesthetized with isoflurane and transcardially perfused with 0.01 M PBS (pH 7.4) followed by 4% paraformaldehyde (PFA) in PBS. Brains were removed and fixed in 4% PFA overnight at 4 °C and placed in 35% sucrose in PBS for 2 days. Brains were then embedded in O.C.T. compound (Tissue-Tek) and cut into 30 μm-thick sections on the freezing microtome of a Leica cryostat. The sections were blocked in 5% bovine serum albumin (BSA) and 0.2% Triton X-100 in PBS for 1 h at room temperature, incubated with primary antibodies overnight at 4 °C, and stained with fluorescent secondary antibodies for 1 h at room temperature. Finally, the slices were counterstained with DAPI for nuclei and cover-slipped with 75% (v/v) glycerol. Images were acquired with identical parameters under a confocal microscope (FV1000MPE-B, Olympus). For three-dimensional rendering of synapses, fluorescent images were captured under a 100× oil immersion objective, with a frame size of 1,024 × 1,024 and a bit depth of 12. Serial images were captured at different depths along the *z*-axis with an optical step of 0.5 μm, resulting in a total of six layers. These images were imported into Imaris 10.0.0 software for analysis. Presynaptic and postsynaptic signals were represented as “spots” and synapses were represented as co-localized presynaptic and postsynaptic signals.

For cell immunostaining, cells were fixed in 4% PFA for 10 min, followed by permeabilization with 0.2% Triton X-100 in PBS for 10 min and blocking in 2% BSA in PBS for 1 h at room temperature. Cells were then incubated with primary antibodies overnight at 4 °C, stained with fluorescent secondary antibodies for 1 h at room temperature, and mounted on slides with Antifade Mounting Medium with DAPI (Beyotime, P0131). Cell images were acquired by confocal microscopy (FV1000MPE-B, Olympus) and High Intelligent and Sensitive SIM (HIS-SIM, Computational SR). All specimens were imaged under identical conditions and analyzed with the Image J software.

### Electron microscope analysis

Eight-week-old male mice were anesthetized with isoflurane and perfused transcardially with PBS followed by 4% PFA and 2% glutaraldehyde in PBS. Brains were removed and fixed in 4% PFA and 2% glutaraldehyde overnight at 4 °C. The hippocampal CA1 region was dissected, fixed in 1% osmium tetroxide, dehydrated in graded alcohols, cut to 70 nm section slices, and stained. Images were obtained on a transmission electron microscope (Hitachi HT-7800). Excitatory (asymmetric PSD density) and inhibitory (symmetric PSD density) synapses, PSD length, and PSD width were identified and counted for comparison using ImageJ (National Institutes of Health).

### Protein–protein interaction prediction

The PDB files for TrkB (AF-Q16620-F1), MDGA2 (AF-Q7Z553-F1) and matured BDNF (AF-P23560-F1) from AlphaFold Protein Structure Database were used. Protein–protein interactions were predicted from the GRAMM: Docking web server (http://gramm.compbio.ku.edu/gramm, Vakser Lab). Structural visualizations of different 3D protein models and their interactions were performed using PYMOL.

### *Mdga2* shRNA AAVs and viral injections

To knockdown *Mdga2* in vivo, an AAV-*Mdga2*-RNAi plasmid was constructed using a vector that contains a mir30shRNA targeting *Mdga2* and a CaMKIIa promoter driving EGFP expression. The sequence of the *Mdga2* shRNA in the AAV-*Mdga2*-RNAi plasmid is 5′-CCCTCAAGATTATGCTAACTA-3′. An AAV-Ctrl-RNAi plasmid with a control sequence of 5′-CTCGCTTGGGCGAGAGTAA-3′ was also used. AAV-*Mdga2*-RNAi and AAV-Ctrl-RNAi plasmids were packaged into AAVs with an AAV2/9 serotype by Obio Technology. Viruses were injected into the hippocampal regions of C57BL/6 WT male mice at postnatal day 6−7. Briefly, mice were cryo-anesthetized on ice for 3−5 min. Following cessation of movement, viruses were injected into bilateral hippocampal regions with 7.5 × 109 viral particles on each side using a 10-μL syringe (Hamilton, 7642-01) with a 30-G needle (Hamilton, 7803-07). After the injections were completed, pups were placed on a warming pad until they regained normal skin tone and resumed movement. All injected pups were then returned to their mothers for care and further recovery.

### Stereotaxic peptide injection

Four-week-old male mice were anesthetized and fixed to a stereotactic mouse frame (RWD Life Science). Two small holes were made in the skull, and guide cannulas (RWD Life Science, 62004) were implanted simultaneously to ventral hippocampus (AP, − 2.0 mm; L, ± 2.3 mm; DV, − 2.0 mm) and fixed to the skull with two metal screws and dental cement. To prevent clogging, stainless wires were placed into the cannula. Three days after mouse recovery, the stainless wires were replaced with an injector (RWD Life Science, 62104) connected to Hamilton syringes, which was extended 1 mm beyond the tip of the guide cannula. One μL MDGA2-P2 peptide (1 μg/μL, dissolved in saline) or scrambled peptide (1 μg/μL, dissolved in saline) was injected stereotaxically over 5 min (0.20 μL/min), and the needle was kept in place for another 5 min after injection. Injection was performed once per day for seven consecutive days.

### Quantitative real-time PCR

Total RNA was extracted using TRIzol reagent (Thermo Fisher Scientific, Cat# 15596026) following the manufacturer’s instructions. cDNA was synthesized using the ReverTra Ace qPCR RT Kit (TOYOBO). Quantitative real-time PCR was performed using FastStart Universal SYBR Green Master Mix (ROX) (Roche, Cat# 04913850001) in combination with relevant gene-specific primers; β-actin served as an internal control. PCR primers used for qRT-PCR were as the following:

mouse *Mdga2* forward primer: 5′-CCAGAGGCCTATGAAGTCCG-3′,
mouse *Mdga2* reverse primer: 5′-TCCACAGTGAAATTCCCTCAA-3′;
mouse β-actin forward primer: 5′-AGCCATGTACGTAGCCATCCA-3′,
mouse β-actin reverse primer: 5′-TCTCCGGAGTCCATCACAATG-3′.

### Pharmacological treatment

ANA-12 was dissolved in saline containing 10% DMSO to a final concentration of 0.5 mg/kg and intraperitoneally administered into male mice. For chronic treatment, ANA-12 was administered to 3-week-old male mice at twice/week for 3 weeks until mice reached 6 weeks of age. For acute treatment, ANA-12 was administered to 4-week-old male mice for three consecutive days. For and cultured neurons, ANA-12 was suspended to a 10 mM concentration in DMSO and diluted 1:1,000 in solution. Final concentration for treatment is 10 µ M with 0.1% DMSO.

CP-465022 was dissolved in saline containing 10% DMSO to a final concentration of 1 mg/kg and intraperitoneally administered into male mice. Sixty minutes later, mice were evaluated for behaviors.

### SUnSET puromycin treatment

To measure de novo protein synthesis in primary neurons, we used the SUnSET puromycin incorporation assay described previously [37,64]. Briefly, puromycin (10 μg/ml) was applied to cultured neurons for 1 h, and the cultured neurons were then lysed and used for immunoblotting. Puromycin-incorporated proteins were detected using an anti-puromycin antibody.

### Electrophysiological recordings

Electrophysiological recordings were performed following published protocols [65,66] with some modifications. Briefly, acute hippocampal slices from 8-week-old treated *Mdga2*^ +/−^ male mice and WT littermate controls were recorded for mEPSCs and mIPSCs in the presence of 1 μM TTX at holding potentials of −70 and 0 mV. The pipette was filled with an internal solution containing 140 mM CsMeSO_3_, 5 mM TEA-Cl, 10 mM HEPES, 1 mM EGTA, 2.5 mM ATP magnesium salt, 0.3 mM GTP sodium salt, and 2 mM MgCl_2_·6H_2_O (pH 7.3, 298–300 mOsm).

AMPAR-mediated currents were recorded in the presence of 50 μM AP-5 and 20 μM bicuculline at a holding membrane potential of − 70 mV. NMDAR-mediated currents were recorded in the presence of 20 μM CNQX and 20 μM bicuculline at a holding membrane potential of +40 mV. Responses were evoked every 30 s and recorded at a stimulus intensity of 30–300 μA. Alternatively, for recording both AMPAR- and NMDAR-mediated currents within a same cell, only 20 μM bicuculline was used to inhibit GABA receptors. The AMPAR/NMDAR ratio was calculated as the ratio of the average EPSC peak amplitude recorded at − 70 mV (for AMPAR) to the average amplitude (for those at 50 ms after the peak of the current response, when the contribution of the AMPAR current was minimal [65]) of EPSCs recorded at +40 mV. The pipette for AMPAR and NMDAR EPSC recording was filled with an internal solution containing 140 mM CsMeSO_3_, 5 mM TEA-Cl, 10 mM HEPES, 1 mM EGTA, 2.5 mM ATP magnesium salt, 0.3 mM GTP sodium salt, 2 mM MgCl2·6H_2_O, and 5 mM QX-314 (pH 7.3, 298–300 mOsm).

### Behavioral experiments

Six- to eight-week-old male mice were subjected to various behavioral tests. Mice were allowed to acclimatize in the testing room for 1 h prior to each test. Data were recorded and analyzed using Smart 3.0 video tracking system (Panlab, Harvard Apparatus).

#### Open field test.

Mice were gently placed in the center of an open field apparatus (40 × 40 × 40 cm), and allowed for a 10-min period of free movement. Total distance traveled and time spent in center were analyzed.

#### Novel location recognition (NLR).

This test consists of habituation, training, and testing phases. Mice were first put into the open field apparatus described above for 10 min habituation. During the training phase, mice were allowed to freely explore two identical objects placed near two opposite corners of the open field arena for 8 min. Twenty-four hour later, one of the objects was moved to be close to a third corner (N: Novel location) while the other one was kept in the same spatial position (F: Familiar location). Mice were then tested for their exploration behavior with these objects for 8 min. The discrimination index (T(N − F)/T(N + F): time spent exploring the novel location minus time spent in familiar location over time spent to both novel and familiar location) was calculated to assess the recognition memory of mice.

#### Social affiliation test.

The test procedure was similar to that described previously [18,67]. Briefly, a cylinder (8.5 cm in diameter) with a gender-matched reference mouse confined was placed at a specific location in an open field (40 cm in diameter). Tested mice were allowed to move freely in the open field for 7 min and their sniffing time with the reference mice was analyzed.

#### Three-chamber test.

This test was performed as described previously [18,67] with minor modifications. The apparatus (60 W × 40 D × 22 H cm) has two transparent partitions separating the left, the middle, and the right chambers (20 × 40 cm each), and each partition has a small opening (5 × 5 cm) for access to each chamber. A fixed wire cylinder (8.5 cm diameter) was placed in each of the left and the right chambers. This test consisted of three phases: habituation, sociability test, and social novelty test. During habituation, both left and right cylinders were empty, and tested mice were placed in the center and allowed to explore freely for 10 min. During the sociability test, a gender-matched strange mouse (S1) was enclosed in one empty cylinder, and mice were allowed to freely explore for 10 min. Their time spent sniffing S1 or the other empty cylinder (E) was measured and the sociability preference index (T(S1 − -E)/T(S1 + E): time spent sniffing S1 minus time spent sniffing E over time spent sniffing both S1 and E) was calculated for comparison. During the social novelty test, another gender-matched unfamiliar mouse (S2) was placed into the empty cylinder and mice were allowed to freely explore for another 10 min. Their time spent sniffing S1 or S2 was measured and the social novelty preference index (T(S2 − S1)/T(S2 + S1): time spent sniffing S2 minus time spent sniffing S1 over time spent sniffing both S2 and S1) was calculated for comparison.

#### Self-grooming test.

Tested mice were placed individually in a clean cage with fresh bedding. After 5 min of habituation, the spontaneous behaviors of mice were recorded for 30 min and their self-grooming behavior was analyzed.

#### Nest-building test.

A square cotton (3 g) was placed into a cage with fresh bedding. Tested mice were placed individually into a cage with fresh bedding and a square cotton (3 g) as bedding material. The next morning, nest building was scored according to the guidelines as described previously [39].

### Statistical analysis

Mouse behaviors were studied in a double-blind manner (neither the experimenters nor the analysts were aware of the experimental group assignments). Other experiments were studied in a single-blind manner (the experimenters but not the analysts knew the experimental group assignments). Statistical analysis was performed using GraphPad Prism 5 or 8 (GraphPad Software, La Jolla, California, USA). All data represent mean ± SEM. No inclusion/exclusion criteria were applied to datasets. Differences between two groups were analyzed using unpaired *t*-test. For multiple comparisons, differences were assessed by one-way ANOVA followed by Tukey’s post hoc test or one-way ANOVA followed by Dunnett’s post hoc test. For data with more than one independent variable, two-way ANOVA with Tukey’s post hoc test was used. Detailed test used for each experiment was described in the figure legends. *p* < 0.05 was considered to be statistically significant.

## Supporting information

S1 Fig MDGA2 expression pattern and *Mdga2* knockout (KO) mouse generation. (**A**) MDGA2 protein levels were detected by immunoblotting in medial prefrontal cortex (mPFC), hippocampus, and cerebellum from 1-month-old wild-type (WT) mice. β-actin served as a loading control. (**B**) MDGA2 protein levels in P0 mouse primary neurons, astrocytes, and microglia were detected by immunoblotting. NeuN, GFAP, and Iba1 were used as markers for neurons, astrocytes, and microglia, respectively. β-actin served as a loading control. (**C**) MDGA2 protein levels in the mouse cerebrum during different developmental periods were detected by immunoblotting. β-actin served as a loading control. (**D**) Co-immunostaining of MDGA2 (green) with PSD-95 (red) or Gephyrin (red) in cultured mouse primary neurons at DIV14. Scale bars = 10 μm. (**E**) Schematic depiction of the *Mdga2* gene knockout strategy. The *Mdga2* gene containing 17 exons is located on mouse chromosome 12, with the ATG start codon in exon 1 and the TGA stop codon in exon 17 (Transcript: ENSMUST00000037181). Exon 2 is selected as a target site for knockout. (**F**) Genotyping of *Mdga2*^ +/−^ (Het) mice, *Mdga2*^−/−^ (KO) mice, and their WT littermates. (**G**) *Mdga2* mRNA levels in the brain of Het and KO mice and their littermates were determined by quantitative real-time PCR, normalized to respective β-actin levels, and compared to WT values (set to one arbitrary unit, indicated by dashed line). (**H**) Equal amounts of brain protein lysates were subjected to immunoblotting to determine MDGA2 and MDGA1 levels in Het and KO mice and their WT littermates. MDGA2 protein levels were quantified by densitometry, normalized to respective β-actin levels, and compared to WT values (set to one arbitrary unit, indicated by dashed line). (**I**) Co-immunostaining of MDGA2 (green) with PSD-95 (red) or Gephyrin (pink) in the hippocampus of MDGA2 complete KO mice and their WT littermates. The nuclei were stained with DAPI (blue). Scale bars = 200 μm. Data represent mean ± SEM, *n* = 3 per group in (A–C), and *n* = 4 per group in (G, H). *P*-values were determined by one-way ANOVA with Tukey’s multiple comparisons test. *p < 0.05, **p < 0.01, ***p < 0.001, ns: not significant. The data underlying this figure can be found in S1 Data, specifically in the sheet labeled “Supplementary Figure 1”.
(TIF)

S2 Fig MDGA2 deficiency results in aberrant tyrosine kinase B (TrkB) activation and synaptic protein production that can be reversed by ANA-12 treatment. (**A**) Primary neurons from *Mdga2*^ +/−^ (Het), *Mdga2*^−/−^ (KO), and WT littermate mice were treated with saline or 10 μM ANA-12 for 24 h at 12 DIV, and equal protein amounts of cells lysates were immunoblotted for the proteins indicated. In some experiments, 10 μg/ml puromycin was added to the media 1 h before the end of ANA-12 treatment. (**B**) Total levels of proteins indicated were quantified by densitometry, normalized to those of β-actin, and compared to those of WT (set to one arbitrary units). The levels of phosphorylated (p) proteins were normalized to respective total protein levels and then compared to those of WT controls (set to one arbitrary units). *n* = 3 replicates per group. Data represent means ± SEM. *P*-values were determined by one-way ANOVA with Tukey’s multiple comparisons test. *p < 0.05, **p < 0.01, ***p < 0.001, ****p < 0.0001, ns: not significant. The data underlying this figure can be found in S1 Data, specifically in the sheet labeled “Supplementary Figure 2”.
(TIF)

S3 Fig MDGA2 competes with brain-derived neurotrophic factor (BDNF) for tyrosine kinase B (TrkB) binding. (**A**) Primary hippocampal neurons from *Mdga2*^ +/−^ (Het), *Mdga2*^−/−^ (KO), and wild-type (WT) littermate mice at DIV 7 were treated with 50 ng/ml BDNF for 30 min. Equal amounts of protein lysates were subjected to immunoprecipitation (IP) with an anti-tyrosine kinase B (TrkB) antibody, followed by immunoblotting (IB) with an anti-phosphotyrosine (pTyr) antibody or the anti-TrkB antibody. The pTyr levels were quantified by densitometry, normalized to total TrkB levels, and then compared to those of WT littermates. *n* = 4 per group. (**B**) MDGA2-GFP was expressed in SH-SY5Y cells for 24 h. Cell lysates were subjected to IP with an anti-GFP antibody and Protein G Agarose beads at 4 °C overnight. Immunoprecipitated proteins were then subjected to LC–MS/MS analysis. (**C**) WT mouse brain lysates were subjected to IP with an anti-TrkB antibody and a control IgG, and immunoblotted (IB) for the components indicated. (**D**) SH-SY5Y cells were immunostained with antibodies against TrkB (red) and MDGA2 (green), and then counter-stained with DAPI (blue). Images were acquired by High Sensitivity Structured Illumination Microscope (HiS-SIM). Scale bar: 10 μm. (**E**) HeLa cells were transfected with MDGA2-GFP or MDGA2-ΔMAM-GFP for 24 h. Cells were fixed and counterstained with DAPI (blue). Images were acquired by HiS-SIM. Scale bars: 10 μm. (**F**) Computer simulation of the interaction between TrkB and mature BDNF (mBDNF). The binding domain of TrkB (the d5 domain) is highlighted by a red square and labeled in brown. (**G**) A scheme of full-length TrkB shows its C1 domain, three LRR domains, C2 domain, IgG1 domain, and IgG2/d5 domain. The scheme of TrkB-Δd5 lacking the IgG2/d5 domain is also shown. (**H**) HEK293T cells were co-transfected with MDGA2-GFP and Flag-tagged full-length TrkB (TrkB-Flag) or TrkB-Δd5 (Δd5-Flag). Cell lysates were subjected to IP with control IgG or antibodies against Flag or GFP, and IB for the components indicated. (**I**) Equal amounts of protein lysates from 1-month-old Het and their WT littermate mouse brains were subjected to IP with IgG or an anti-TrkB antibody, and then IB for the components indicated. Immunoprecipitated BDNF levels in Het samples were quantified by densitometry, normalized to input levels, and compared to WT controls (set to one arbitrary units). *n* = 4 per group. (**J**) WT neurons were transfected with GFP control (Ctrl), MDGA2-GFP, or MDGA2-ΔMAM-GFP for 24 h, and then treated with 50 ng/ml BDNF for 30 min. Equal amounts of protein lysates were subjected to IB for the proteins indicated. Levels of phosphorylated proteins were quantified by densitometry, normalized to respective total protein levels and compared to controls (set to one arbitrary units). *n* = 6 per group. (**K**) Hippocampal neurons of Het mice and their WT littermates were treated with either vehicle or NLGN1 siRNA. Equal amounts of protein lysates were immunoblotted to detect the proteins indicated. Levels of phosphorylated proteins were quantified by densitometry, normalized to respective total protein levels and compared to controls (set to one arbitrary units). *n* = 4 per group. Data represent mean ± SEM. *P*-values were determined by one-way ANOVA with Dunnett’s multiple comparisons test in (A), two-tailed unpaired Student *t*-test in (I), and one-way ANOVA with Tukey’s multiple comparisons test in (J, K). *p < 0.05, **p < 0.01, ***p < 0.001, ns: not significant. The data underlying this figure can be found in S1 Data, specifically in the sheet labeled “Supplementary Figure 3”.
(TIF)

S4 Fig Acute ANA-12 treatment temporarily rescues social deficits in *Mdga2* ^+/−^ mice. (**A**) ANA-12 was intraperitoneally injected into 3-week-old Het mice and their wild-type (WT) littermates at 0.5 mg/kg body weight. After different time points, mice were sacrificed and equal protein amounts of cerebrum lysates were subjected to immunoblotting for components indicated. (**B**) ANA-12 acute treatment scheme. ANA-12 was intraperitoneally injected into 4-week-old Het mice and their WT littermates at 0.5 mg/kg body weight for three consecutive days. Animal behavioral tests were performed on day 4 (Test 1) and day 10 (Test 2), respectively. (**C**–**G**) Mice were analyzed for their total travel distance in the open field test (C), their discrimination index in the novel location recognition test (D), their time spent self-grooming and bouts of self-grooming (E), their sniffing time in the social affiliation test (F), and their sociability and social novelty preferences in the three-chamber test (G). *n* = 15 mice per group. Data represent mean ± SEM. *P*-values were determined by two-way ANOVA with Tukey’s multiple comparisons test. ns: not significant, *p < 0.05, **p < 0.01, ***p < 0.001, ns: not significant. For Test 1: Interaction *F*(1,56) = 0.3413, *p = *0.5614, and for Test 2: Interaction *F*(1,56) = 0.1904, *p = *0.6643 in C. For Test 1: Interaction *F*(1,56) = 3.941, *p = *0.0520, and for Test 2: Interaction *F*(1,56) = 0.9635, *p = *0.3305 in D. For Test 1: Interaction *F*(1,56) = 0.6611, *p = *0.4196, and for Test 2: Interaction *F*(1,56) = 0.4466, *p = *0.5067 in E. For Test 1: Interaction *F*(1,56) = 7.214, *p = *0.0095, and for Test 2: Interaction *F*(1,56) = 0.9759, *p = *0.3275 in F. For Test 1: Interaction *F*(1,56) = 9.336, *p = *0.0034, and for Test 2: Interaction *F*(1,56) = 1.622, *p = *0.2081 in G left. For Test 1: Interaction *F*(1,56) = 0.9906, *p = *0.3239, and for Test 2: Interaction *F*(1,56) = 0.8065, *p = *0.3730 in G right. The data underlying this figure can be found in S1 Data, specifically in the sheet labeled “Supplementary Figure 4”.
(TIF)

S5 Fig Pharmacological inhibition of AMPAR improves social behaviors in *Mdga2* ^+/−^ mice. (**A**, **B**) The miniature inhibitory postsynaptic currents (mIPSCs) of hippocampal neurons in treated Het mice and their wild-type (WT) littermates were recorded and their kinetics (rise time and decay) were quantified for comparison. *n* = 14 cells from 3 mice per group. (**C**, **D**) The miniature excitatory postsynaptic currents (mEPSCs) of hippocampal neurons in treated mice were recorded and their kinetics (rise time and decay) were quantified for comparison. *n* = 14 cells from 3 mice per group. (**E**) Representative traces of evoked NMDAR-mediated EPSCs and quantitative analysis of evoked NMDAR-mediated EPSC amplitude with increasing stimulus intensity. *n* = 20–23 cells from 3 mice per group. (**F**) AMPAR- and NMDAR-mediated EPSCs were recorded and their ratios were calculated for comparison. *n* = 24 cells from 3 mice per group. (**G**) Hippocampal neurons of *Mdga2*^ +/−^ (Het), *Mdga2*^−/−^ (KO), and their WT littermate mice were treated with vehicle or 10 μM ANA-12 for 24 h at 12 DIV, then subjected to cell surface biotinylation and immunoblotting of the proteins indicated. (**H**) The CP-465022 treatment scheme. CP-465022 or saline control was intraperitoneally injected into 8-week-old *Mdga2*^ +/−^ (Het) mice at 1 mg/kg body weight. Sixty min later, mice were evaluated for behaviors. (**I**–**M**) Treated mice were studied for their sniffing time in the social affiliation test (I), their sociability preference (J) and social novelty preference (K) in the three-chamber test, their discrimination index in the novel location recognition test (L), and their time spent self-grooming and bouts of self-grooming (M). *n* = 10 mice per group. Data represent mean ± SEM. *P*-values were determined by two-way ANOVA with Tukey’s multiple comparisons test in (A–D, F), repeated-measures ANOVA followed by Bonferroni’s post hoc analysis in (E), and two-tailed unpaired Student *t*-test in (I–M). *p < 0.05, **p < 0.01, ns: not significant. Interaction *F*(1,52) = 7.382, *p = *0.0089 in A; Interaction *F*(1,52) = 0.2293, *p = *0.6340 in B; Interaction *F*(1,52) = 0.1152, *p = *0.7357 in C; Interaction *F*(1,52) = 0.1500, *p = *0.7001 in D; Interaction *F*(1,92) = 3.337, *p = *0.0710 in F. The data underlying this figure can be found in S1 Data, specifically in the sheet labeled “Supplementary Figure 5”.
(TIF)

S6 Fig Inhibition of tyrosine kinase B (TrkB) activity alleviates social behaviors in hippocampal MDGA2-deficient mice. (**A**–**I**) Mice were subjected to hippocampal infection with AAVs, and then treated with ANA-12 (A–D) or MDGA2-P2 peptide (E–I). Treated mice were analyzed for their total travel distance and time spent in the center in the open field test (A, E), their time spent self-grooming and bouts of self-grooming (B, F), their discrimination index in the novel location recognition (NLR) test (C, G), and their time spent sniffing a reference mouse in the social affiliation test (D, H). *n* = 10 mice per group. AMPAR- and NMDAR-mediated EPSCs were recorded and their ratios were calculated for comparison (I). *n* = 16 cells from 3 mice per group. Data represent mean ± SEM. *P*-values were determined by one-way ANOVA with Tukey’s multiple comparisons test. *p < 0.05, ns: not significant. The data underlying this figure can be found in S1 Data, specifically in the sheet labeled “Supplementary Figure 6”.
(TIF)

S7 Fig A scheme showing the mechanism underlying MDGA2-mediated brain-derived neurotrophic factor (BDNF)/tyrosine kinase B (TrkB) signaling pathway. Under physiological conditions, the MAM domain of MDGA2 interacts with the d5 domain of TrkB to suppress BDNF-TrkB binding. Therefore, MDGA2 keeps the BDNF/TrkB signaling at bay for maintaining a normal Akt/mTOR signaling and downstream synaptic protein translation. MDGA2 deficiency in mice and mutations in ASD patients lead to excessive activation of the BDNF/TrkB/Akt/mTOR signaling and elevated synaptic protein production, resulting in increased excitatory synapse transmission and ASD-associated behavioral phenotypes. Inhibiting TrkB activity by ANA-12 or MDGA2 MAM domain peptide can reduce the aberrantly activated Akt/mTOR signaling, elevated synaptic protein levels and excitatory synapse transmission, and thus ASD-associated social deficits in *Mdga2*-deficient mice.
(TIF)

S1 Table DIA quantification proteomics to compare protein expression differences in the hippocampus of wild-type and *Mdga2* ^+/−^ mice. (XLSX)

S2 Table Upregulated DEPs in *Mdga2* ^+/−^ versus wild-type samples. (XLSX)

S3 Table Downregulated DEPs in *Mdga2* ^+/−^ versus wild-type samples. (XLSX)

S1 Data Source data of Figs 1A, 1B, 1C, 1D, 1E, 1F, 1G, 1H, 2C, 2D, 2F, 2G, 2I, 2J, 3A, 3H, 3I, 3J, 4H, 4J, 4K, 4L, 4M, 4N, 4O, 5B, 5C, 5D, 5E, 5F, 5G, 5H, 5I, 5J, 6C, 6D, 6E, 6G, 6H, 6I, 7D, 7E, 7F, S1A, S1B, S1C, S1G, S1H, S2B, S3A, S3I, S3J, S3K, S4C, S4D, S4E, S4F, S4G, S5A, S5B, S5C, S5D, S5E, S5F, S5I, S5J, S5K, S5L, S5M, S6A, S6B, S6C, S6D, S6E, S6F, S6G, S6H, and S6I. (XLSX)

S1 Raw Images Uncropped blots for immunoblotting data. (PDF)

## Abbreviations

**:**
ASD: autism spectrum disorder
BDNF: brain-derived neurotrophic factor
BSA: bovine serum albumin
Co-IP: co-immunoprecipitation
DDA: data-dependent acquisition
DEPs: differentially expressed proteins
DIA: data-independent acquisition
FITC: fluorescein isothiocyanate
FNIII: fibronectin type III-like
ImBDNF: immature BDNF
i.p.: intraperitoneal
MAM: memprin/A5/mu
mBDNF: mature BDNF
mEPSC: miniature excitatory postsynaptic current
mIPSC: miniature inhibitory postsynaptic current
mPFC: medial prefrontal cortex
NLGN1: neuroligin 1
NLGN2: neuroligin 2
NLGN3: neuroligin 3
NLR: Novel location recognition
NRXN: neurexin
PFA: paraformaldehyde
PSD: postsynaptic density
SPR: surface plasmon resonance
TrkB: tyrosine kinase B

## References

[1] Mullins C, Fishell G, Tsien RW. Unifying views of autism spectrum disorders: a consideration of autoregulatory feedback loops. Neuron. 2016;89(6):1131–56. doi: 10.1016/j.neuron.2016.02.017 26985722 PMC5757244

[2] Won H, Mah W, Kim E. Autism spectrum disorder causes, mechanisms, and treatments: focus on neuronal synapses. Front Mol Neurosci. 2013;6:19. doi: 10.3389/fnmol.2013.00019 23935565 PMC3733014

[3] Bhat S, Acharya UR, Adeli H, Bairy GM, Adeli A. Autism: cause factors, early diagnosis and therapies. Rev Neurosci. 2014;25(6):841–50. doi: 10.1515/revneuro-2014-0056 25222596

[4] Folstein SE, Rosen-Sheidley B. Genetics of autism: complex aetiology for a heterogeneous disorder. Nat Rev Genet. 2001;2(12):943–55. doi: 10.1038/35103559 11733747

[5] Glessner JT, Wang K, Cai G, Korvatska O, Kim CE, Wood S, et al. Autism genome-wide copy number variation reveals ubiquitin and neuronal genes. Nature. 2009;459(7246):569–73. doi: 10.1038/nature07953 19404257 PMC2925224

[6] Peça J, Feng G. Cellular and synaptic network defects in autism. Curr Opin Neurobiol. 2012;22(5):866–72. doi: 10.1016/j.conb.2012.02.015 22440525 PMC3407823

[7] Leblond CS, Nava C, Polge A, Gauthier J, Huguet G, Lumbroso S, et al. Meta-analysis of SHANK mutations in autism spectrum disorders: a gradient of severity in cognitive impairments. PLoS Genet. 2014;10(9):e1004580. doi: 10.1371/journal.pgen.1004580 25188300 PMC4154644

[8] Bucan M, Abrahams BS, Wang K, Glessner JT, Herman EI, Sonnenblick LI, et al. Genome-wide analyses of exonic copy number variants in a family-based study point to novel autism susceptibility genes. PLoS Genet. 2009;5(6):e1000536. doi: 10.1371/journal.pgen.1000536 19557195 PMC2695001

[9] De Rubeis S, He X, Goldberg AP, Poultney CS, Samocha K, Cicek AE, et al. Synaptic, transcriptional and chromatin genes disrupted in autism. Nature. 2014;515(7526):209–15. doi: 10.1038/nature13772 25363760 PMC4402723

[10] Elegheert J, Cvetkovska V, Clayton AJ, Heroven C, Vennekens KM, Smukowski SN, et al. Structural mechanism for modulation of synaptic neuroligin-neurexin signaling by MDGA proteins. Neuron. 2017;95(4):896-913.e10. doi: 10.1016/j.neuron.2017.07.040 28817804 PMC5563082

[11] Connor SA, Elegheert J, Xie Y, Craig AM. Pumping the brakes: suppression of synapse development by MDGA-neuroligin interactions. Curr Opin Neurobiol. 2019;57:71–80. doi: 10.1016/j.conb.2019.01.002 30771697

[12] Pettem KL, Yokomaku D, Takahashi H, Ge Y, Craig AM. Interaction between autism-linked MDGAs and neuroligins suppresses inhibitory synapse development. J Cell Biol. 2013;200(3):321–36. doi: 10.1083/jcb.201206028 23358245 PMC3563690

[13] Lee K, Kim Y, Lee S-J, Qiang Y, Lee D, Lee HW, et al. MDGAs interact selectively with neuroligin-2 but not other neuroligins to regulate inhibitory synapse development. Proc Natl Acad Sci U S A. 2013;110(1):336–41. doi: 10.1073/pnas.1219987110 23248271 PMC3538197

[14] Kim J, Wulschner LEG, Oh WC, Ko J. Trans-synaptic mechanisms orchestrated by mammalian synaptic cell adhesion molecules. Bioessays. 2022;44(11):e2200134. doi: 10.1002/bies.202200134 36089658

[15] Gangwar SP, Zhong X, Seshadrinathan S, Chen H, Machius M, Rudenko G. Molecular mechanism of MDGA1: regulation of Neuroligin 2:Neurexin trans-synaptic bridges. Neuron. 2017;94(6):1132–41.e4. doi: 10.1016/j.neuron.2017.06.009 28641112 PMC5745067

[16] Kim JA, Kim D, Won SY, Han KA, Park D, Cho E, et al. Structural insights into modulation of Neurexin-Neuroligin trans-synaptic adhesion by MDGA1/Neuroligin-2 complex. Neuron. 2017;94(6):1121–31.e6. doi: 10.1016/j.neuron.2017.05.034 28641111

[17] Toledo A, Letellier M, Bimbi G, Tessier B, Daburon S, Favereaux A, et al. MDGAs are fast-diffusing molecules that delay excitatory synapse development by altering neuroligin behavior. Elife. 2022;11:e75233. doi: 10.7554/eLife.75233 35532105 PMC9084894

[18] Connor SA, Ammendrup-Johnsen I, Chan AW, Kishimoto Y, Murayama C, Kurihara N, et al. Altered cortical dynamics and cognitive function upon haploinsufficiency of the autism-linked excitatory synaptic suppressor MDGA2. Neuron. 2016;91(5):1052–68. doi: 10.1016/j.neuron.2016.08.016 27608760

[19] Kim S, Jang G, Kim H, Lim D, Han KA, Um JW, et al. MDGAs perform activity-dependent synapse type-specific suppression via distinct extracellular mechanisms. Proc Natl Acad Sci U S A. 2024;121(26):e2322978121. doi: 10.1073/pnas.2322978121 38900791 PMC11214077

[20] Bryn V, Halvorsen B, Ueland T, Isaksen J, Kolkova K, Ravn K, et al. Brain derived neurotrophic factor (BDNF) and autism spectrum disorders (ASD) in childhood. Eur J Paediatr Neurol. 2015;19(4):411–4. doi: 10.1016/j.ejpn.2015.03.005 25847750

[21] Connolly AM, Chez M, Streif EM, Keeling RM, Golumbek PT, Kwon JM, et al. Brain-derived neurotrophic factor and autoantibodies to neural antigens in sera of children with autistic spectrum disorders, Landau-Kleffner syndrome, and epilepsy. Biol Psychiatry. 2006;59(4):354–63. doi: 10.1016/j.biopsych.2005.07.004 16181614

[22] Meng W-D, Sun S-J, Yang J, Chu R-X, Tu W, Liu Q. Elevated serum brain-derived neurotrophic factor (BDNF) but not BDNF gene Val66Met polymorphism is associated with autism spectrum disorders. Mol Neurobiol. 2017;54(2):1167–72. doi: 10.1007/s12035-016-9721-9 26820673

[23] Nishimura K, Nakamura K, Anitha A, Yamada K, Tsujii M, Iwayama Y, et al. Genetic analyses of the brain-derived neurotrophic factor (BDNF) gene in autism. Biochem Biophys Res Commun. 2007;356(1):200–6. doi: 10.1016/j.bbrc.2007.02.135 17349978

[24] Ebert DH, Greenberg ME. Activity-dependent neuronal signalling and autism spectrum disorder. Nature. 2013;493(7432):327–37. doi: 10.1038/nature11860 23325215 PMC3576027

[25] Enriquez-Barreto L, Morales M. The PI3K signaling pathway as a pharmacological target in autism related disorders and Schizophrenia. Mol Cell Ther. 2016;4:2. doi: 10.1186/s40591-016-0047-9 26877878 PMC4751644

[26] Yeung KS, Tso WWY, Ip JJK, Mak CCY, Leung GKC, Tsang MHY, et al. Identification of mutations in the PI3K-AKT-mTOR signalling pathway in patients with macrocephaly and developmental delay and/or autism. Mol Autism. 2017;8:66. doi: 10.1186/s13229-017-0182-4 29296277 PMC5738835

[27] Yoshii A, Constantine-Paton M. Postsynaptic BDNF-TrkB signaling in synapse maturation, plasticity, and disease. Dev Neurobiol. 2010;70(5):304–22. doi: 10.1002/dneu.20765 20186705 PMC2923204

[28] Armeanu R, Mokkonen M, Crespi B. Meta-analysis of BDNF levels in autism. Cell Mol Neurobiol. 2017;37(5):949–54. doi: 10.1007/s10571-016-0415-7 27501933 PMC11482231

[29] Winden KD, Ebrahimi-Fakhari D, Sahin M. Abnormal mTOR activation in autism. Annu Rev Neurosci. 2018;41:1–23. doi: 10.1146/annurev-neuro-080317-061747 29490194

[30] McMahon JJ, Yu W, Yang J, Feng H, Helm M, McMahon E, et al. Seizure-dependent mTOR activation in 5-HT neurons promotes autism-like behaviors in mice. Neurobiol Dis. 2015;73:296–306. doi: 10.1016/j.nbd.2014.10.004 25315683 PMC4394017

[31] Tai C, Chang C-W, Yu G-Q, Lopez I, Yu X, Wang X, et al. Tau reduction prevents key features of autism in mouse models. Neuron. 2020;106(3):421–37.e11. doi: 10.1016/j.neuron.2020.01.038 32126198 PMC7210056

[32] Qi C, Chen A, Mao H, Hu E, Ge J, Ma G, et al. Excitatory and inhibitory synaptic imbalance caused by brain-derived neurotrophic factor deficits during development in a valproic acid mouse model of autism. Front Mol Neurosci. 2022;15:860275. doi: 10.3389/fnmol.2022.860275 35465089 PMC9019547

[33] Lee Y, Han P-L. Early-life stress in D2 heterozygous mice promotes autistic-like behaviors through the downregulation of the BDNF-TrkB pathway in the dorsal striatum. Exp Neurobiol. 2019;28(3):337–51. doi: 10.5607/en.2019.28.3.337 31308794 PMC6614072

[34] Bahi A. Hippocampal BDNF overexpression or microR124a silencing reduces anxiety- and autism-like behaviors in rats. Behav Brain Res. 2017;326:281–90. doi: 10.1016/j.bbr.2017.03.010 28284951

[35] Kang M-S, Choi T-Y, Ryu HG, Lee D, Lee S-H, Choi S-Y, et al. Autism-like behavior caused by deletion of vaccinia-related kinase 3 is improved by TrkB stimulation. J Exp Med. 2017;214(10):2947–66. doi: 10.1084/jem.20160974 28899869 PMC5626391

[36] Aryal S, Longo F, Klann E. Genetic removal of p70 S6K1 corrects coding sequence length-dependent alterations in mRNA translation in fragile X syndrome mice. Proc Natl Acad Sci U S A. 2021;118(18):e2001681118. doi: 10.1073/pnas.2001681118 33906942 PMC8106352

[37] Xu Z-X, Kim GH, Tan J-W, Riso AE, Sun Y, Xu EY, et al. Elevated protein synthesis in microglia causes autism-like synaptic and behavioral aberrations. Nat Commun. 2020;11(1):1797. doi: 10.1038/s41467-020-15530-3 32286273 PMC7156673

[38] Santini E, Huynh TN, MacAskill AF, Carter AG, Pierre P, Ruggero D, et al. Exaggerated translation causes synaptic and behavioural aberrations associated with autism. Nature. 2013;493(7432):411–5. doi: 10.1038/nature11782 23263185 PMC3548017

[39] Kalinowska M, van der Lei MB, Kitiashvili M, Mamcarz M, Oliveira MM, Longo F, et al. Deletion of Fmr1 in parvalbumin-expressing neurons results in dysregulated translation and selective behavioral deficits associated with fragile X syndrome. Mol Autism. 2022;13(1):29. doi: 10.1186/s13229-022-00509-2 35768828 PMC9245312

[40] Litwack ED, Babey R, Buser R, Gesemann M, O’Leary DDM. Identification and characterization of two novel brain-derived immunoglobulin superfamily members with a unique structural organization. Mol Cell Neurosci. 2004;25(2):263–74. doi: 10.1016/j.mcn.2003.10.016 15019943

[41] Thoumine O, Marchot P. A triad of crystals sheds light on MDGA interference with neuroligation. Neuron. 2017;95(4):729–32. doi: 10.1016/j.neuron.2017.08.001 28817794

[42] Haniu M, Montestruque S, Bures EJ, Talvenheimo J, Toso R, Lewis-Sandy S, et al. Interactions between brain-derived neurotrophic factor and the TRKB receptor. Identification of two ligand binding domains in soluble TRKB by affinity separation and chemical cross-linking. J Biol Chem. 1997;272(40):25296–303. doi: 10.1074/jbc.272.40.25296 9312147

[43] Kim J-W, Park K, Kang RJ, Gonzales ELT, Kim DG, Oh HA, et al. Pharmacological modulation of AMPA receptor rescues social impairments in animal models of autism. Neuropsychopharmacology. 2019;44(2):314–23. doi: 10.1038/s41386-018-0098-5 29899405 PMC6300529

[44] Heck A, Pfister H, Czamara D, Müller-Myhsok B, Pütz B, Lucae S, et al. Evidence for associations between MDGA2 polymorphisms and harm avoidance: replication and extension of a genome-wide association finding. Psychiatr Genet. 2011;21(5):257–60. doi: 10.1097/YPG.0b013e3283457bfb 21399569

[45] Moore JM, Oliver PL, Finelli MJ, Lee S, Lickiss T, Molnár Z, et al. Laf4/Aff3, a gene involved in intellectual disability, is required for cellular migration in the mouse cerebral cortex. PLoS One. 2014;9(8):e105933. doi: 10.1371/journal.pone.0105933 25162227 PMC4146563

[46] Ishikawa T, Gotoh N, Murayama C, Abe T, Iwashita M, Matsuzaki F, et al. IgSF molecule MDGA1 is involved in radial migration and positioning of a subset of cortical upper-layer neurons. Dev Dyn. 2011;240(1):96–107. doi: 10.1002/dvdy.22496 21104742

[47] Takeuchi A, O’Leary DDM. Radial migration of superficial layer cortical neurons controlled by novel Ig cell adhesion molecule MDGA1. J Neurosci. 2006;26(17):4460–4. doi: 10.1523/JNEUROSCI.4935-05.2006 16641224 PMC6674065

[48] Joset P, Wacker A, Babey R, Ingold EA, Andermatt I, Stoeckli ET, et al. Rostral growth of commissural axons requires the cell adhesion molecule MDGA2. Neural Dev. 2011;6:22. doi: 10.1186/1749-8104-6-22 21542908 PMC3113314

[49] Loh KH, Stawski PS, Draycott AS, Udeshi ND, Lehrman EK, Wilton DK, et al. Proteomic analysis of unbounded cellular compartments: synaptic clefts. Cell. 2016;166(5):1295–307.e21. doi: 10.1016/j.cell.2016.07.041 27565350 PMC5167540

[50] Zeppillo T, Ali H, Ravichandran S, Ritter TC, Wenger S, López-Murcia FJ, et al. Functional Neuroligin-2-MDGA1 interactions differentially regulate synaptic GABAARs and cytosolic gephyrin aggregation. Commun Biol. 2024;7(1):1157. doi: 10.1038/s42003-024-06789-z 39284869 PMC11405390

[51] Chen C-J, Sgritta M, Mays J, Zhou H, Lucero R, Park J, et al. Therapeutic inhibition of mTORC2 rescues the behavioral and neurophysiological abnormalities associated with Pten-deficiency. Nat Med. 2019;25(11):1684–90. doi: 10.1038/s41591-019-0608-y 31636454 PMC7082835

[52] Iezzi D, Curti L, Ranieri G, Gerace E, Costa A, Ilari A, et al. Acute rapamycin rescues the hyperexcitable phenotype of accumbal medium spiny neurons in the valproic acid rat model of autism spectrum disorder. Pharmacol Res. 2022;183:106401. doi: 10.1016/j.phrs.2022.106401 35987482

[53] Liu D, Nanclares C, Simbriger K, Fang K, Lorsung E, Le N, et al. Autistic-like behavior and cerebellar dysfunction in Bmal1 mutant mice ameliorated by mTORC1 inhibition. Mol Psychiatry. 2023;28(9):3727–38. doi: 10.1038/s41380-022-01499-6 35301425 PMC9481983

[54] Pagani M, Barsotti N, Bertero A, Trakoshis S, Ulysse L, Locarno A, et al. mTOR-related synaptic pathology causes autism spectrum disorder-associated functional hyperconnectivity. Nat Commun. 2021;12(1):6084. doi: 10.1038/s41467-021-26131-z 34667149 PMC8526836

[55] Poopal AC, Schroeder LM, Horn PS, Bassell GJ, Gross C. Increased expression of the PI3K catalytic subunit p110δ underlies elevated S6 phosphorylation and protein synthesis in an individual with autism from a multiplex family. Mol Autism. 2016;7:3. doi: 10.1186/s13229-015-0066-4 26770665 PMC4712554

[56] Xing X, Zhang J, Wu K, Cao B, Li X, Jiang F, et al. Suppression of Akt-mTOR pathway rescued the social behavior in Cntnap2-deficient mice. Sci Rep. 2019;9(1):3041. doi: 10.1038/s41598-019-39434-5 30816216 PMC6395585

[57] Letellier M, Szíber Z, Chamma I, Saphy C, Papasideri I, Tessier B, et al. A unique intracellular tyrosine in neuroligin-1 regulates AMPA receptor recruitment during synapse differentiation and potentiation. Nat Commun. 2018;9(1):3979. doi: 10.1038/s41467-018-06220-2 30266896 PMC6162332

[58] Müller R-A. The study of autism as a distributed disorder. Ment Retard Dev Disabil Res Rev. 2007;13(1):85–95. doi: 10.1002/mrdd.20141 17326118 PMC3315379

[59] Yizhar O, Fenno LE, Prigge M, Schneider F, Davidson TJ, O’Shea DJ, et al. Neocortical excitation/inhibition balance in information processing and social dysfunction. Nature. 2011;477(7363):171–8. doi: 10.1038/nature10360 21796121 PMC4155501

[60] Barnea-Goraly N, Frazier TW, Piacenza L, Minshew NJ, Keshavan MS, Reiss AL, et al. A preliminary longitudinal volumetric MRI study of amygdala and hippocampal volumes in autism. Prog Neuropsychopharmacol Biol Psychiatry. 2014;48:124–8. doi: 10.1016/j.pnpbp.2013.09.010 24075822 PMC8655120

[61] Dager SR, Wang L, Friedman SD, Shaw DW, Constantino JN, Artru AA, et al. Shape mapping of the hippocampus in young children with autism spectrum disorder. AJNR Am J Neuroradiol. 2007;28(4):672–7. 17416819 PMC7977363

[62] Perez-Riverol Y, Bai J, Bandla C, García-Seisdedos D, Hewapathirana S, Kamatchinathan S, et al. The PRIDE database resources in 2022: a hub for mass spectrometry-based proteomics evidences. Nucleic Acids Res. 2022;50(D1):D543–52. doi: 10.1093/nar/gkab1038 34723319 PMC8728295

[63] Zhao D, Meng J, Zhao Y, Huo Y, Liu Y, Zheng N, et al. RPS23RG1 is required for synaptic integrity and rescues Alzheimer’s disease-associated cognitive deficits. Biol Psychiatry. 2019;86:171–84. https://doi:10.1016/j.biopsych.2018.08.009. 30292394 10.1016/j.biopsych.2018.08.009 PMC6389446

[64] Schmidt EK, Clavarino G, Ceppi M, Pierre P. SUnSET, a nonradioactive method to monitor protein synthesis. Nat Methods. 2009;6(4):275–7. doi: 10.1038/nmeth.1314 19305406

[65] Wang X, Lin D, Jiang J, Liu Y, Dong X, Fan J, et al. MDGA2 constrains glutamatergic inputs selectively onto CA1 pyramidal neurons to optimize neural circuits for plasticity, memory, and social behavior. Neurosci Bull. 2024;40(7):887–904. doi: 10.1007/s12264-023-01171-1 38321347 PMC11250762

[66] Gao Y, Hong Y, Huang L, Zheng S, Zhang H, Wang S, et al. β2-microglobulin functions as an endogenous NMDAR antagonist to impair synaptic function. Cell. 2023;186(5):1026–38.e20. doi: 10.1016/j.cell.2023.01.021 36868208

[67] Qin L, Ma K, Wang Z-J, Hu Z, Matas E, Wei J, et al. Social deficits in Shank3-deficient mouse models of autism are rescued by histone deacetylase (HDAC) inhibition. Nat Neurosci. 2018;21(4):564–75. doi: 10.1038/s41593-018-0110-8 29531362 PMC5876144

