# Supplementary figures and images for: Mdga2 deficiency leads to an aberrant activation of BDNF/TrkB signaling that underlies autism-relevant synaptic and behavioral changes in mice
Source: PLoS Biol. 2025 Apr 1;23(4):e3003047. doi: 10.1371/journal.pbio.3003047 (PMC11960969; doi:10.1371/journal.pbio.3003047)

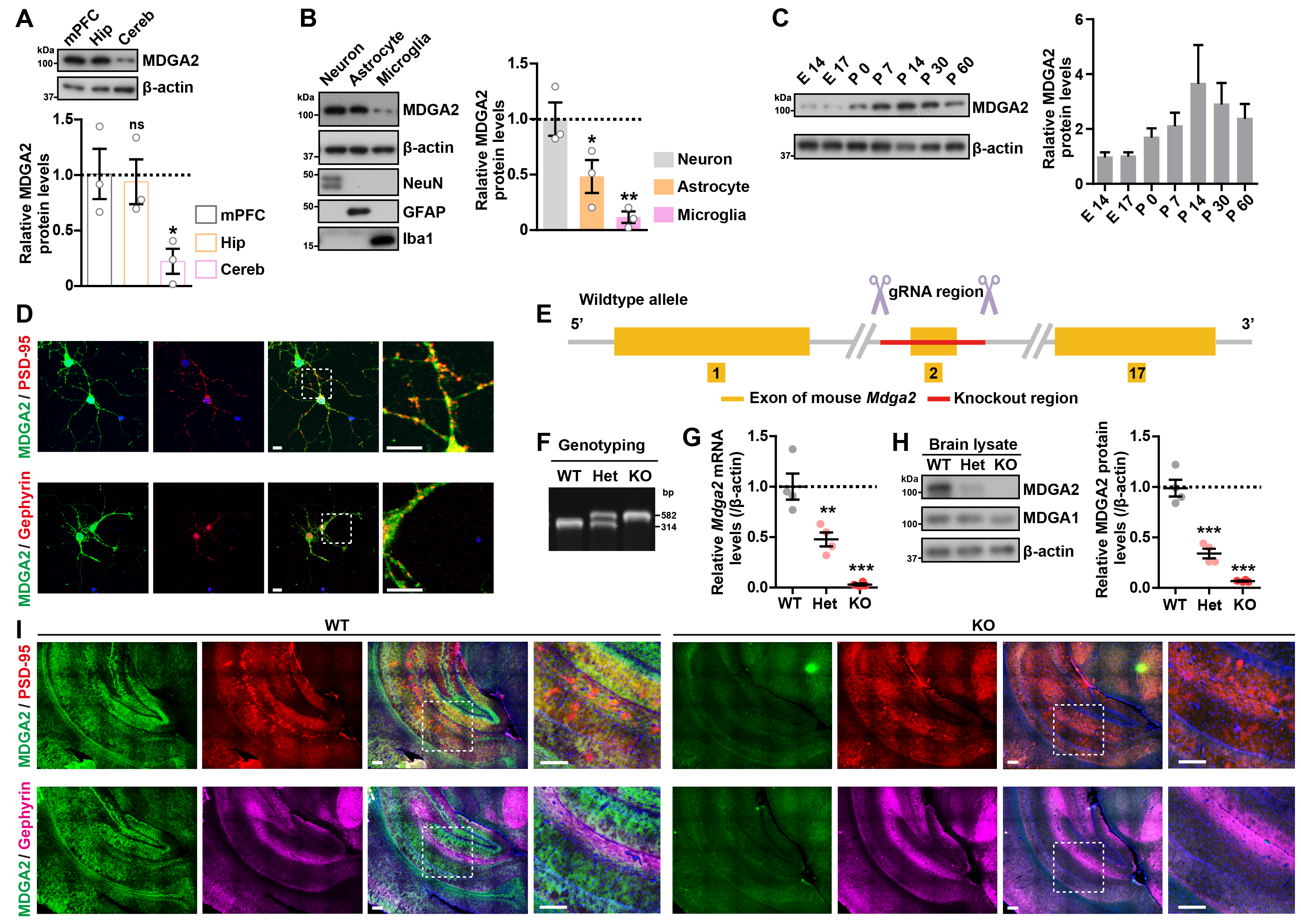

Supplement: S1 Fig — (A) MDGA2 protein levels were detected by immunoblotting in medial prefrontal cortex (mPFC), hippocampus, and cerebellum from 1-month-old wild-type (WT) mice. β-actin served as a loading control. (B) MDGA2 protein levels in P0 mouse primary neurons, astrocytes, and microglia were detected by immunoblotting. NeuN, GFAP, and Iba1 were used as markers for neurons, astrocytes, and microglia, respectively. β-actin served as a loading control. (C) MDGA2 protein levels in the mouse cerebrum during different developmental periods were detected by immunoblotting. β-actin served as a loading control. (D) Co-immunostaining of MDGA2 (green) with PSD-95 (red) or Gephyrin (red) in cultured mouse primary neurons at DIV14. Scale bars = 10 μm. (E) Schematic depiction of the Mdga2 gene knockout strategy. The Mdga2 gene containing 17 exons is located on mouse chromosome 12, with the ATG start codon in exon 1 and the TGA stop codon in exon 17 (Transcript: ENSMUST00000037181). Exon 2 is selected as a target site for knockout. (F) Genotyping of Mdga2 +/− (Het) mice, Mdga2−/− (KO) mice, and their WT littermates. (G) Mdga2 mRNA levels in the brain of Het and KO mice and their littermates were determined by quantitative real-time PCR, normalized to respective β-actin levels, and compared to WT values (set to one arbitrary unit, indicated by dashed line). (H) Equal amounts of brain protein lysates were subjected to immunoblotting to determine MDGA2 and MDGA1 levels in Het and KO mice and their WT littermates. MDGA2 protein levels were quantified by densitometry, normalized to respective β-actin levels, and compared to WT values (set to one arbitrary unit, indicated by dashed line). (I) Co-immunostaining of MDGA2 (green) with PSD-95 (red) or Gephyrin (pink) in the hippocampus of MDGA2 complete KO mice and their WT littermates. The nuclei were stained with DAPI (blue). Scale bars = 200 μm. Data represent mean ± SEM, n = 3 per group in (A–C), and n = 4 per group in (G, H). P-values were determi [file pbio.3003047.s001.tif]

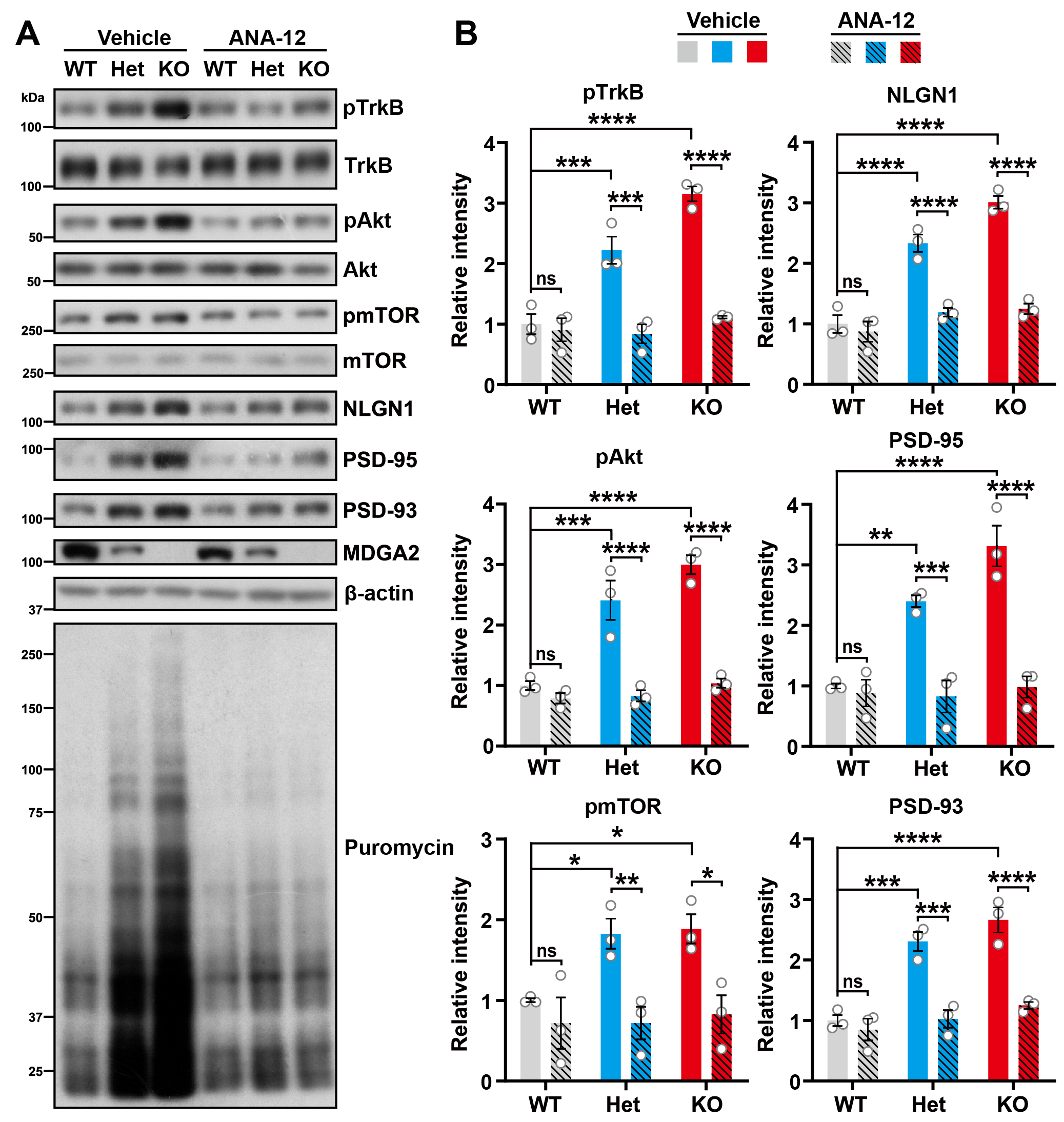

Supplement: S2 Fig — (A) Primary neurons from Mdga2 +/− (Het), Mdga2−/− (KO), and WT littermate mice were treated with saline or 10 μM ANA-12 for 24 h at 12 DIV, and equal protein amounts of cells lysates were immunoblotted for the proteins indicated. In some experiments, 10 μg/ml puromycin was added to the media 1 h before the end of ANA-12 treatment. (B) Total levels of proteins indicated were quantified by densitometry, normalized to those of β-actin, and compared to those of WT (set to one arbitrary units). The levels of phosphorylated (p) proteins were normalized to respective total protein levels and then compared to those of WT controls (set to one arbitrary units). n = 3 replicates per group. Data represent means ± SEM. P-values were determined by one-way ANOVA with Tukey’s multiple comparisons test. *p < 0.05, **p < 0.01, ***p < 0.001, ****p < 0.0001, ns: not significant. The data underlying this figure can be found in S1 Data, specifically in the sheet labeled “Supplementary Figure 2”. (TIF) [file pbio.3003047.s002.tif]

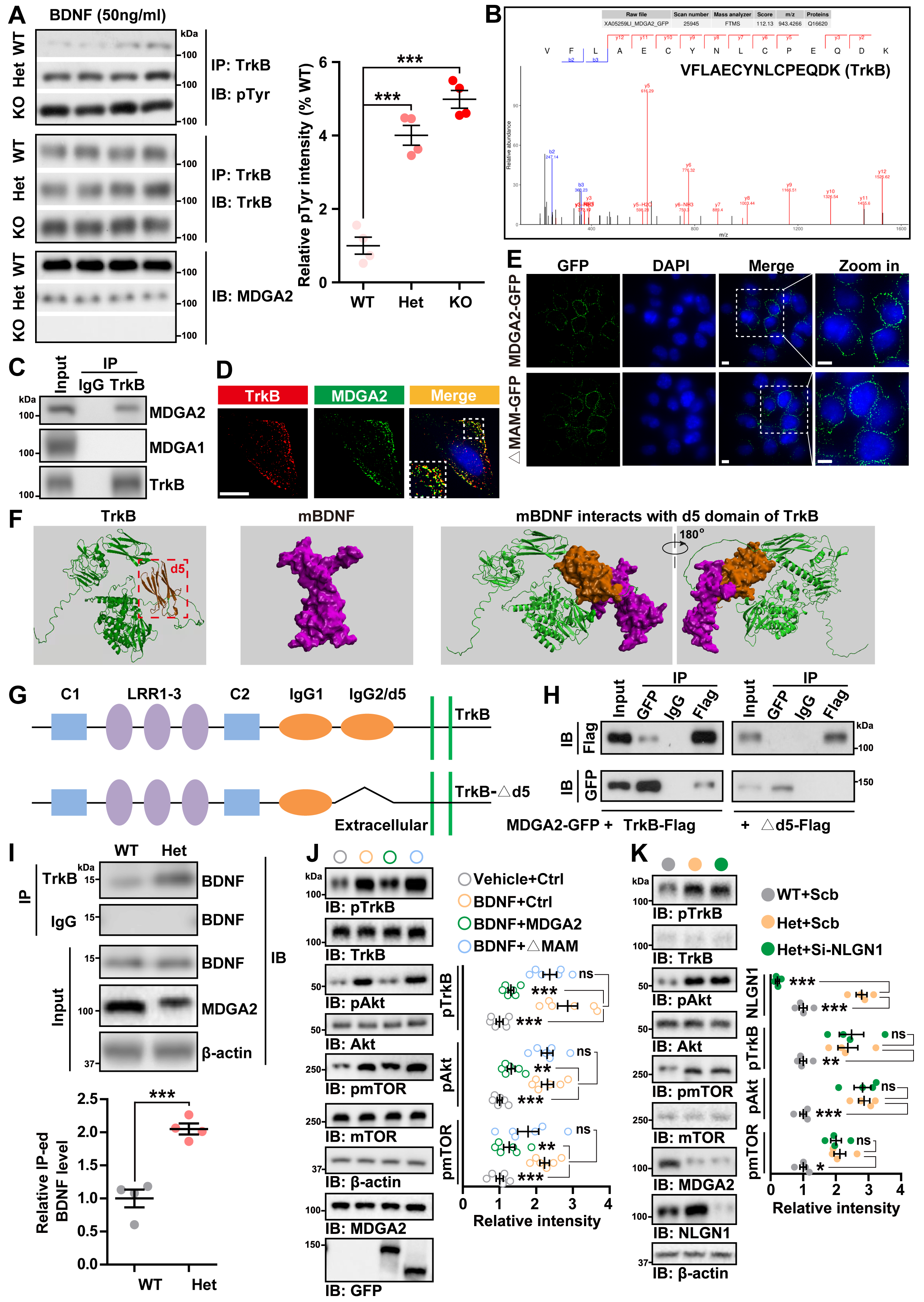

Supplement: S3 Fig — (A) Primary hippocampal neurons from Mdga2 +/− (Het), Mdga2−/− (KO), and wild-type (WT) littermate mice at DIV 7 were treated with 50 ng/ml BDNF for 30 min. Equal amounts of protein lysates were subjected to immunoprecipitation (IP) with an anti-tyrosine kinase B (TrkB) antibody, followed by immunoblotting (IB) with an anti-phosphotyrosine (pTyr) antibody or the anti-TrkB antibody. The pTyr levels were quantified by densitometry, normalized to total TrkB levels, and then compared to those of WT littermates. n = 4 per group. (B) MDGA2-GFP was expressed in SH-SY5Y cells for 24 h. Cell lysates were subjected to IP with an anti-GFP antibody and Protein G Agarose beads at 4 °C overnight. Immunoprecipitated proteins were then subjected to LC–MS/MS analysis. (C) WT mouse brain lysates were subjected to IP with an anti-TrkB antibody and a control IgG, and immunoblotted (IB) for the components indicated. (D) SH-SY5Y cells were immunostained with antibodies against TrkB (red) and MDGA2 (green), and then counter-stained with DAPI (blue). Images were acquired by High Sensitivity Structured Illumination Microscope (HiS-SIM). Scale bar: 10 μm. (E) HeLa cells were transfected with MDGA2-GFP or MDGA2-ΔMAM-GFP for 24 h. Cells were fixed and counterstained with DAPI (blue). Images were acquired by HiS-SIM. Scale bars: 10 μm. (F) Computer simulation of the interaction between TrkB and mature BDNF (mBDNF). The binding domain of TrkB (the d5 domain) is highlighted by a red square and labeled in brown. (G) A scheme of full-length TrkB shows its C1 domain, three LRR domains, C2 domain, IgG1 domain, and IgG2/d5 domain. The scheme of TrkB-Δd5 lacking the IgG2/d5 domain is also shown. (H) HEK293T cells were co-transfected with MDGA2-GFP and Flag-tagged full-length TrkB (TrkB-Flag) or TrkB-Δd5 (Δd5-Flag). Cell lysates were subjected to IP with control IgG or antibodies against Flag or GFP, and IB for the components indicated. (I) Equal amounts of protein lysates from 1-month-old Het and their [file pbio.3003047.s003.tif]

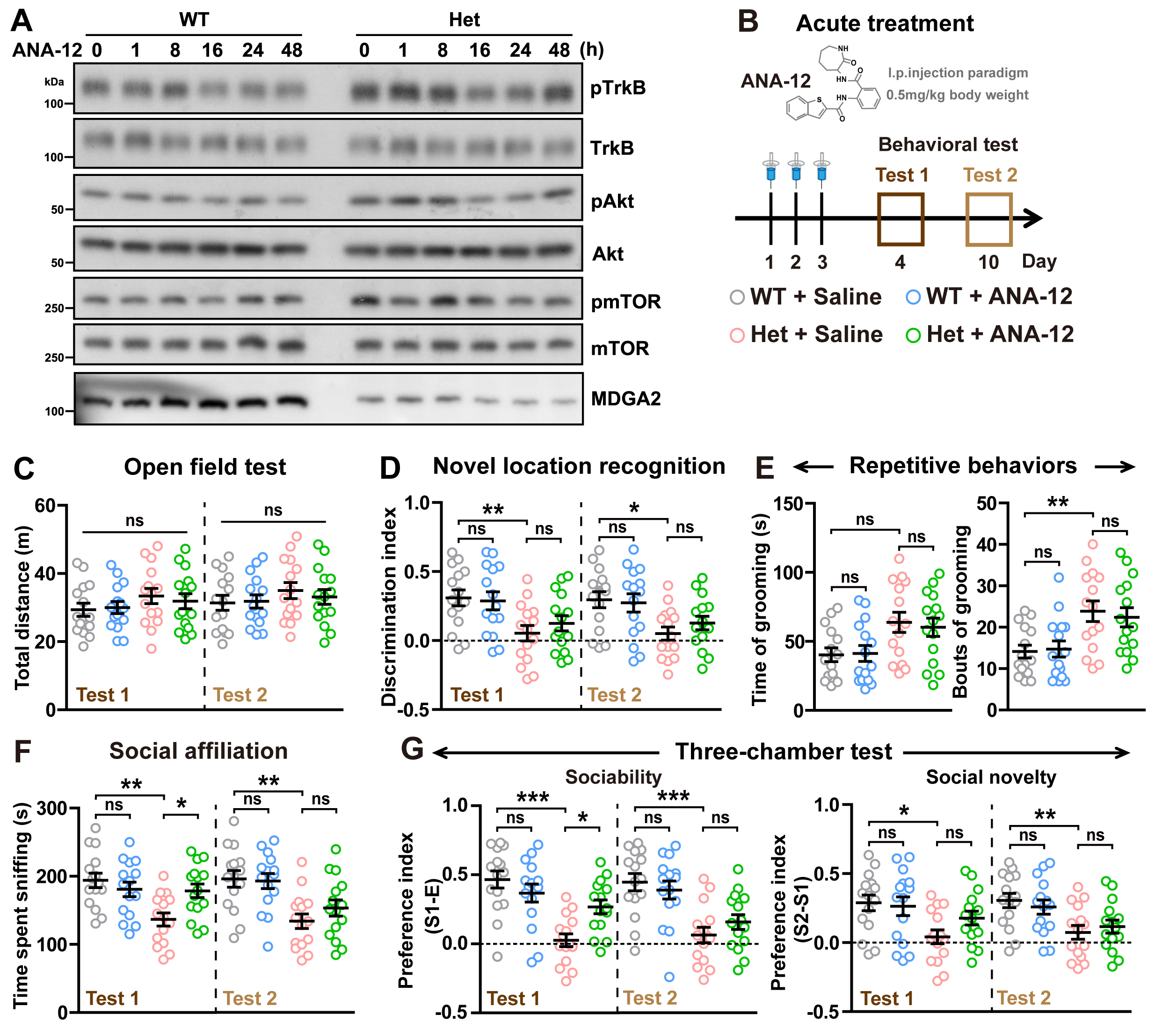

Supplement: S4 Fig — (A) ANA-12 was intraperitoneally injected into 3-week-old Het mice and their wild-type (WT) littermates at 0.5 mg/kg body weight. After different time points, mice were sacrificed and equal protein amounts of cerebrum lysates were subjected to immunoblotting for components indicated. (B) ANA-12 acute treatment scheme. ANA-12 was intraperitoneally injected into 4-week-old Het mice and their WT littermates at 0.5 mg/kg body weight for three consecutive days. Animal behavioral tests were performed on day 4 (Test 1) and day 10 (Test 2), respectively. (C–G) Mice were analyzed for their total travel distance in the open field test (C), their discrimination index in the novel location recognition test (D), their time spent self-grooming and bouts of self-grooming (E), their sniffing time in the social affiliation test (F), and their sociability and social novelty preferences in the three-chamber test (G). n = 15 mice per group. Data represent mean ± SEM. P-values were determined by two-way ANOVA with Tukey’s multiple comparisons test. ns: not significant, *p < 0.05, **p < 0.01, ***p < 0.001, ns: not significant. For Test 1: Interaction F(1,56) = 0.3413, p = 0.5614, and for Test 2: Interaction F(1,56) = 0.1904, p = 0.6643 in C. For Test 1: Interaction F(1,56) = 3.941, p = 0.0520, and for Test 2: Interaction F(1,56) = 0.9635, p = 0.3305 in D. For Test 1: Interaction F(1,56) = 0.6611, p = 0.4196, and for Test 2: Interaction F(1,56) = 0.4466, p = 0.5067 in E. For Test 1: Interaction F(1,56) = 7.214, p = 0.0095, and for Test 2: Interaction F(1,56) = 0.9759, p = 0.3275 in F. For Test 1: Interaction F(1,56) = 9.336, p = 0.0034, and for Test 2: Interaction F(1,56) = 1.622, p = 0.2081 in G left. For Test 1: Interaction F(1,56) = 0.9906, p = 0.3239, and for Test 2: Interaction F(1,56) = 0.8065, p = 0.3730 in G right. The data underlying this figure can be found in S1 Data, specifically in the sheet labeled “Supplementary Figure 4”. (TIF) [file pbio.3003047.s004.tif]

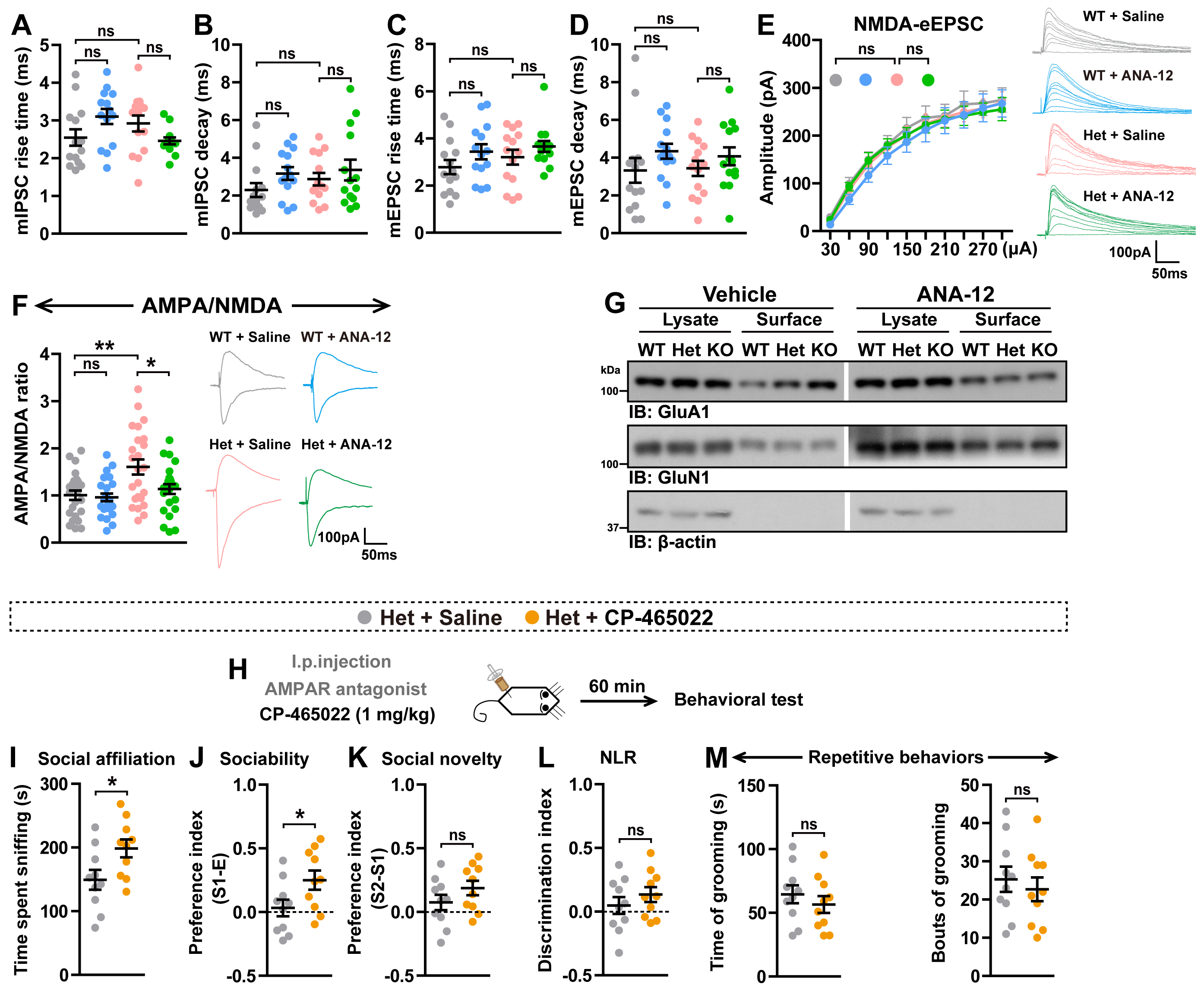

Supplement: S5 Fig — (A, B) The miniature inhibitory postsynaptic currents (mIPSCs) of hippocampal neurons in treated Het mice and their wild-type (WT) littermates were recorded and their kinetics (rise time and decay) were quantified for comparison. n = 14 cells from 3 mice per group. (C, D) The miniature excitatory postsynaptic currents (mEPSCs) of hippocampal neurons in treated mice were recorded and their kinetics (rise time and decay) were quantified for comparison. n = 14 cells from 3 mice per group. (E) Representative traces of evoked NMDAR-mediated EPSCs and quantitative analysis of evoked NMDAR-mediated EPSC amplitude with increasing stimulus intensity. n = 20–23 cells from 3 mice per group. (F) AMPAR- and NMDAR-mediated EPSCs were recorded and their ratios were calculated for comparison. n = 24 cells from 3 mice per group. (G) Hippocampal neurons of Mdga2 +/− (Het), Mdga2−/− (KO), and their WT littermate mice were treated with vehicle or 10 μM ANA-12 for 24 h at 12 DIV, then subjected to cell surface biotinylation and immunoblotting of the proteins indicated. (H) The CP-465022 treatment scheme. CP-465022 or saline control was intraperitoneally injected into 8-week-old Mdga2 +/− (Het) mice at 1 mg/kg body weight. Sixty min later, mice were evaluated for behaviors. (I–M) Treated mice were studied for their sniffing time in the social affiliation test (I), their sociability preference (J) and social novelty preference (K) in the three-chamber test, their discrimination index in the novel location recognition test (L), and their time spent self-grooming and bouts of self-grooming (M). n = 10 mice per group. Data represent mean ± SEM. P-values were determined by two-way ANOVA with Tukey’s multiple comparisons test in (A–D, F), repeated-measures ANOVA followed by Bonferroni’s post hoc analysis in (E), and two-tailed unpaired Student t-test in (I–M). *p < 0.05, **p < 0.01, ns: not significant. Interaction F(1,52) = 7.382, p = 0.0089 in A; Interaction F(1,52) = 0.2293, p = 0.6340 in B [file pbio.3003047.s005.tif]

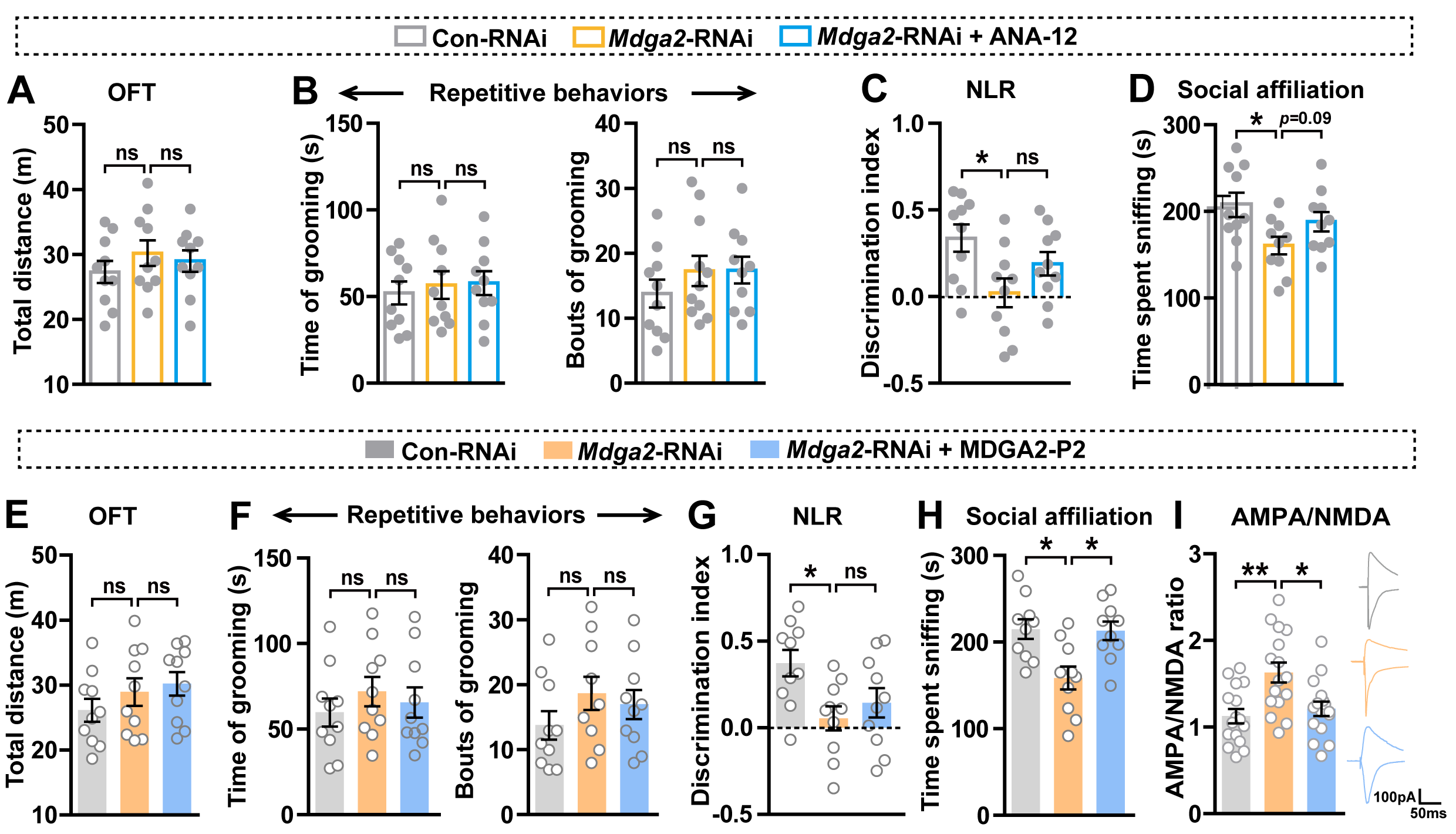

Supplement: S6 Fig — (A–I) Mice were subjected to hippocampal infection with AAVs, and then treated with ANA-12 (A–D) or MDGA2-P2 peptide (E–I). Treated mice were analyzed for their total travel distance and time spent in the center in the open field test (A, E), their time spent self-grooming and bouts of self-grooming (B, F), their discrimination index in the novel location recognition (NLR) test (C, G), and their time spent sniffing a reference mouse in the social affiliation test (D, H). n = 10 mice per group. AMPAR- and NMDAR-mediated EPSCs were recorded and their ratios were calculated for comparison (I). n = 16 cells from 3 mice per group. Data represent mean ± SEM. P-values were determined by one-way ANOVA with Tukey’s multiple comparisons test. *p < 0.05, ns: not significant. The data underlying this figure can be found in S1 Data, specifically in the sheet labeled “Supplementary Figure 6”. (TIF) [file pbio.3003047.s006.tif]

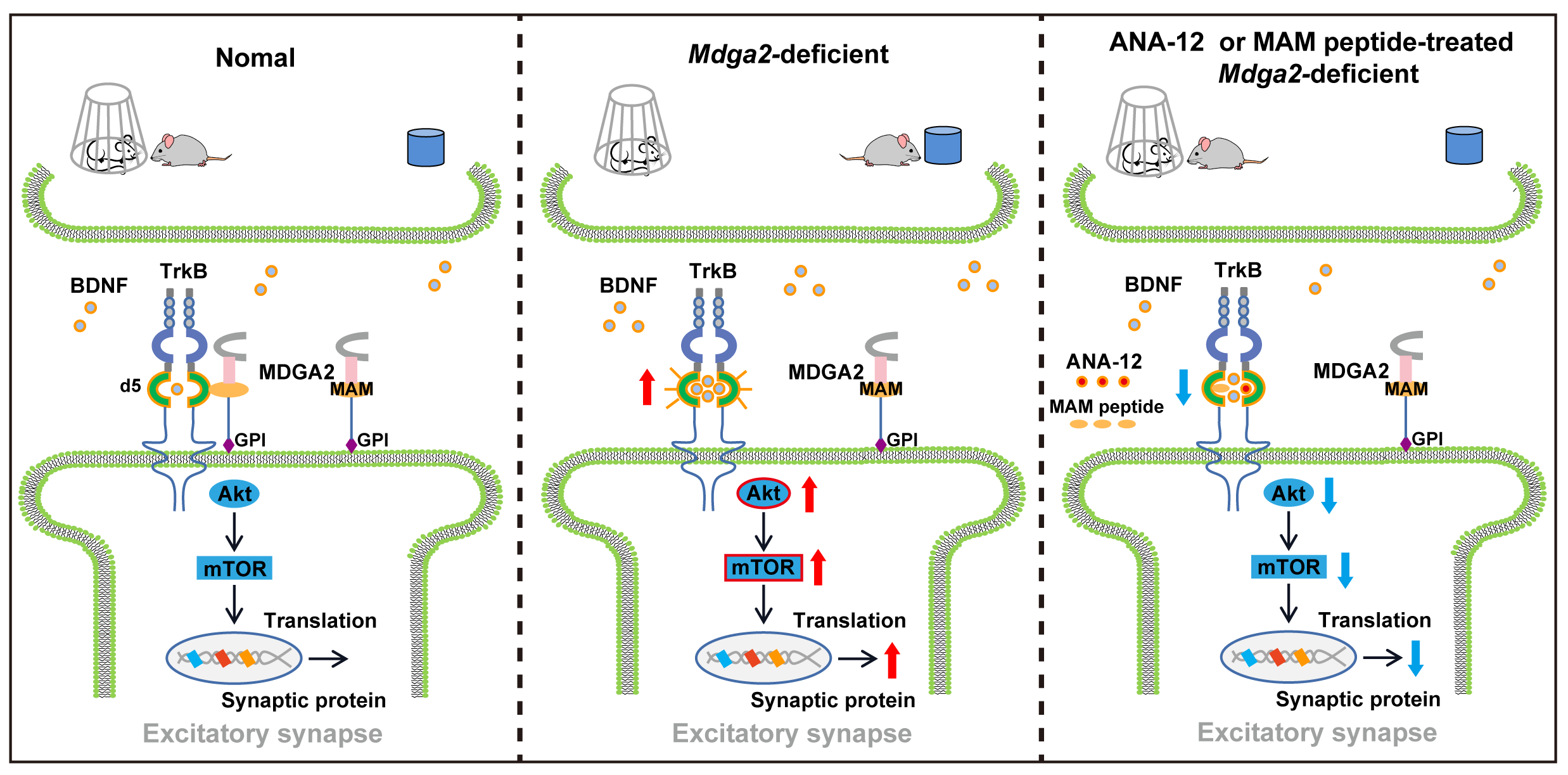

Supplement: S7 Fig — Under physiological conditions, the MAM domain of MDGA2 interacts with the d5 domain of TrkB to suppress BDNF-TrkB binding. Therefore, MDGA2 keeps the BDNF/TrkB signaling at bay for maintaining a normal Akt/mTOR signaling and downstream synaptic protein translation. MDGA2 deficiency in mice and mutations in ASD patients lead to excessive activation of the BDNF/TrkB/Akt/mTOR signaling and elevated synaptic protein production, resulting in increased excitatory synapse transmission and ASD-associated behavioral phenotypes. Inhibiting TrkB activity by ANA-12 or MDGA2 MAM domain peptide can reduce the aberrantly activated Akt/mTOR signaling, elevated synaptic protein levels and excitatory synapse transmission, and thus ASD-associated social deficits in Mdga2-deficient mice. (TIF) [file pbio.3003047.s007.tif]
