# Supplementary material for: Mdga2 deficiency leads to an aberrant activation of BDNF/TrkB signaling that underlies autism-relevant synaptic and behavioral changes in mice
Source: PLoS Biol. 2025 Apr 1;23(4):e3003047. doi: 10.1371/journal.pbio.3003047 (PMC11960969; doi:10.1371/journal.pbio.3003047)

Raw images for figures

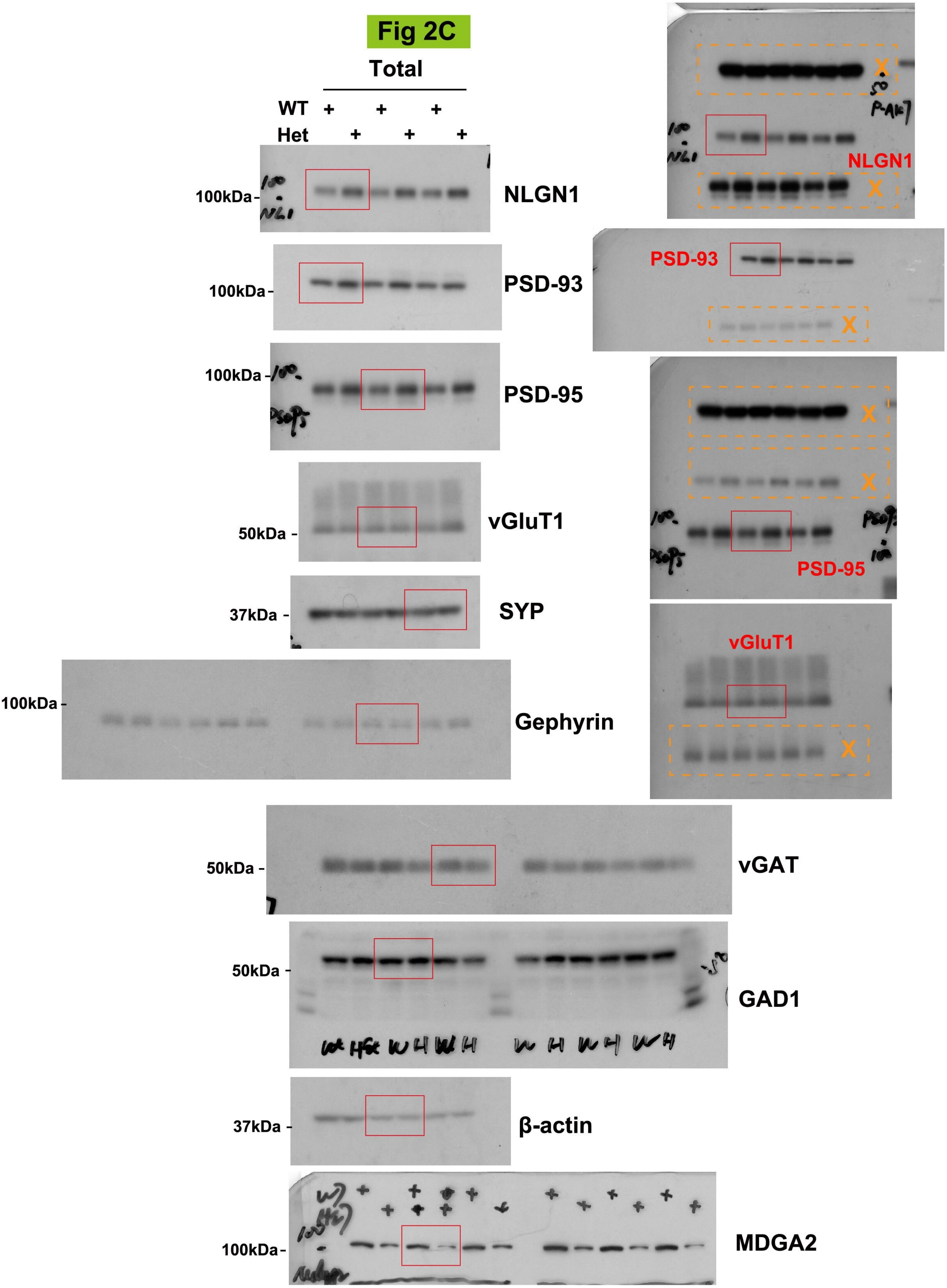

Fig 2D

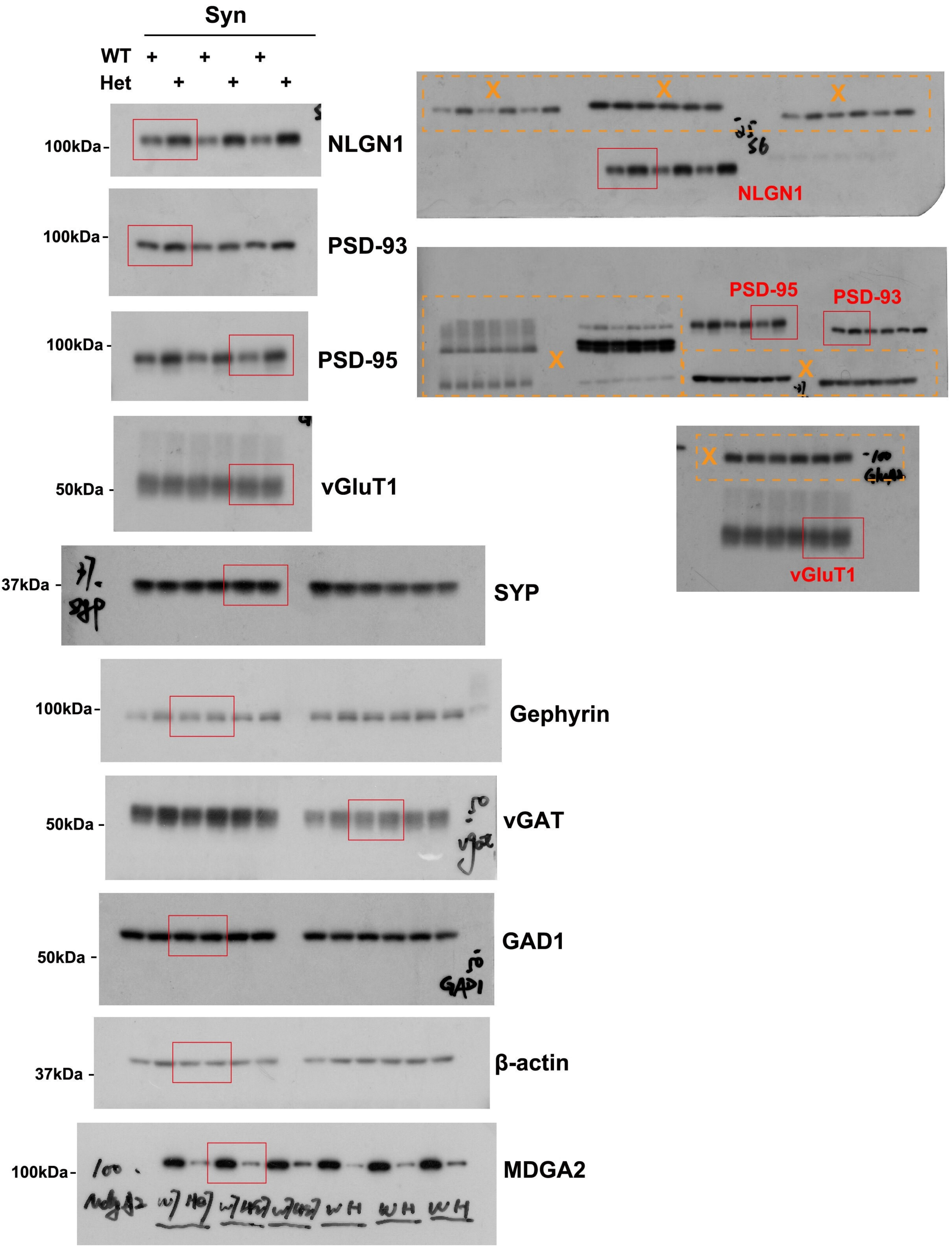

Syn
WT + + +
Het + + +
100kDa
NLGN1
100kDa
PSD-93
100kDa
PSD-95
50kDa
vGluT1
37kDa
SYP
100kDa
Gephyrin
50kDa
vGAT
50kDa
GAD1
37kDa
β-actin
100kDa
MDGA2
NLGN1
PSD-95
PSD-93
vGluT1

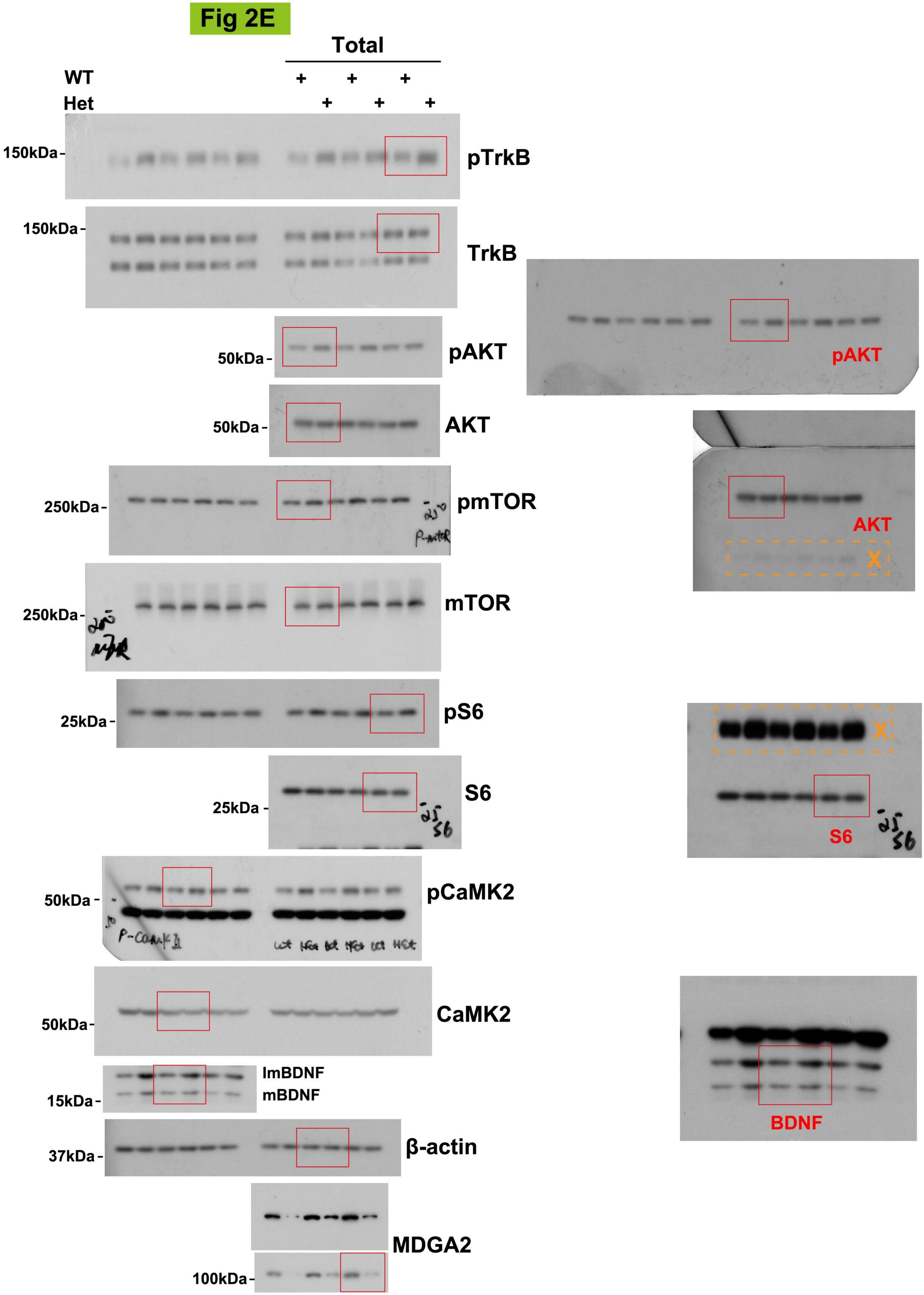
Fig 2E
Total
WT
Het
150kDa
pTrkB
150kDa
TrkB
50kDa
pAKT
50kDa
AKT
250kDa
pmTOR
250kDa
mTOR
25kDa
pS6
25kDa
S6
50kDa
pCaMK2
50kDa
CaMK2
lmBDNF
mBDNF
15kDa
β-actin
37kDa
MDGA2
100kDa
pAKT
AKT
X
X
S6
BDNF

Fig 2H

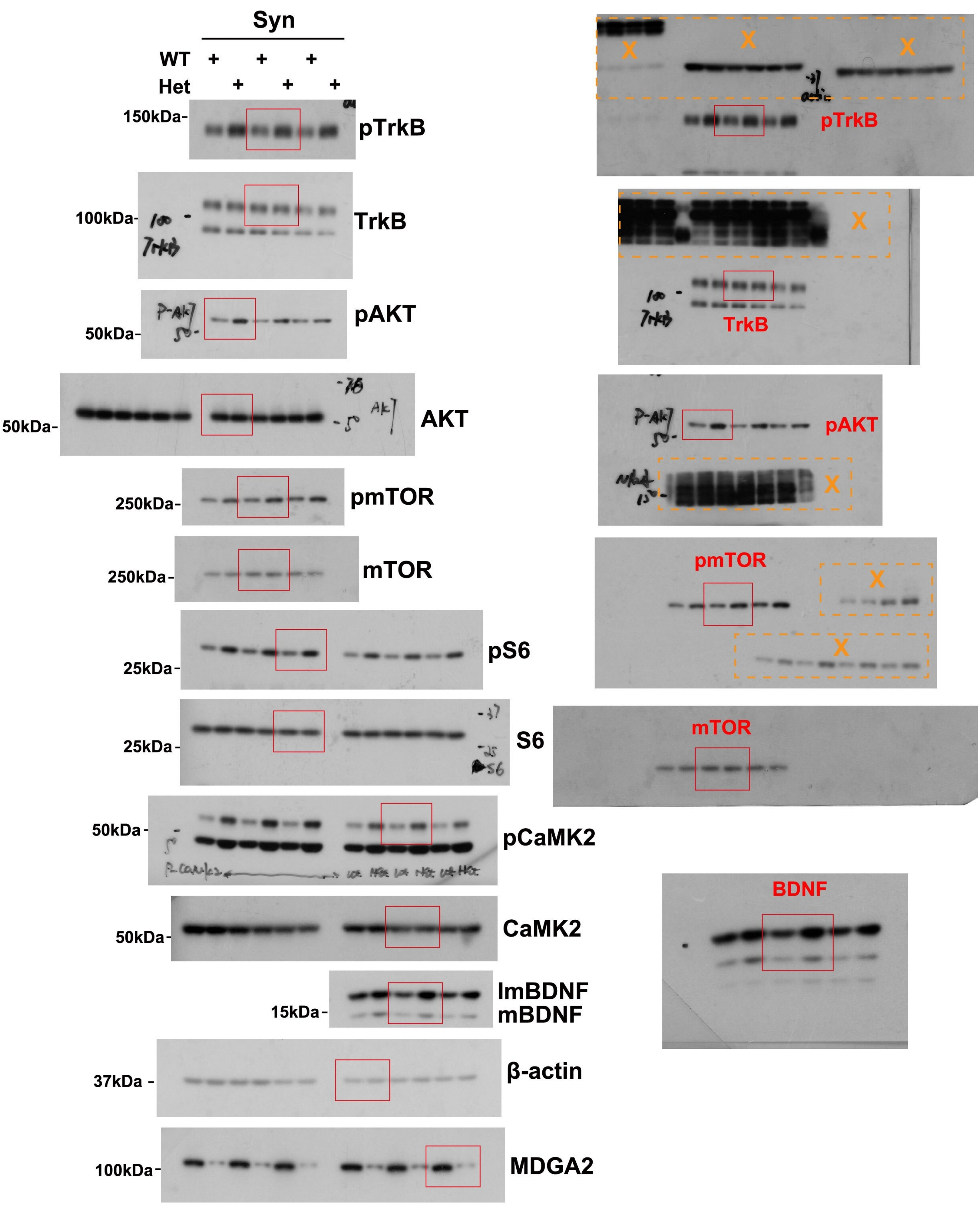
Syn
WT + + +
Het + + +
150kDa- pTrkB
100kDa- TrkB
50kDa- pAKT
50kDa- AKT
250kDa- pmTOR
250kDa- mTOR
25kDa- pS6
25kDa- S6
50kDa- pCaMK2
50kDa- CaMK2
15kDa- lmBDNF
mBDNF
37kDa - β-actin
100kDa- MDGA2
X
pTrkB
TrkB
pAKT
pmTOR
mTOR
BDNF

Fig 3A

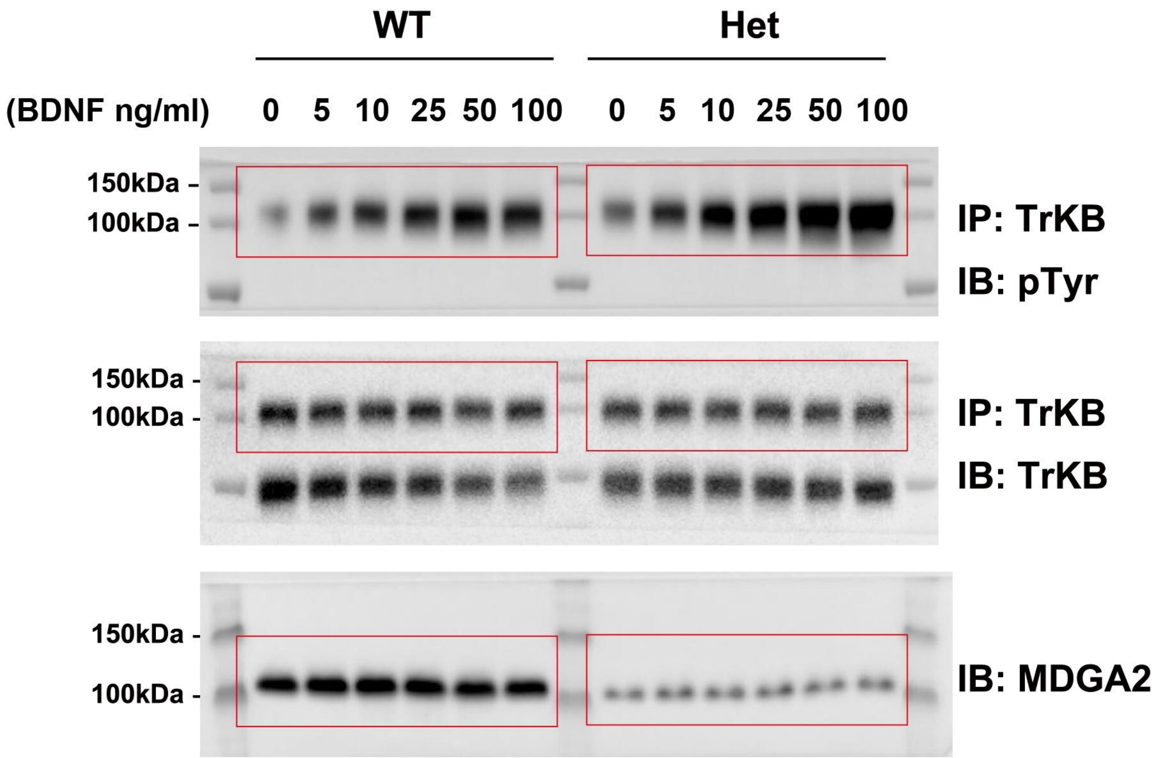

Fig 3B

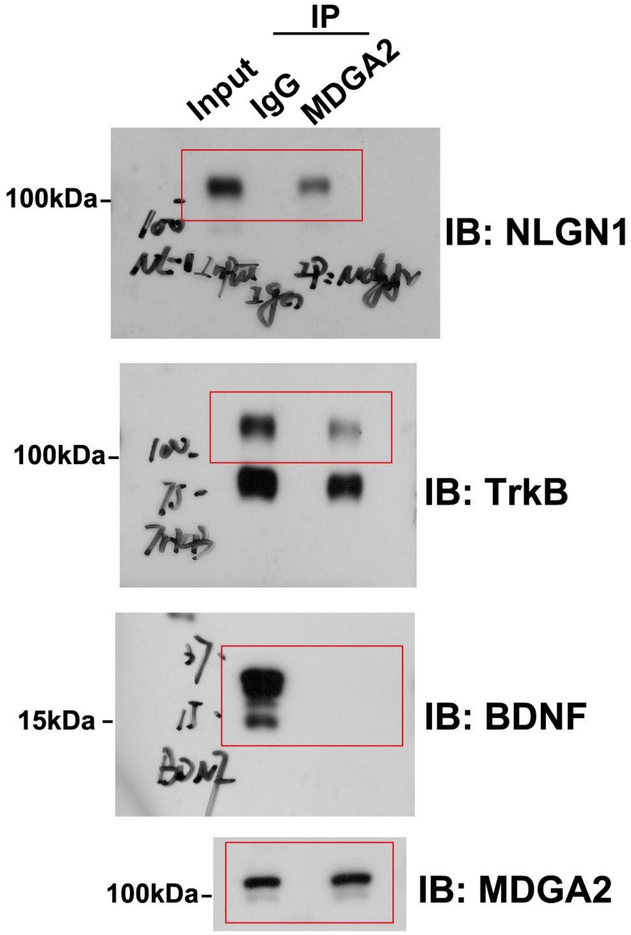

Fig 3F

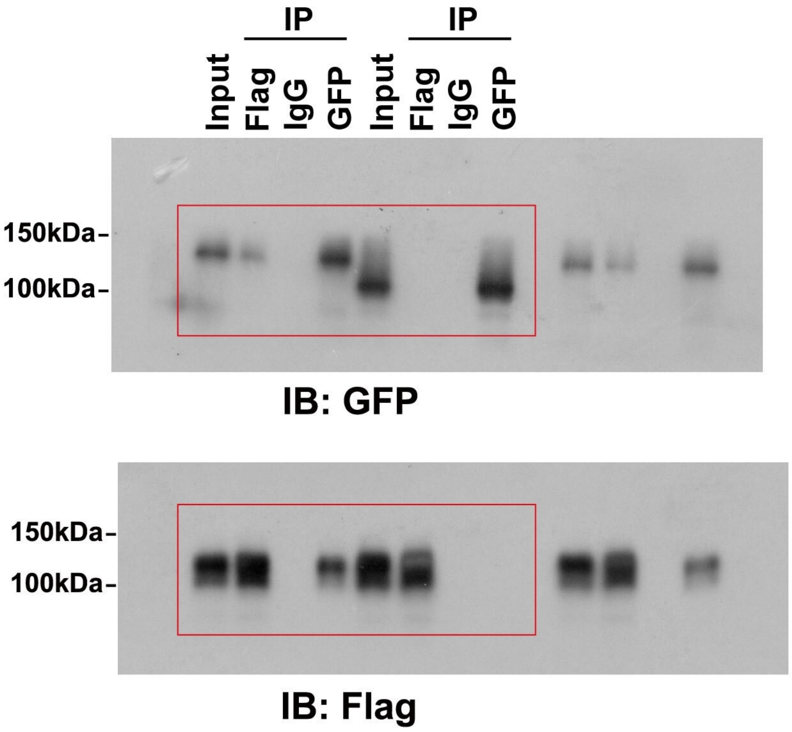

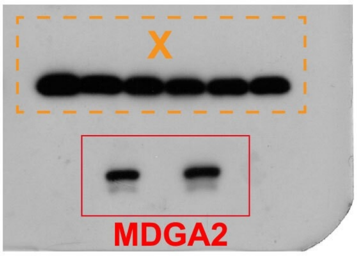

Fig 3H

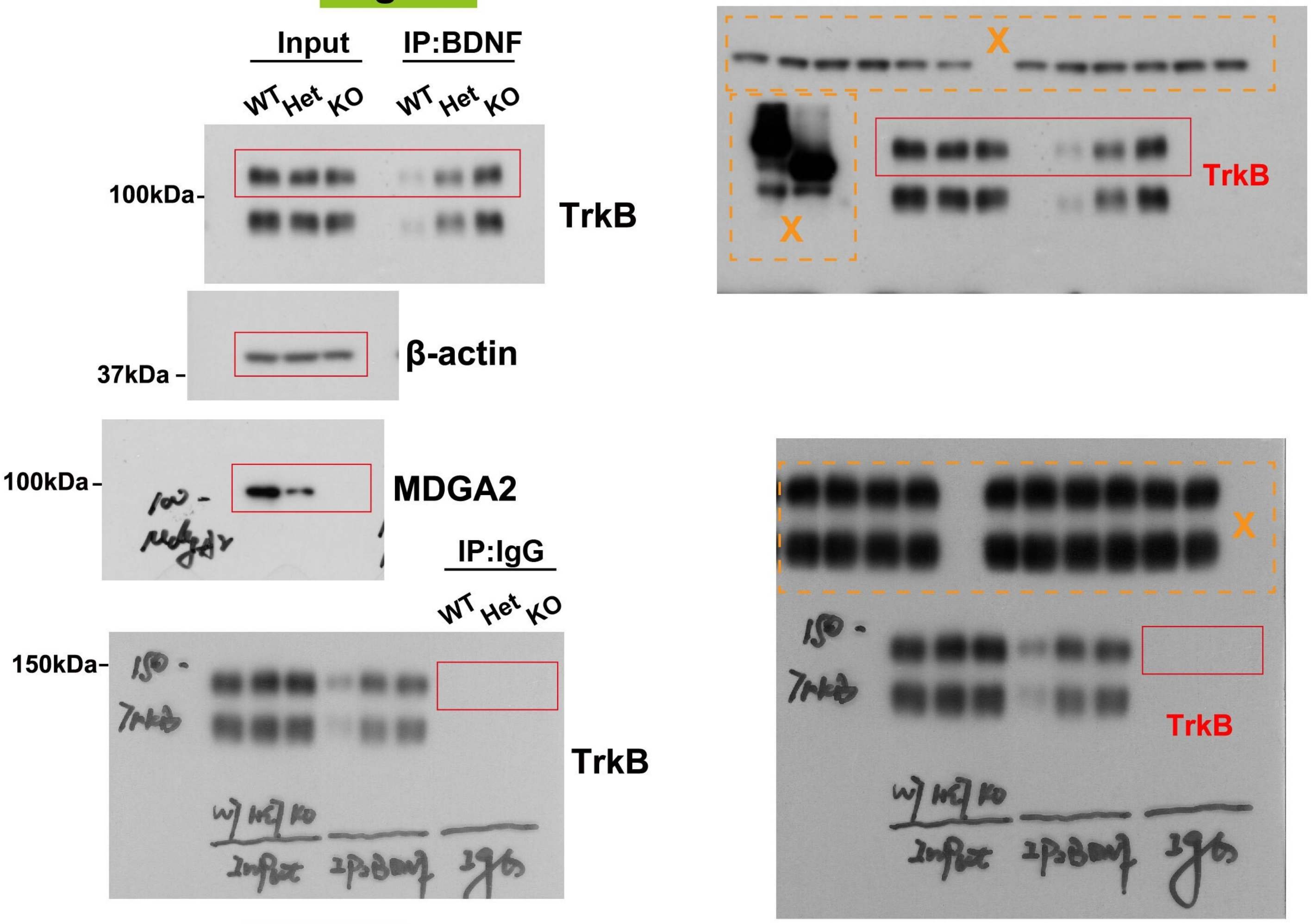
Input
IP:BDNF
WT
Het
KO
WT
Het
KO
100kDa
TrkB
37kDa
β-actin
100kDa
MDGA2
IP:IgG
WT
Het
KO
150kDa
TrkB
TrkB
TrkB

Fig 3I

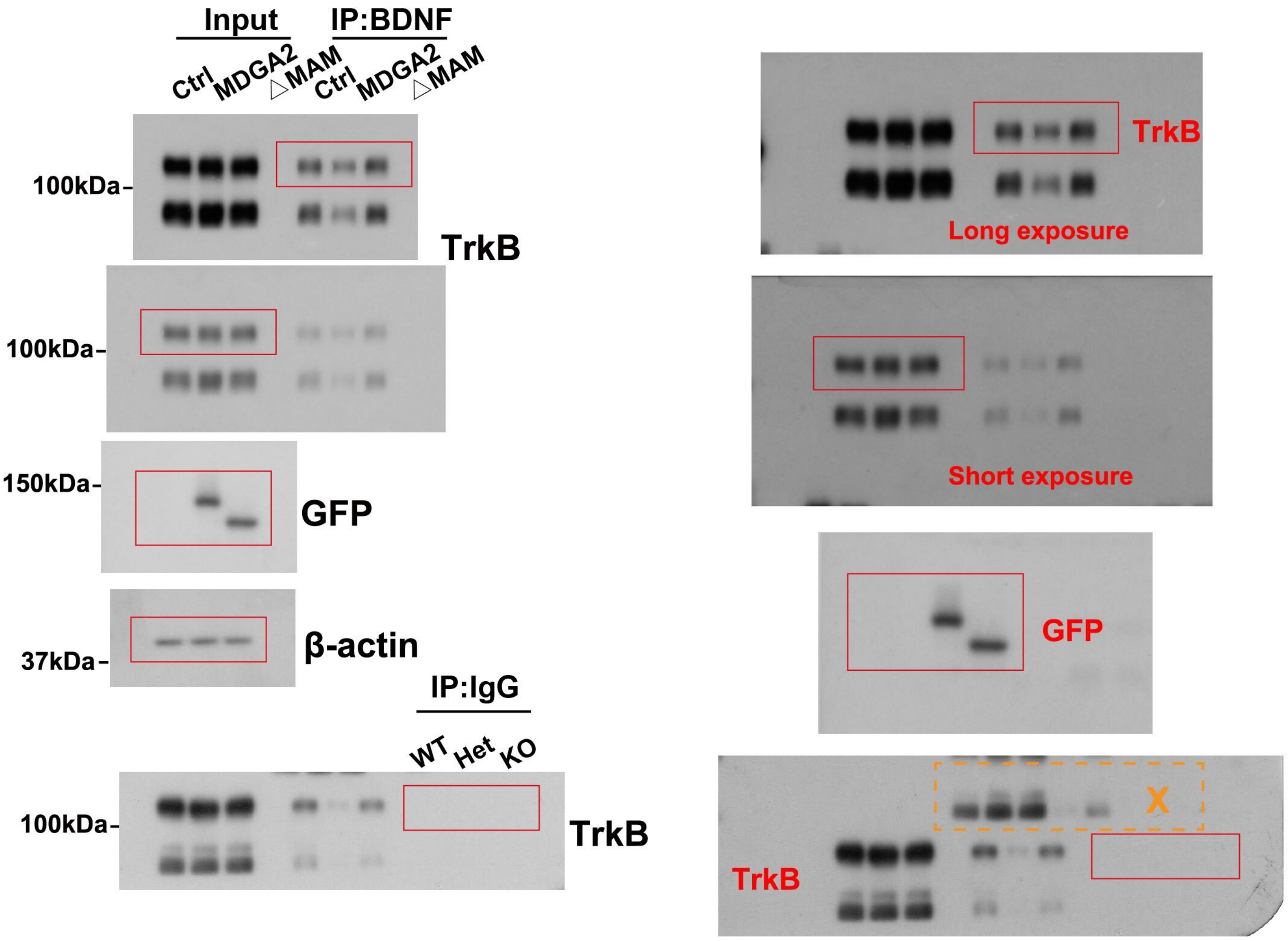
Input
IP:BDNF
Ctrl
MDGA2
△MAM
Ctrl
MDGA2
△MAM
100kDa
TrkB
100kDa
150kDa
GFP
37kDa
β-actin
IP:IgG
WT
Het
KO
100kDa
TrkB
TrkB
Long exposure
Short exposure
GFP
TrkB

Fig 3J

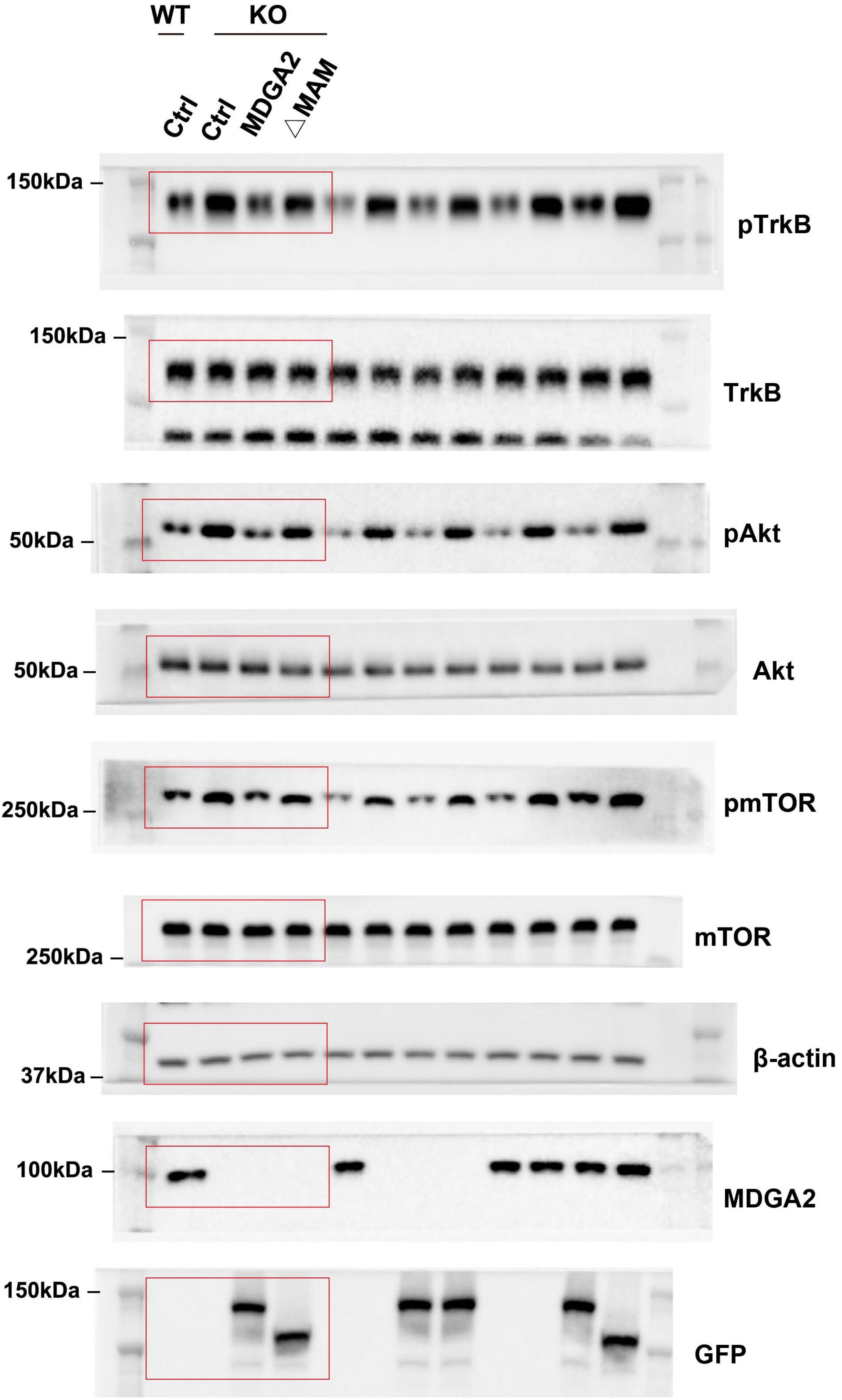

WT
KO
Ctrl
Ctrl
MDGA2
▽MAM
150kDa
pTrkB
150kDa
TrkB
50kDa
pAkt
50kDa
Akt
250kDa
pmTOR
250kDa
mTOR
37kDa
β-actin
100kDa
MDGA2
150kDa
GFP

Fig 4B

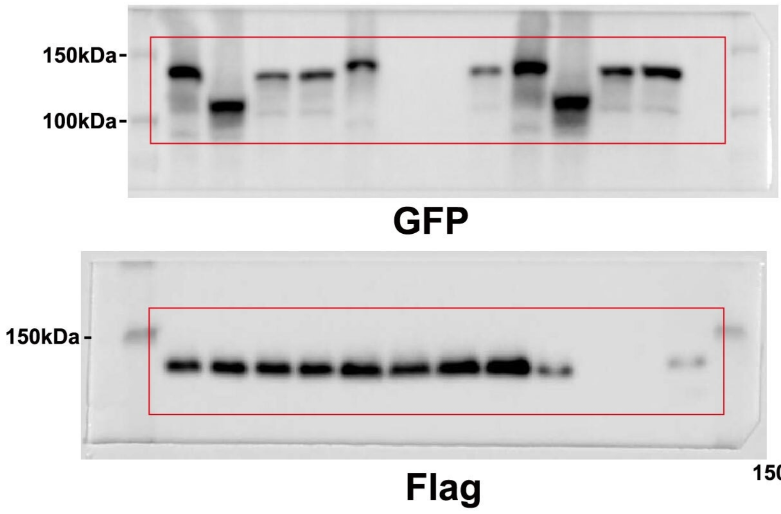

Fig 4H

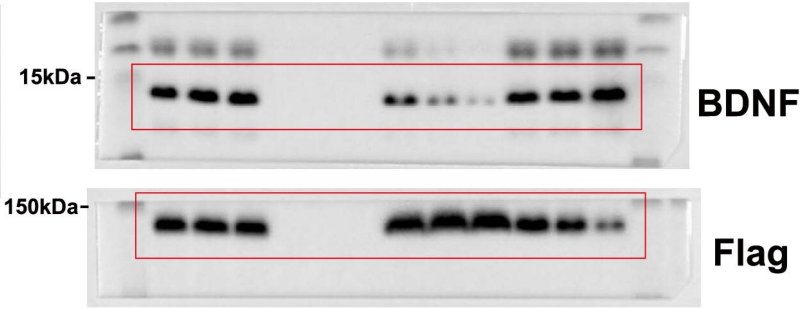

Fig 4N

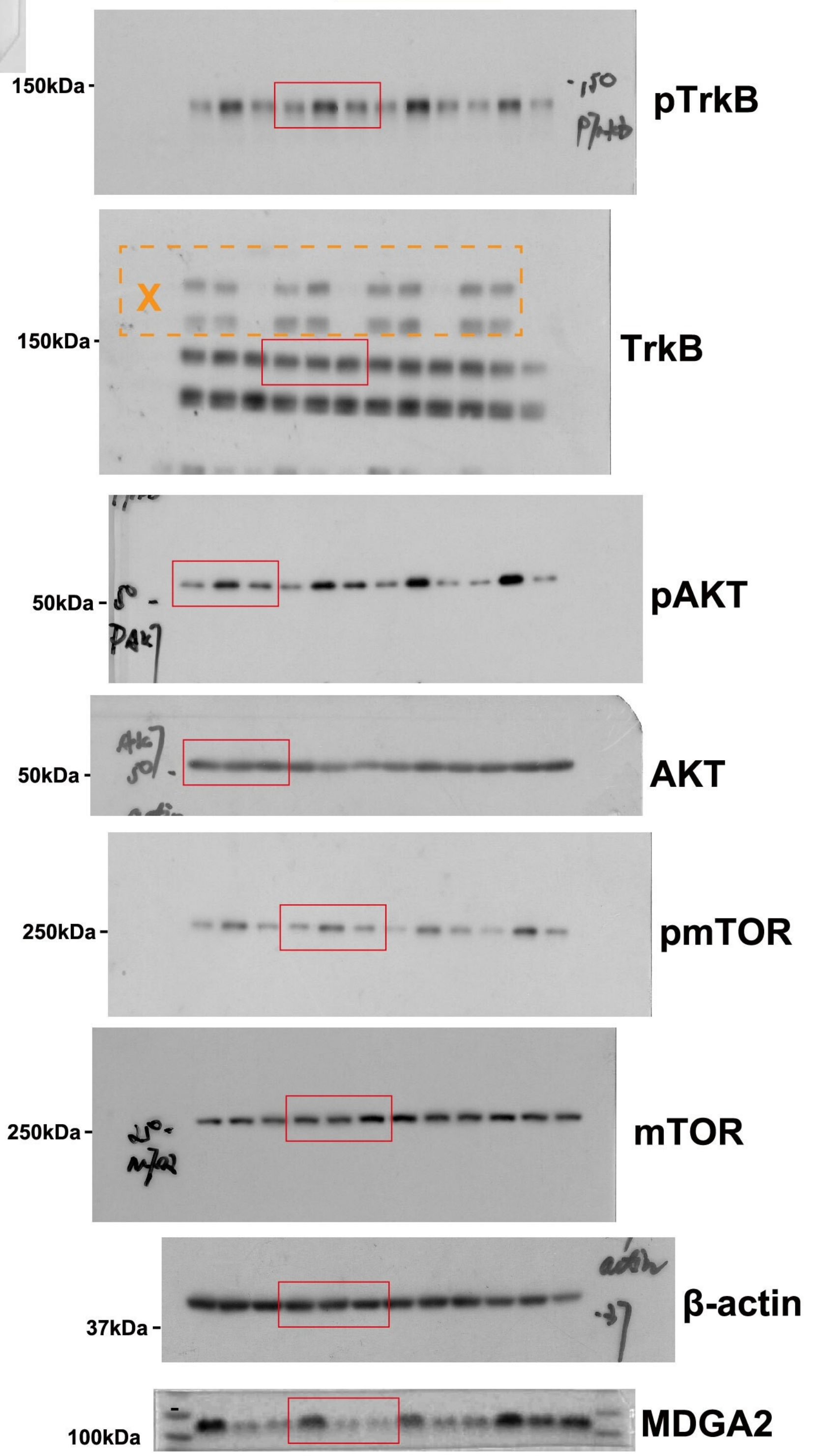

Fig 4D

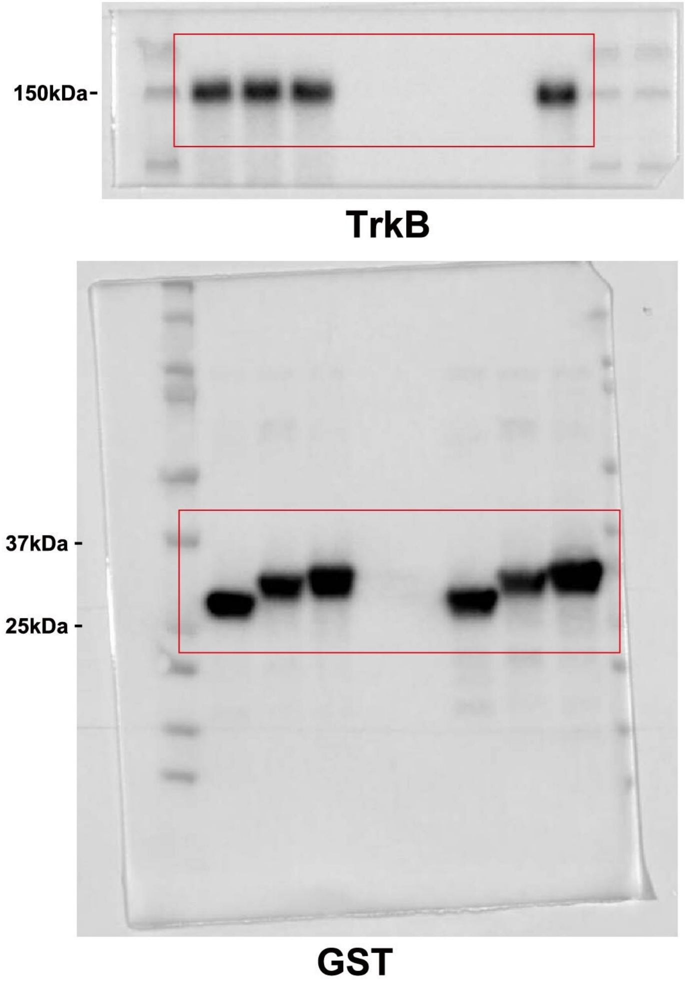

Fig 4O

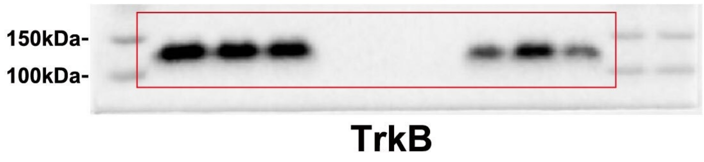

Fig 4E

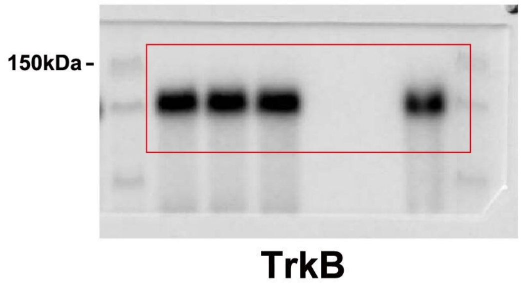

Fig 5G

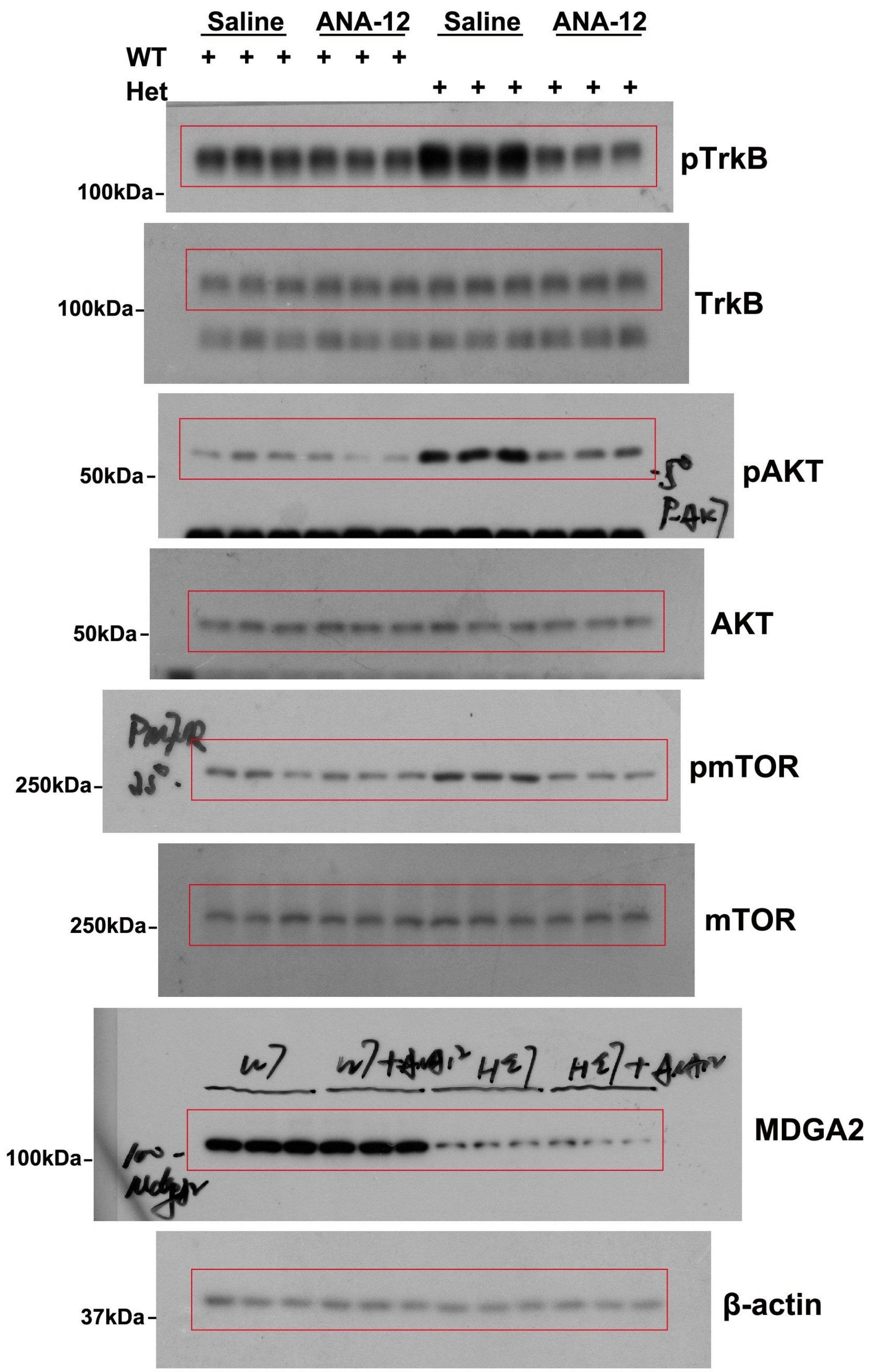

Saline
ANA-12
Saline
ANA-12
WT + + + + + +
Het + + + + + +
pTrkB
100kDa-
TrkB
100kDa-
pAKT
50kDa-
AKT
50kDa-
pmTOR
250kDa-
mTOR
250kDa-
MDGA2
100kDa-
β-actin
37kDa-

Fig 6C

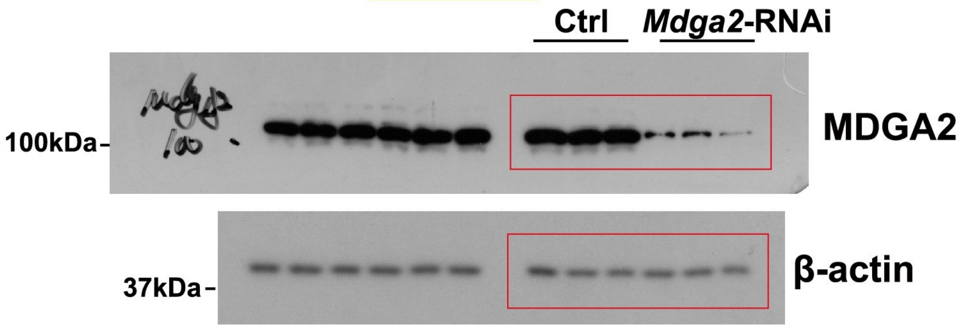

Ctrl
Mdga2-RNAi
MDGA2
100kDa-
β-actin
37kDa-

Fig 6E

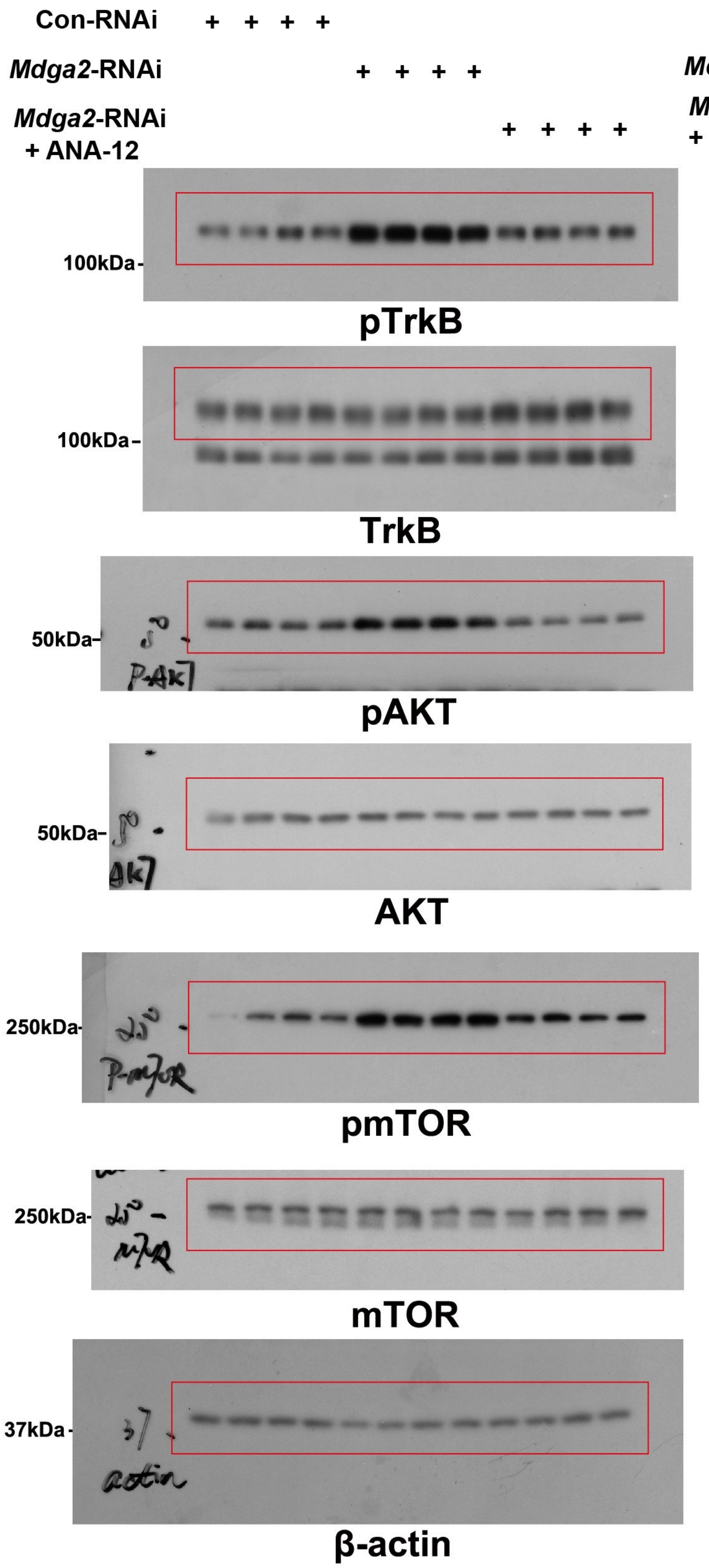
Con-RNAi
Mdga2-RNAi
Mdga2-RNAi + ANA-12
100kDa
pTrkB
100kDa
TrkB
50kDa
pAKT
50kDa
AKT
250kDa
pmTOR
250kDa
mTOR
37kDa
β-actin

Fig 6H

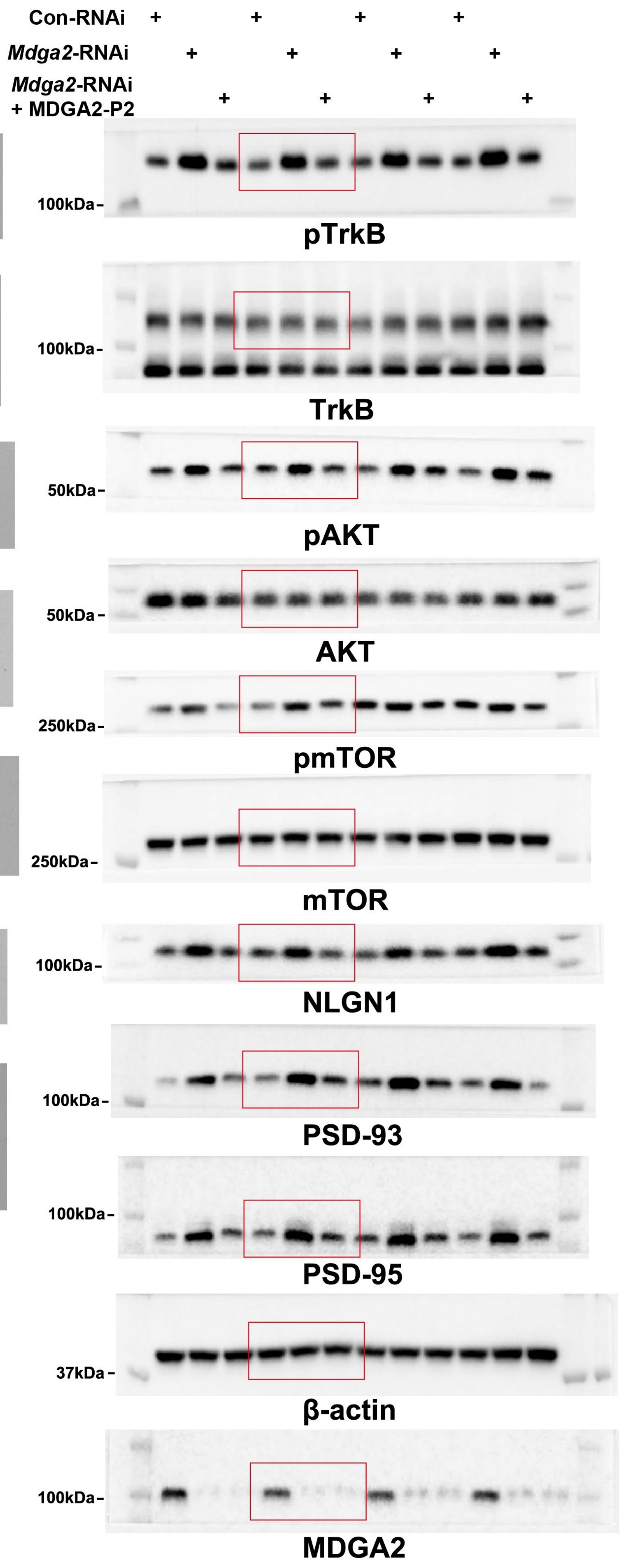
Con-RNAi
Mdga2-RNAi
Mdga2-RNAi + MDGA2-P2
100kDa
pTrkB
100kDa
TrkB
50kDa
pAKT
50kDa
AKT
250kDa
pmTOR
250kDa
mTOR
100kDa
NLGN1
100kDa
PSD-93
100kDa
PSD-95
37kDa
β-actin
100kDa
MDGA2

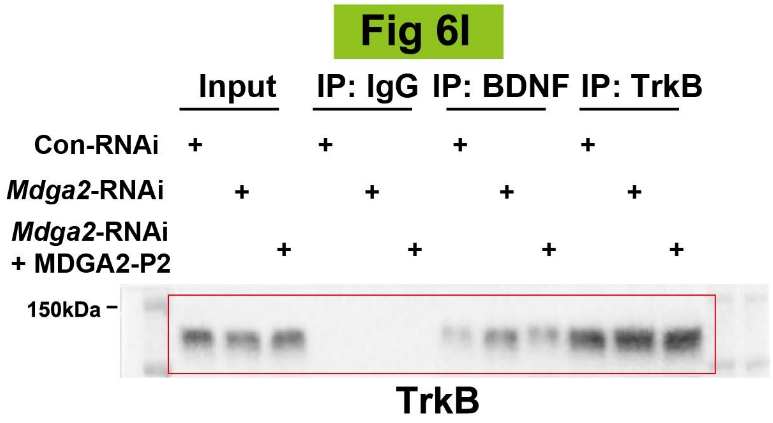
Fig 6I
Input
IP: IgG
IP: BDNF
IP: TrkB
Con-RNAi
Mdga2-RNAi
Mdga2-RNAi + MDGA2-P2
150kDa
TrkB

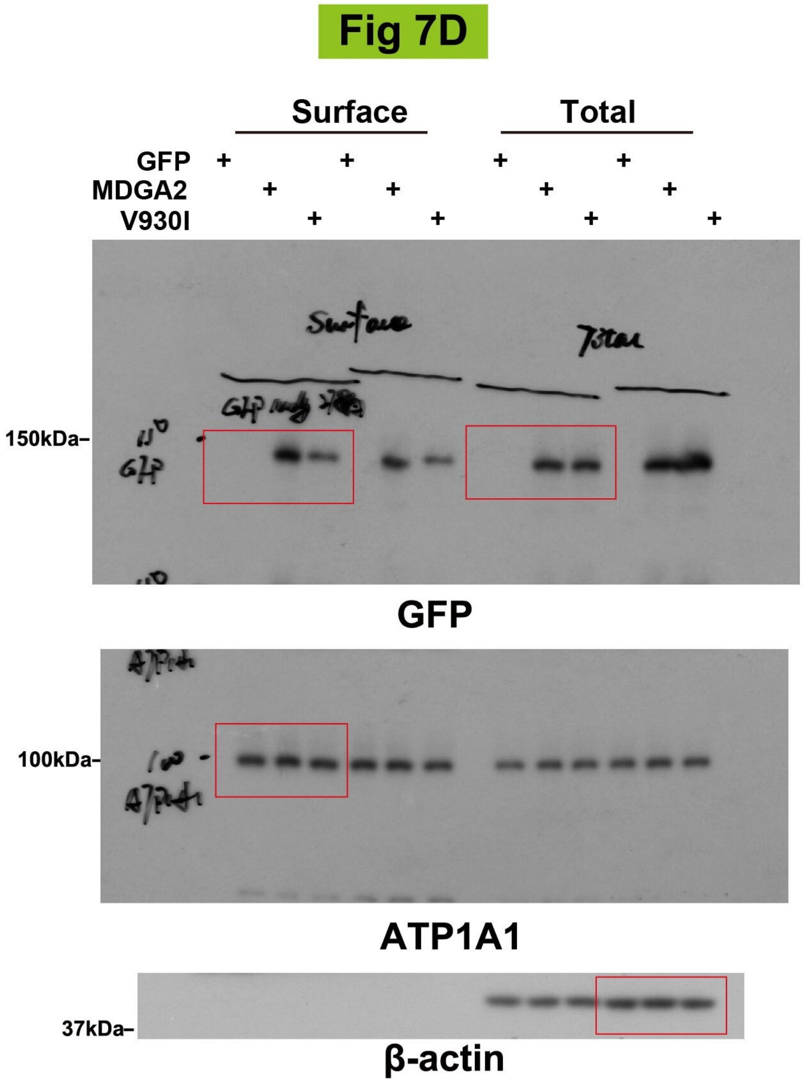
Fig 7D
Surface
Total
GFP
MDGA2
V930I
150kDa
GFP
100kDa
ATP1A1
37kDa
β-actin

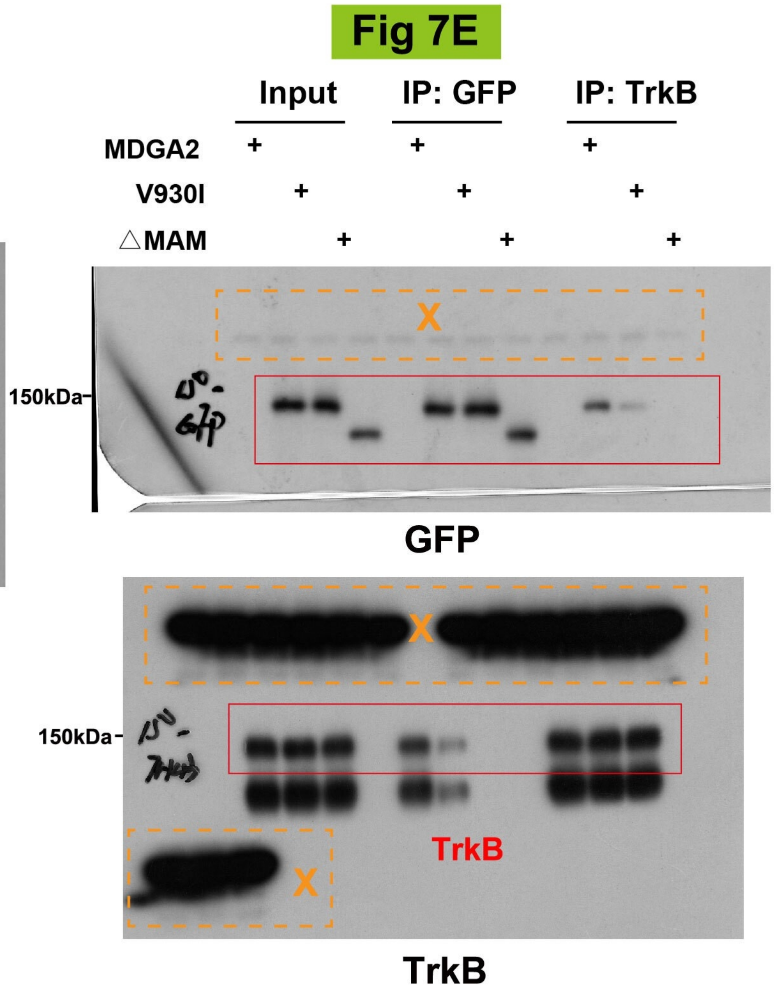
Fig 7E
Input
IP: GFP
IP: TrkB
MDGA2
V930I
△MAM
150kDa
X
GFP
150kDa
X
TrkB
X
TrkB

Fig 7F

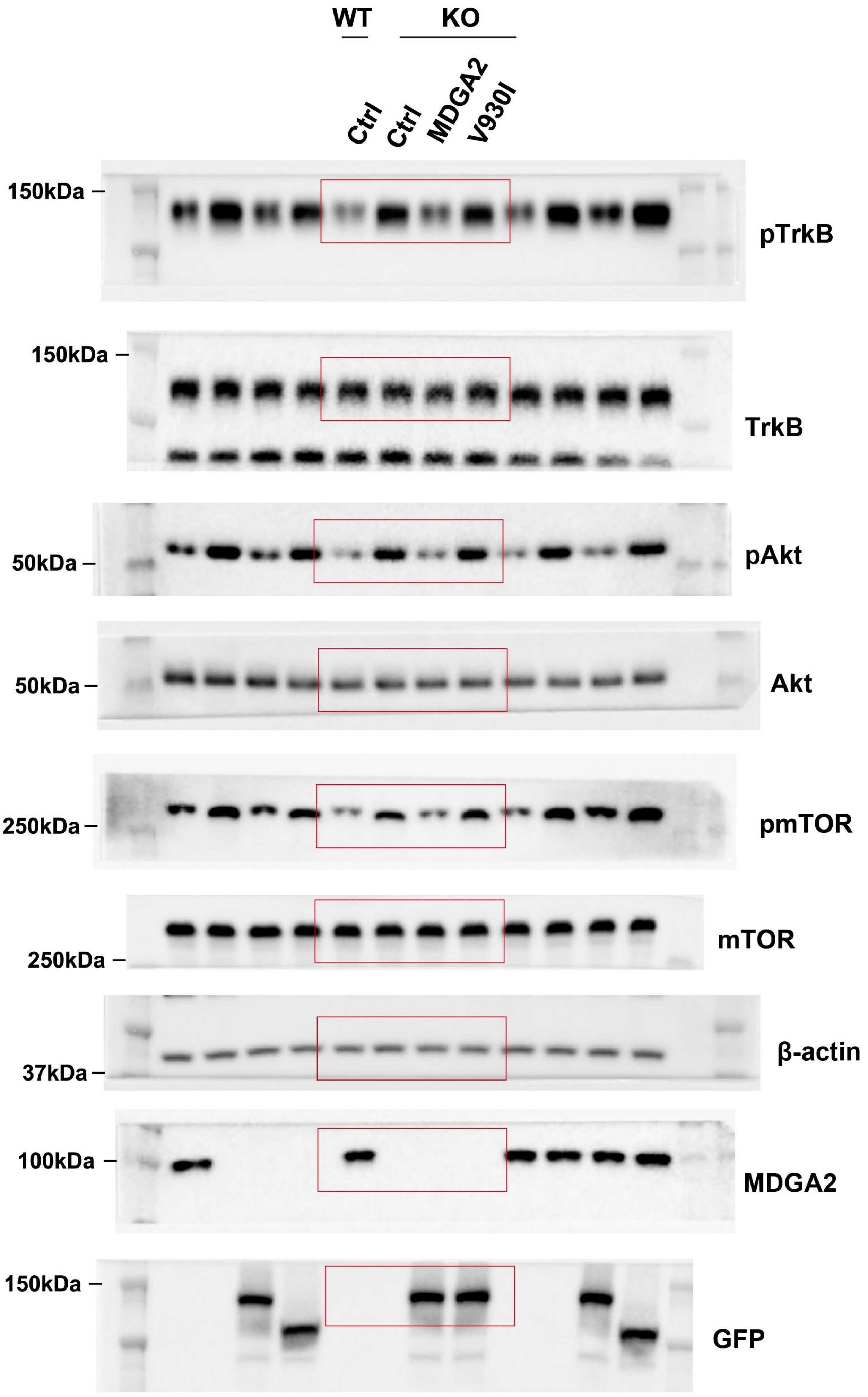
WT
KO
Ctrl
Ctrl
MDGA2
V930I
150kDa
pTrkB
150kDa
TrkB
50kDa
pAkt
50kDa
Akt
250kDa
pmTOR
mTOR
250kDa
β-actin
37kDa
100kDa
MDGA2
150kDa
GFP

Raw images for supplementary figures

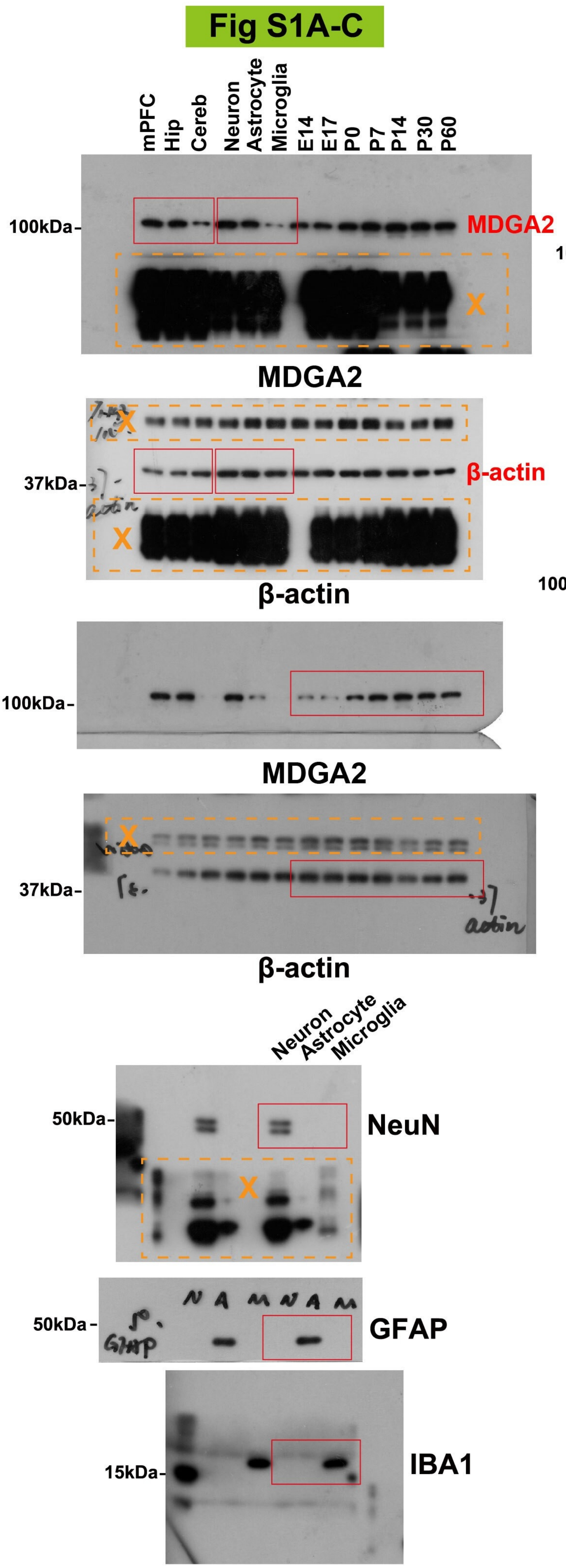

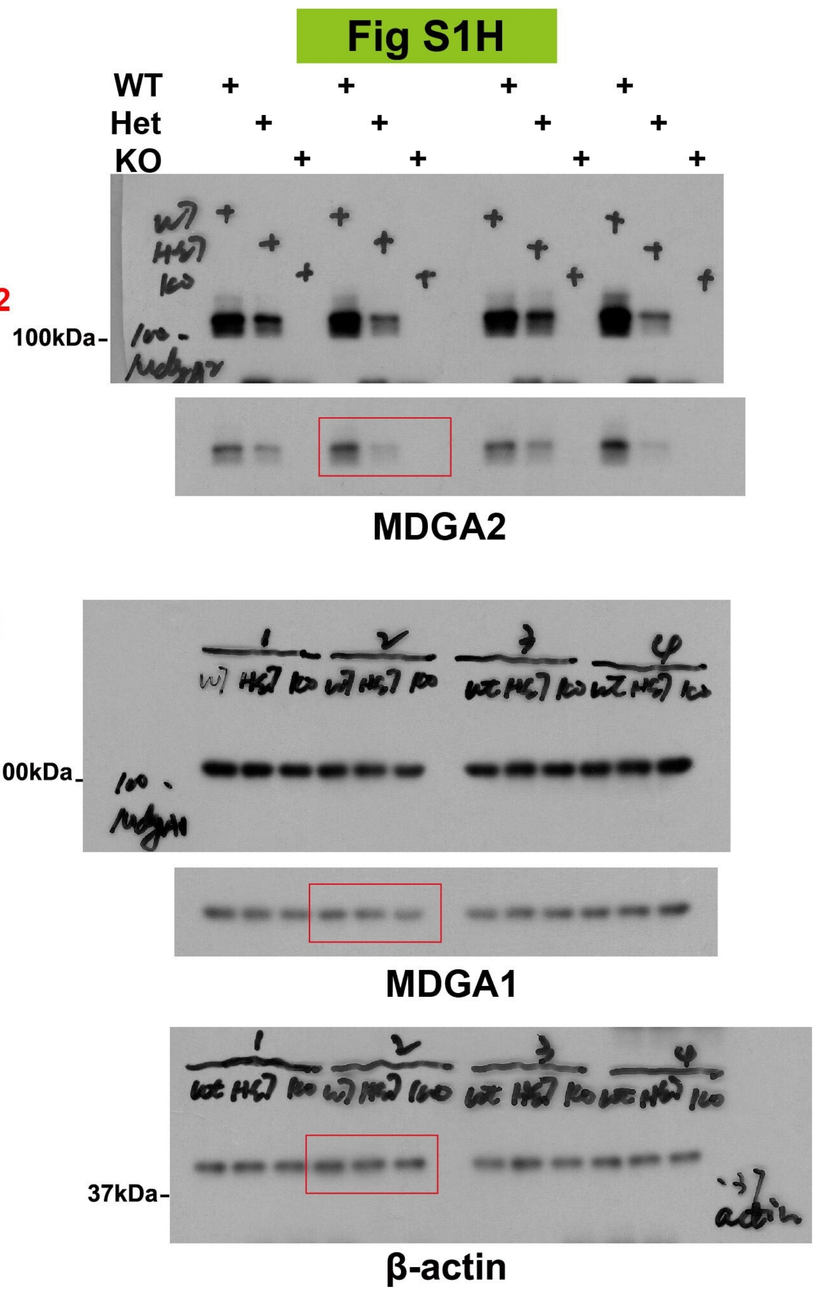

Fig S2A

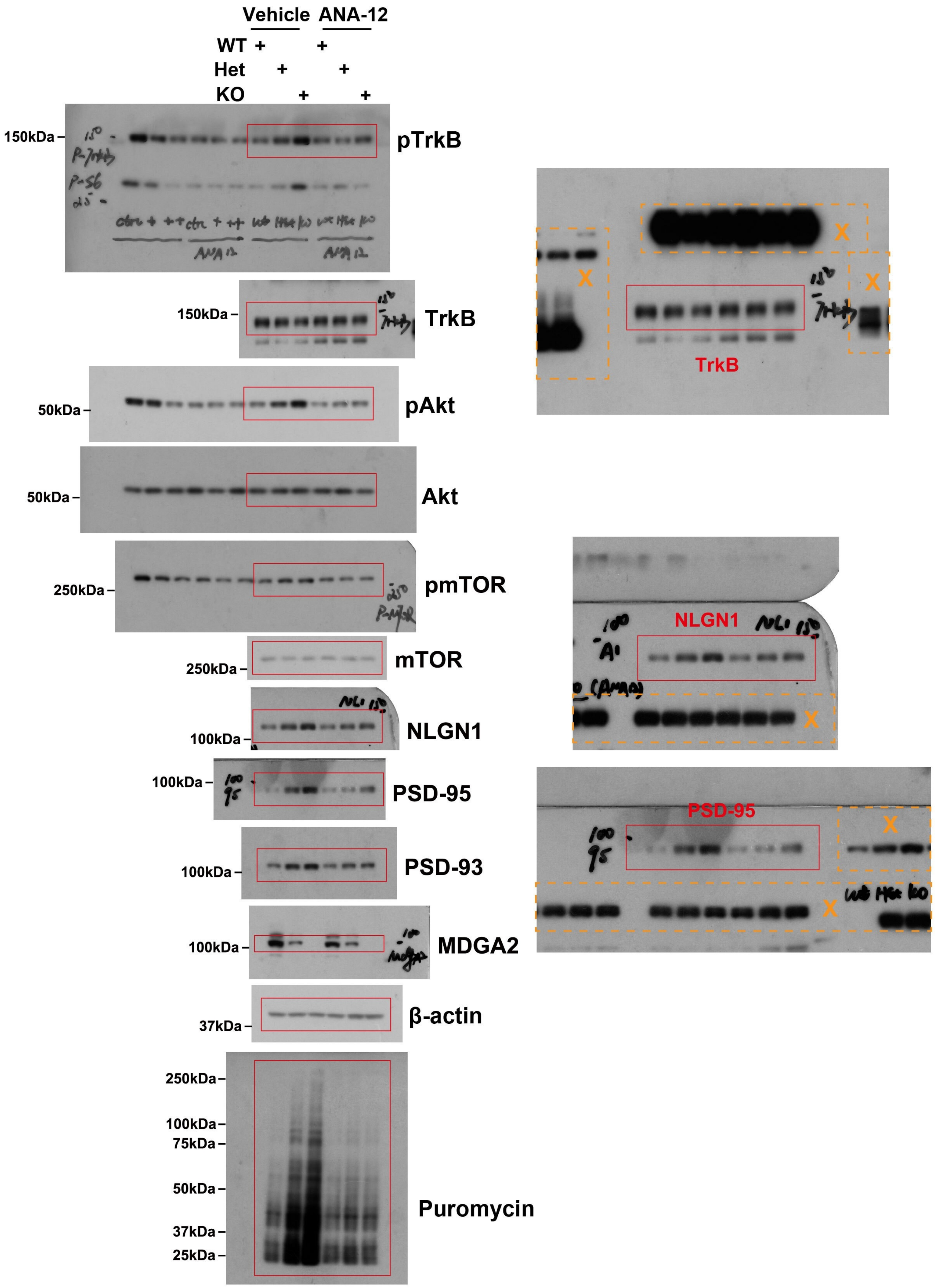

Vehicle
ANA-12
WT + +
Het + +
KO + +
150kDa
pTrkB
150kDa
TrkB
50kDa
pAkt
50kDa
Akt
250kDa
pmTOR
250kDa
mTOR
100kDa
NLGN1
100kDa
PSD-95
100kDa
PSD-93
100kDa
MDGA2
37kDa
β-actin
250kDa
100kDa
75kDa
50kDa
37kDa
25kDa
Puromycin
TrkB
X
NLGN1
X
PSD-95
X

Fig S3A

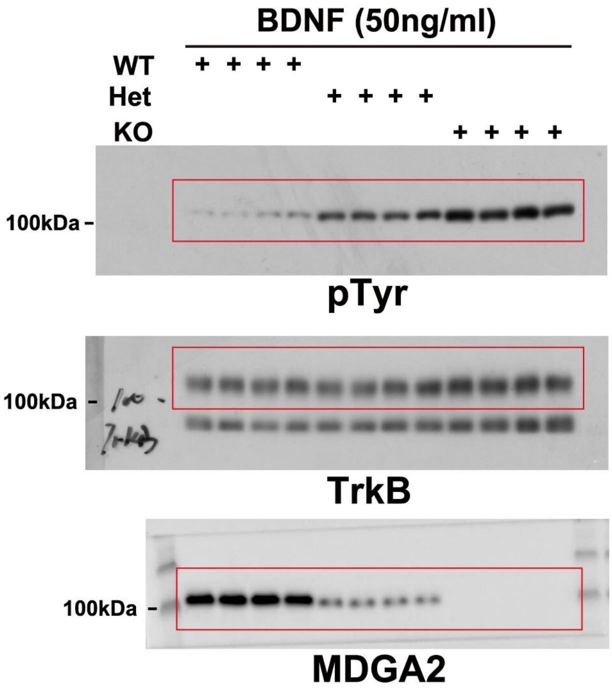

Fig S3C

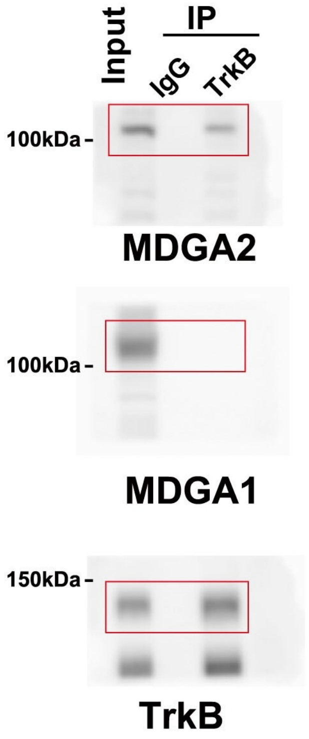

Fig S3H

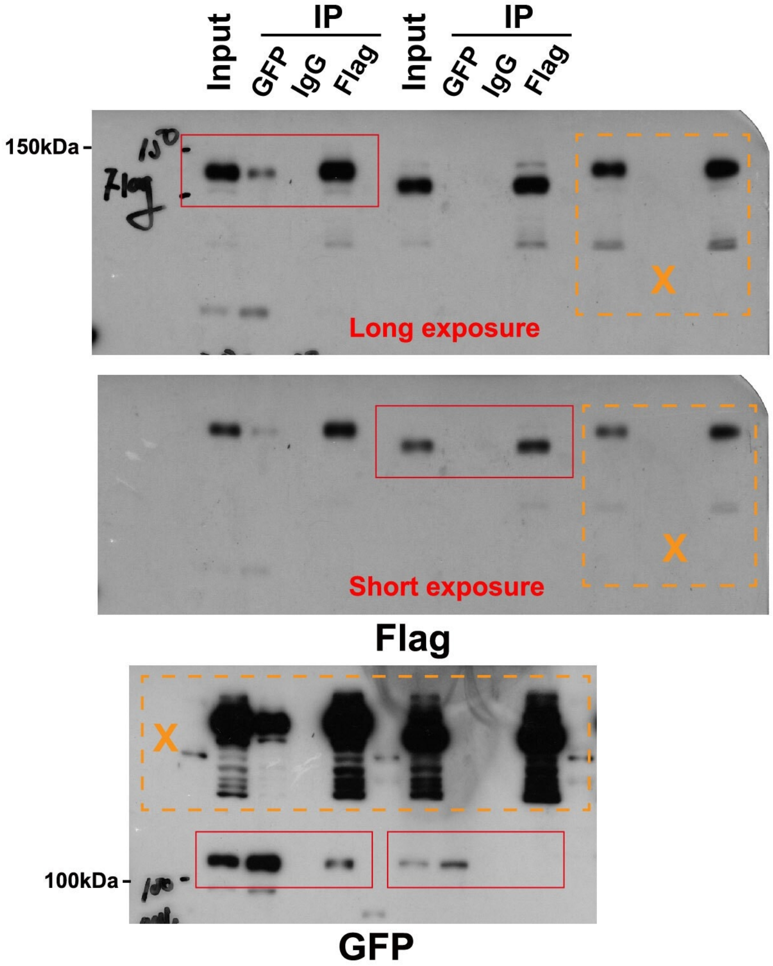

Fig S3I

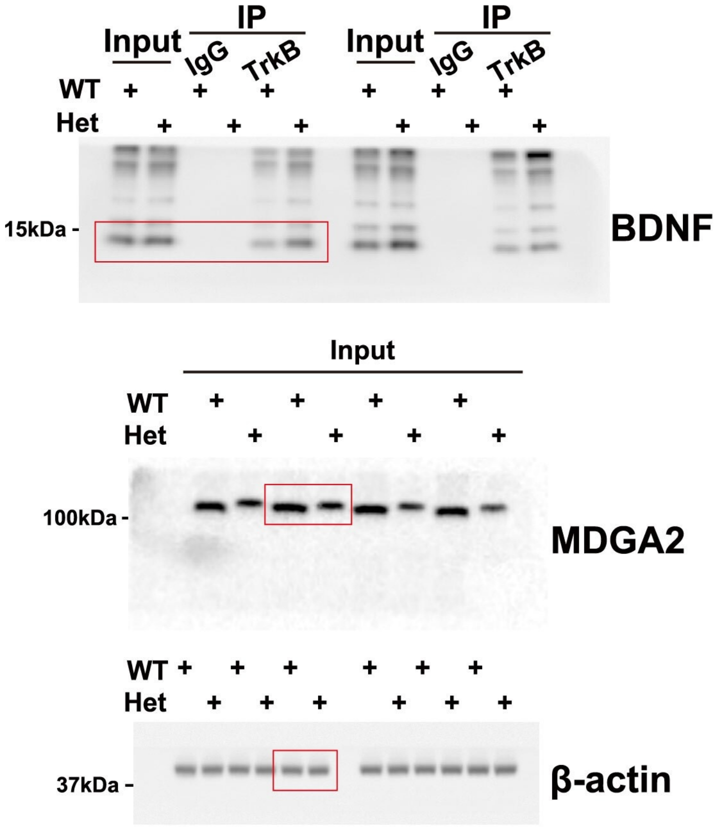

Fig S3J

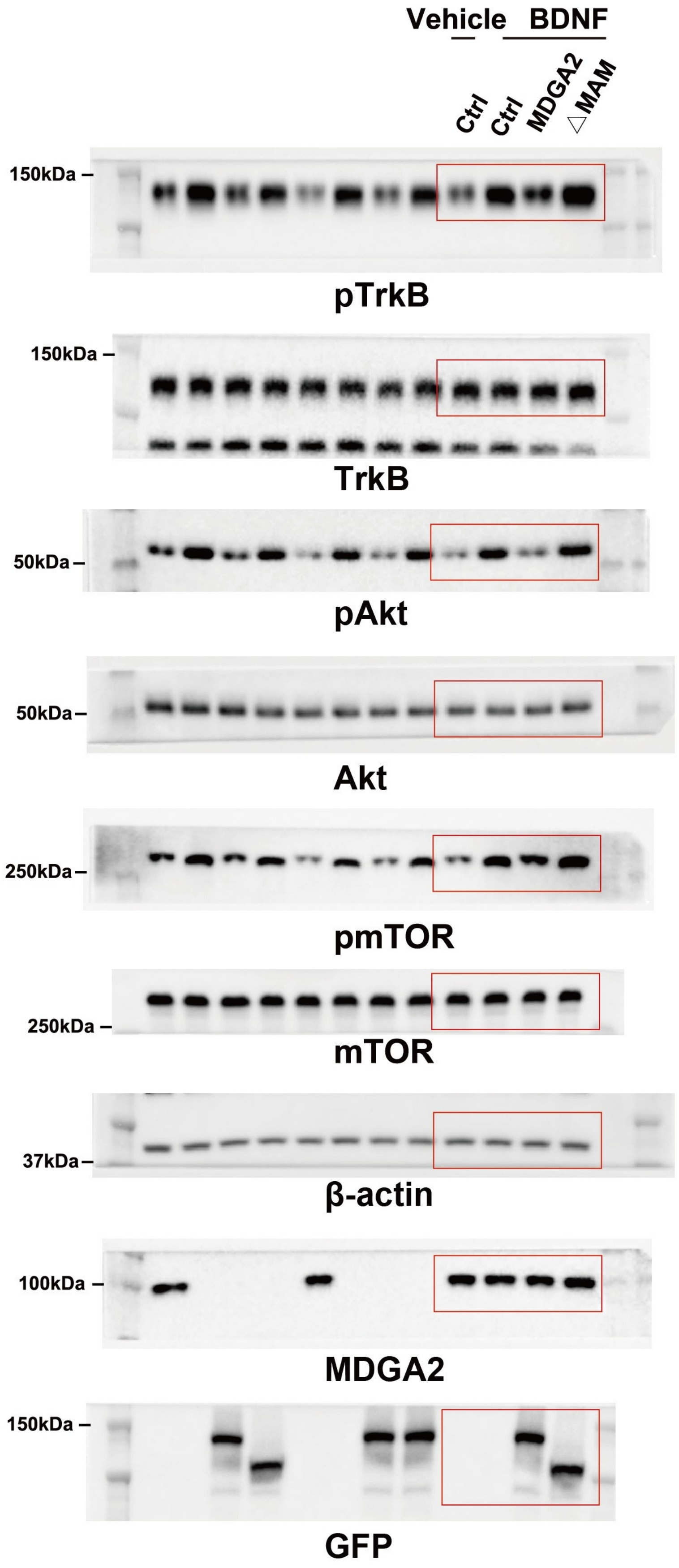

Fig S3K

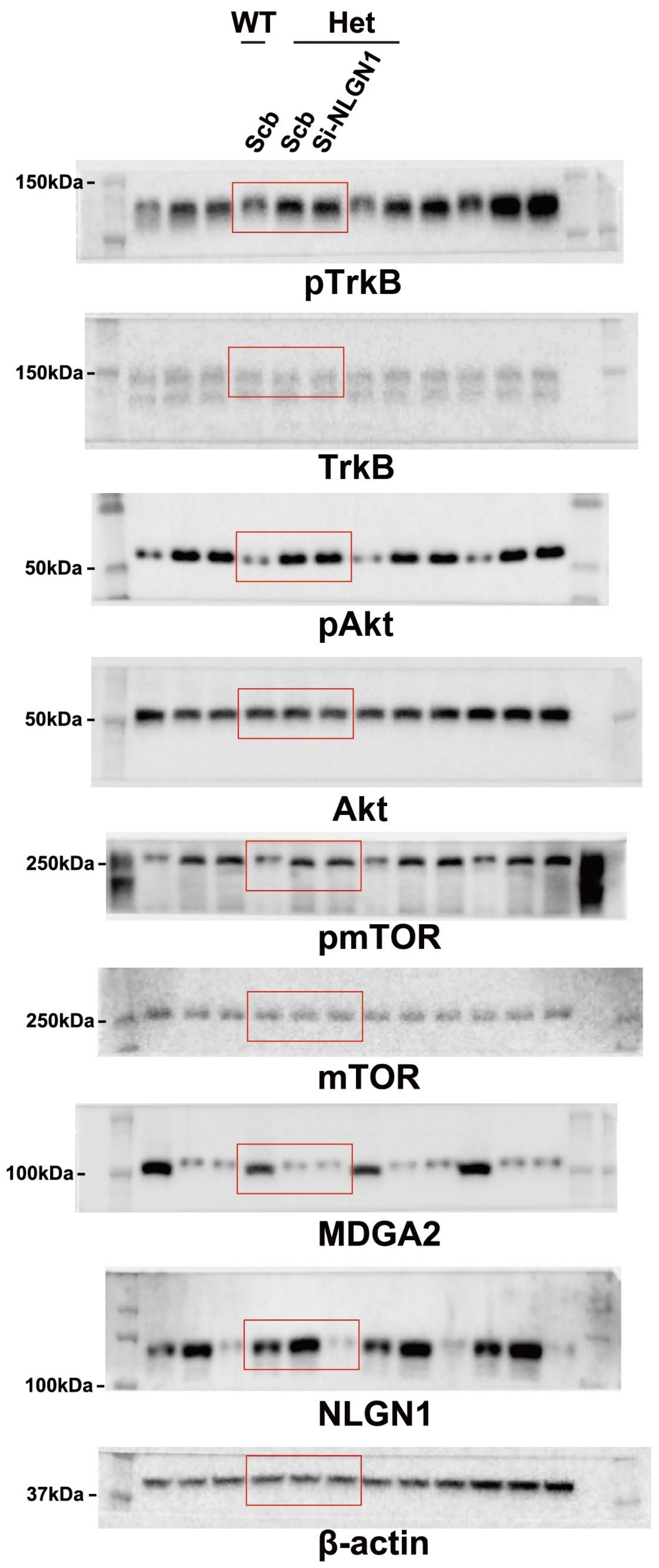

Fig S4A

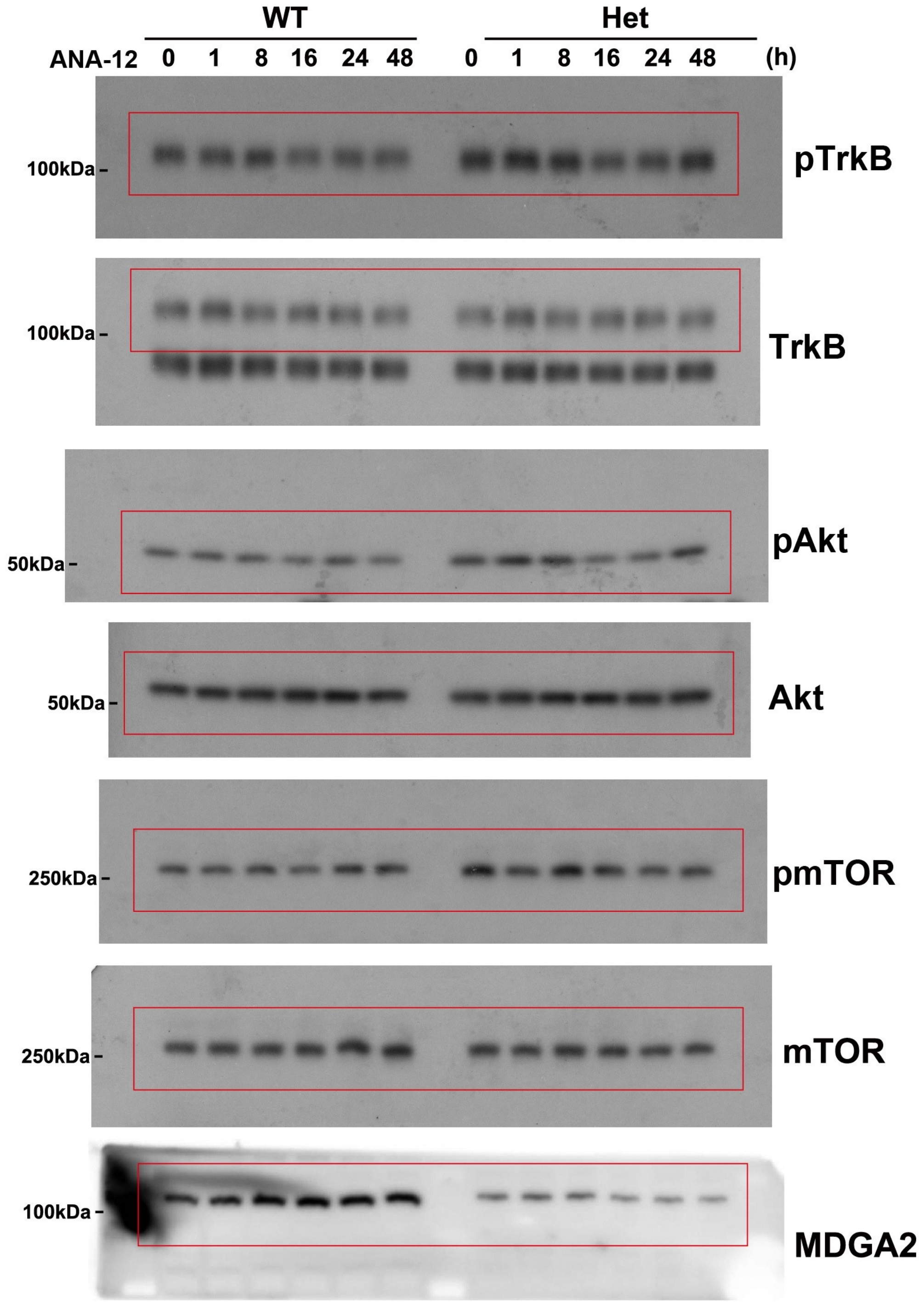

Fig S5G

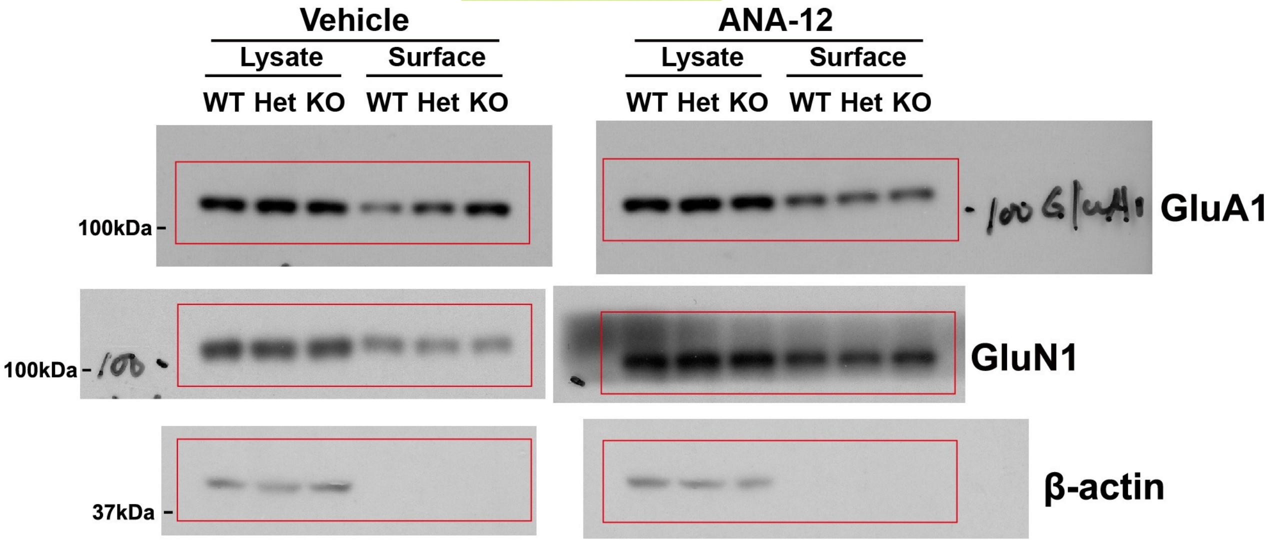

Supplement: S1 Raw Images — (PDF) [file pbio.3003047.s012.pdf]
